# The evolution of the metazoan Toll receptor family and its expression during protostome development

**DOI:** 10.1186/s12862-021-01927-1

**Published:** 2021-11-22

**Authors:** Andrea Orús-Alcalde, Tsai-Ming Lu, Aina Børve, Andreas Hejnol

**Affiliations:** 1 Present Address: Sars International Centre for Marine Molecular Biology, University of Bergen, Thormøhlensgate 55, 5006 Bergen, Norway; 2 Department of Biological Sciences, University of Bergen, Bergen, Norway; 3 Present Address: Institute of Cellular and Organismic Biology, Academia Sinica, Taipei, Taiwan

**Keywords:** Toll receptor, Toll-like receptor, Innate immunity, Development, Metazoan evolution, Gene duplication

## Abstract

**Background:**

Toll-like receptors (TLRs) play a crucial role in immunity and development. They contain leucine-rich repeat domains, one transmembrane domain, and one Toll/IL-1 receptor domain. TLRs have been classified into V-type/scc and P-type/mcc TLRs, based on differences in the leucine-rich repeat domain region. Although TLRs are widespread in animals, detailed phylogenetic studies of this gene family are lacking. Here we aim to uncover TLR evolution by conducting a survey and a phylogenetic analysis in species across Bilateria. To discriminate between their role in development and immunity we furthermore analyzed stage-specific transcriptomes of the ecdysozoans *Priapulus caudatus* and *Hypsibius exemplaris*, and the spiralians *Crassostrea gigas* and *Terebratalia transversa*.

**Results:**

We detected a low number of TLRs in ecdysozoan species, and multiple independent radiations within the Spiralia. V-type/scc and P-type/mcc type-receptors are present in cnidarians, protostomes and deuterostomes, and therefore they emerged early in TLR evolution, followed by a loss in xenacoelomorphs. Our phylogenetic analysis shows that TLRs cluster into three major clades: clade α is present in cnidarians, ecdysozoans, and spiralians; clade β in deuterostomes, ecdysozoans, and spiralians; and clade γ is only found in spiralians. Our stage-specific transcriptome and in situ hybridization analyses show that TLRs are expressed during development in all species analyzed, which indicates a broad role of TLRs during animal development.

**Conclusions:**

Our findings suggest that a clade α TLR gene (*TLR-Ca*) and a clade β/γ TLR gene (*TLR-Cβ/γ*) were already present in the cnidarian-bilaterian common ancestor. However, although *TLR-Ca* was conserved in cnidarians, *TLR-Cβ/γ* was lost during the early evolution of these taxa. Moreover, *TLR-Cβ/γ* duplicated to generate *TLR-Cβ* and *TLR-Cγ* in the lineage to the last common protostome-deuterostome ancestor. *TLR-Ca, TLR-Cβ* and *TLR-Cγ* further expanded generating the three major TLR clades. While all three clades radiated in several spiralian lineages, specific TLRs clades have been presumably lost in other lineages. Furthermore, the expression of the majority of these genes during protostome ontogeny suggests a likely role in development.

**Supplementary Information:**

The online version contains supplementary material available at 10.1186/s12862-021-01927-1.

## Background

Toll-like receptors (TLRs) are involved in immunity and development in metazoans [1–7]. The first described TLR was the *Drosophila* gene *Toll*, which plays a role during early embryonic development [8, 9] and in immunity [10]. The human toll receptor *TLR4* was the first TLR discovered in mammals [11]. Since then, TLRs have been found in most planulozoans (Cnidaria + Bilateria) [12–14]. Both in vertebrates and invertebrates, these receptors recognize pathogens and activate the Toll pathway, which induces the expression of downstream immune genes [15–17]. In *Drosophila*, TLRs are mainly activated by gram-positive bacteria, fungi, and viruses, promoting the synthesis of antimicrobial peptides (AMPs) [4, 10, 17–21]. In vertebrates, TLRs are involved in innate immunity and in the activation and regulation of adaptive immunity [11, 22–26]. TLRs are also involved in the immunity of other animals such as cnidarians [27], mollusks [28–31], annelids [32, 33], crustaceans [34] and echinoderms [35]. The developmental roles of TLRs in *Drosophila* [reviewed in 2] comprise the establishment of the dorso-ventral axis [8, 9], segmentation [36], axis elongation [37], muscle and neuronal development [38, 39], wing formation [40, 41] and heart formation [42]. TLRs also play a role in cnidarian development [27]. Moreover, in ecdysozoans, TLRs are likely involved in onychophoran axis elongation [43]. In spiralians, TLRs are expressed during the development of mollusks [31] and annelids [32], but no further analyses have been conducted. TLRs are also involved in nervous system development in mice [44–47], although the ligands that activate them during this process remain unknown [2].

**Fig. 1:**
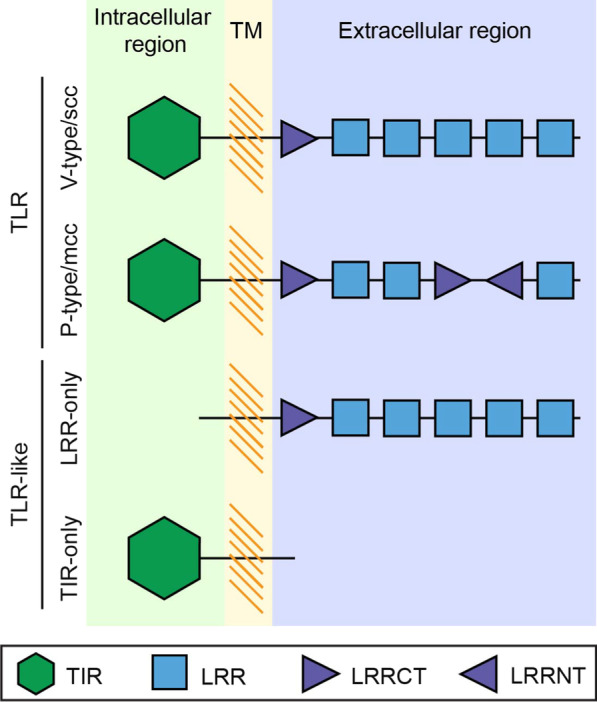
Structure of TLR and TLR-like receptors. TLRs are constituted by a series of extracellular leucine-rich repeat (LRR) domains, a transmembrane region (TM) and an intracellular Toll/IL-1 receptor (TIR) domain. TLRs are often classified into V-type/scc or P-type/mcc according to the structure of their extracellular region. V-type/scc TLRs have only one LRRCT located next to the TIR domain, while P-type/mcc TLRs have more than one LRRCT and, sometimes, an LRRNT domain. Proteins that lack either the LRR domains or the TIR domain are not considered as TLR receptors. These TLR-like proteins are classified in LRR-only or TIR-only. [Adapted from 7, 13]

TLRs are proteins characterized by an extracellular region containing one or more leucine-rich repeat (LRR) domains, one type-I transmembrane domain and one intracellular Toll/IL-1 receptor (TIR) domain (Fig. 1) [48, 49]. The extracellular LRR domains are the regions that recognize the ligand [50, 51]. Each LRR domain is constituted by 22–26 amino acids, in which multiple leucine residues are present [48]. Some LRR domains contain cysteine residues in the N-terminal (LRRNT) or the C-terminal (LRRCT) part of the LRR domain [6, 49, 52]. However, LRR domains are also found in a large number of other proteins [53], for example in the immune NOD receptors [54] and in proteins involved in developmental processes (e.g. Slit, Capricious, Tartan) [55, 56]. The TIR domain is involved in signal transduction [49] and is also present in other proteins, e.g. in immune proteins in plants [57, 58], in members of the interleukin-I receptor family (IL-1) [49, 59] and in adaptors of the Toll pathway (e.g. MyD88) [60–62]. Although the TIR domain is the most characteristic domain of the TLRs, at least one LRR domain must be present to categorize a receptor as TLR (Fig. 1) [13].

Based on the structure of the LRR domains, TLRs have been previously classified as vertebrate-type or single cysteine cluster (V-type/scc), and protostome-type or multiple cysteine cluster (P-type/mcc) (Fig. 1) [7, 13, 63, 64]. V-type/scc TLRs are characterized by having only one LRRCT domain, which is located next to the cellular membrane. P-type/mcc TLRs contain at least two LRRCT domains and, commonly, an LRRNT [7, 13]. Traditionally, it has been assumed that all deuterostome TLRs belong to the V-type/scc [64], and because *Drosophila melanogaster* TLRs (except for Toll9) and the *Caenorhabditis elegans* TLR belong to the P-type/mcc, they have been suggested to be protostome specific [64]. However, P-type TLR are also present in invertebrate deuterostomes and V-type TLRs in protostomes [13, 14, 65, 66]. Therefore, in agreement with Davidson et al. [65]; and Halanych and Kocot [66], we affirm that the V- P-type nomenclature is problematic and should be avoided in favor of the mcc/scc nomenclature.

Several authors consider that TLRs originated in the branch to the Planulozoa by the fusion of a gene with a TIR domain (*TIR-only*) and a gene containing only LRR domains (*LRR-only*) [7, 14, 67]. However, this hypothesis is challenged by the presence of TLRs in choanoflagellates, the sister group to metazoans, which suggests that the origin of TLR could predate metazoans [68]. LRR-only and TIR-only are TLR-like proteins (Fig. 1) involved in immunity [7, 12–14, 69–74]—e.g. in *Hydra*, association of LRR-only and TIR-only proteins activates the Toll pathway [75, 76].

**Fig. 2:**
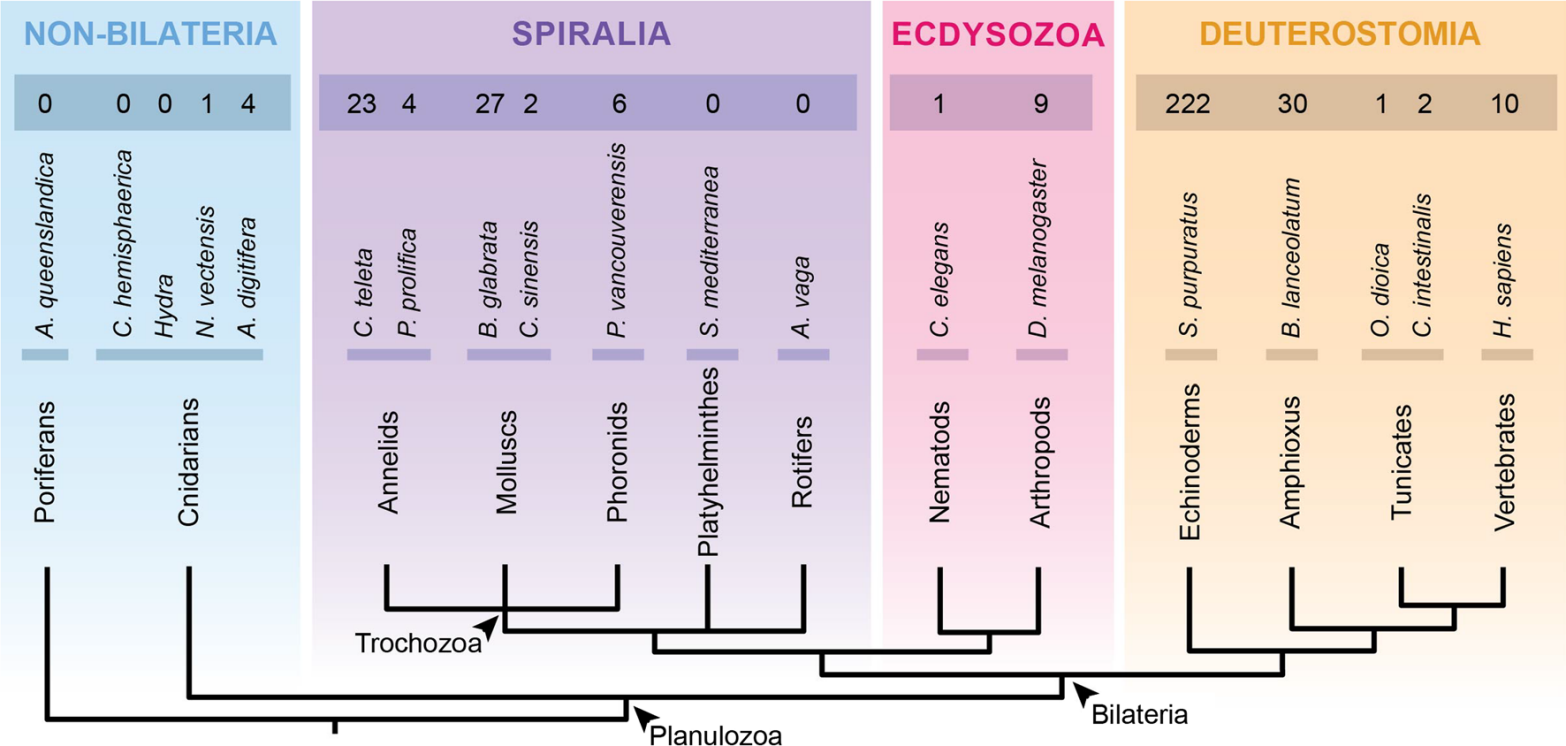
Review of the number of TLRs across metazoans. Within metazoans, no TLRs have been found outside Cnidaria and Bilateria. Spiralians show a variable number of TLRs, being, for example, 23 TLRs in the annelid *C. teleta*, but none in the rotifer *A. vaga*. In ecdysozoans, *C. elegans* and *D. melanogaster* have 1 and 9 TLRs, respectively. The number of TLRs in deuterostomes is also variable, being high in *S. purpuratus* and *B. lanceolatum*, but reduced in tunicates. References: [8, 11, 18, 27, 52, 64–66, 69, 70, 72, 75, 82, 84, 88, 92, 94, 96, 97]. Phylogeny according to [98]

The TLR complement has been previously surveyed in vertebrates [11, 52, 77–79] and in a few invertebrates, especially in arthropods [8, 14, 18, 80, 81]. Humans have 10 TLRs [11, 52], *D. melanogaster* has 9 [8, 18] and the nematode *C. elegans* has only one [82]. Recent genome and transcriptome sequencing of more organisms has revealed that TLRs are widespread across the metazoan tree (summary in Fig. 2). Outside bilaterians, TLRs are present in anthozoan cnidarians (e.g. *Nematostella* [27], *Acropora* [72], *Orbicella* [83]), but not in hydrozoans (e.g. *Hydra* [75], *Clytia* [84]). Furthermore, TLRs have not been found in ctenophores [85, 86], placozoans [73] and poriferans [69, 74]. Within bilaterians, previous studies have shown that the number of TLRs in spiralians is highly variable between species [65, 66, 87–90], suggesting that TLR genes underwent several independent radiations [13, 65, 89, 91]. However, the surveyed platyhelminth and rotifer species lack TLRs [70, 71, 92]. In ecdysozoans, besides arthropods and nematodes, TLRs are also present in onychophorans, tardigrades, nematomorphs and priapulids [93]. In invertebrate deuterostomes, the number of TLRs in echinoderms and amphioxus is expanded [64, 94, 95], which is in contrast to the limited number of TLRs in tunicates [96, 97]. Although the TLR sequences of many metazoans have been explored [7, 12–14], more protostome species must be surveyed to gain a better picture of the TLR evolution (Fig. 2).

Although the phylogenetic relationships of TLRs have been previously analyzed, these were mainly focused on vertebrate TLR evolution [67, 99] or including only few protostome species [13, 65, 89]. So far, the results are contradictory and are not sufficient to comprehend the detailed evolution of TLRs. For instance, Davidson et al. [65] suggested that TLRs are divided into three major clades, although the relationships between them remained unresolved. Brennan and Gilmore [13] suggested that TLRs cluster according to the TLR-type (P-type/mcc or V-type/scc) and Liu et al. [67] suggested that both TLR types would be widespread in invertebrates. Furthermore, Luo et al. [89] showed lineage-specific expansions of TLRs in some trochozoan groups (phoronids, nemerteans and brachiopods). Thus, phylogenetic analyses including TLRs of species representing a broader metazoan diversity are lacking. In this study, we aim to reconstruct the TLR evolution by searching for TLRs in under-represented metazoan clades and performing a phylogenetic analysis including TLRs of species from the four main metazoan clades (cnidarians, spiralians, ecdysozoans and deuterostomes). Moreover, we aim to reconstruct the early TLR function by analyzing their expression during the course of development in four protostome species.

## Results

**Fig. 3:**
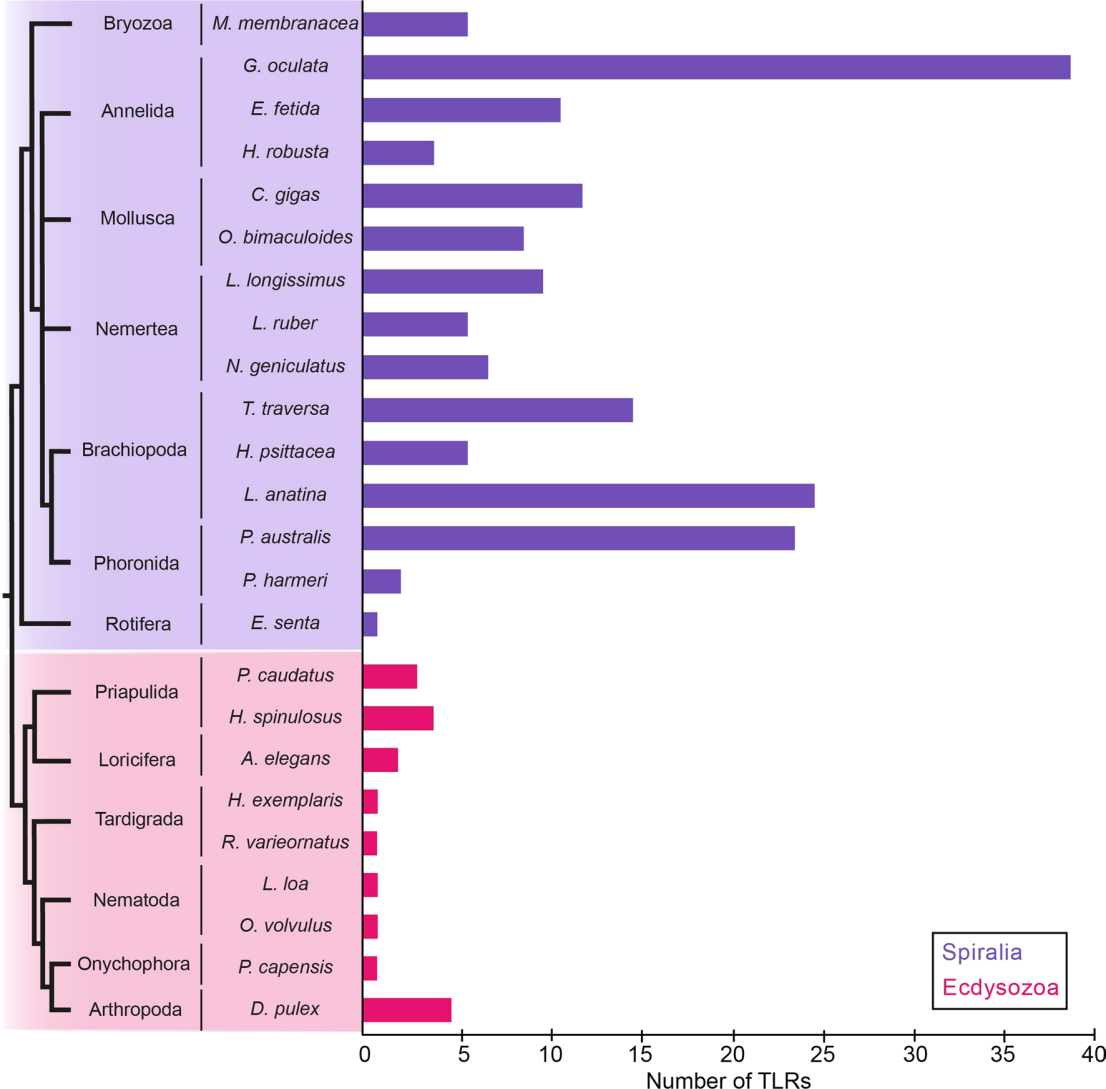
Number of TLRs in species included in the genome/transcriptome analyses. In general, the number of TLRs in spiralians (purple) is higher and more variable between species when compared to ecdysozoans (magenta). Species for which TLRs were not detected are excluded from the graph

Our genome and transcriptomic surveys revealed a total of 198 TLRs in 25 species (Table 1, Fig. 3). No TLRs were found in 20 species. Additionally, our analysis also revealed a large number of TLR-like proteins (TIR-only or LRR-only). However, only sequences containing a TIR domain, a transmembrane domain and, at least, one LRR domain were considered as criteria for TLRs. In this regard, we would like to mention the limitation of our method, as when performing transcriptomic surveys some TLRs could have been under-detected due to no expression in the tissue or the developmental stage sequenced, or because partial sequences obtained from transcriptomes could have been misclassified into TLR-like when an LRR domain, the transmembrane domain and the TIR domain were not present together in one sequence.

**Table 1:** TLR genome/transcriptome survey results and classification of TLRs included in the phylogenetic analysis

Species	TLRs	V-type/scc	P-type/mcc	NC	References
**Cnidaria**					
*Nematostella vectensis*	1	0	1	0	L: [27]
*Acropora digitifera*	4	1	3	0	L: [72]
*Acropora millepora*	1	0	1	0	L: [72]
*Orbicella faveolata*	1	0	1	0	L: [83]
**Xenacoelomorpha**					
*Xenoturbella profunda*	0	0	0	0	G: Unpublished
*Hofstenia miamia*	0	0	0	0	G: GCA004352715
*Praesagittifera naikaiensis*	0	0	0	0	G: PRJDB7329
*Isodiametra pulchra*	0	0	0	0	G: Unpublished
*Meara stichopi*	0	0	0	0	G: Unpublished
*Convolutriloba macropyga*	0	0	0	0	T: [100]
**Spiralia**					
Bryozoa					
*Membranipora membranacea*	6	4	1	1	T: SRX1121923
*Bugula neritina*	0	0	0	0	T: [101]
Cycliophora					
*Symbion pandora*	0	0	0	0	T: [102]
Annelida					
*Galathowenia oculata*	39	18	12	9	T: Unpublished
*Eisenia fetida*	11	0	1	10	T: SRX3108745
*Helobdella robusta*	4	1	3	0	G: [103]
*Phyllochaetopterus prolifica*	3	1	0	2	L: [66]
Mollusca					
*Crassostrea gigas*	12	10	2	0	G: [104]
*Octopus bimaculoides*	9	1	6	2	G: [105]
*Cyclina sinensis*	2	1	1	0	L: [88]
*Leptochiton rugatus*	1	0	0	1	L: [66]
*Biomphalaria glabrata*	27	16	10	1	G: [87]/NCBI
Brachiopoda					
*Terebratalia transversa*	15	4	4	7	T: [100]
*Hemithris psittacea*	6	3	1	2	T: [66]
*Lingula anatina*	25	15	7	3	G: [106]
Micrognathozoa					
*Limnogathia maerski*	0	0	0	0	T: SRX1121929
Gastrotricha					
*Lepidodermella squamata*	0	0	0	0	T: [107]
*Macrodasys sp*	0	0	0	0	T: [108]
*Megadasys sp*	0	0	0	0	T: [108]
*Diuronotus aspetos*	0	0	0	0	T: SRX1121926
*Mesodasys laticaudatus*	0	0	0	0	T: SRX872416
Nemertea					
*Lineus longissimus*	10	7	2	1	T: [100]
*Lineus ruber*	6	2	3	1	T: Unpublished
*Notospermus geniculatus*	7	5	1	1	G: [89]
*Paranemertes peregrina*	2	1	0	1	L: [66]
Phoronida					
*Phoronopsis harmeri*	2	0	1	1	T: SRX1121914
*Phoronis australis*	24	14	8	2	G: [89]
*Phoronis psammophila*	3	1	1	1	L: [66]
*Phoronis vancouverensis*	6	5	0	1	L: [66]
Platyhelminthes					
*Macrostomum lignano*	0	0	0	0	G: [109]
*Echinococcus multilocularis*	0	0	0	0	G: [110]
*Hymenolepis microstoma*	0	0	0	0	G: [110]
Rotifera					
*Epiphanes senta*	1	1	0	0	T: Unpublished
*Rotaria tardigrada*	0	0	0	0	T: [111]
*Echinorhynchus gadi*	0	0	0	0	T: SRX1121912
*Macracanthorhynchus hirudinaceus*	0	0	0	0	T: [108]
**Ecdysozoa**					
Priapulida					
*Priapulus caudatus*	3	0	3	0	T: [100]
*Halicryptus spinulosus*	4	1	3	0	T: [100]
Tardigrada					
*Hypsibius exemplaris*	1	0	1	0	G: [112]
*Ramazzottius varieornatus*	1	0	1	0	G: [113]
Onychophora					
*Peripatopsis capensis*	1	0	0	1	T: [114]
Nematoda					
*Loa loa*	1	0	1	0	G: [115]
*Onchocerca volvulus*	1	0	1	0	G: [116]
*Caenorhabditis elegans*	1	0	1	0	L: NCBI
Loricifera					
*Armorloricus elegans*	2	1	1	0	T: SRX1120677
Arthropoda					
*Daphnia pulex*	5	2	3	0	G: [117]
*Drosophila melanogaster*	9	1	8	0	L: NCBI
*Ixodes scapularis*	5	1	3	1	L: [118]
**Deuterostomia**					
Tunicata					
*Ciona intestinalis*	2	1	1	0	L: [97]
*Oikopleura dioica*	1	1	0	0	L: [96]
Echinodermata					
*Strongylocentrotus purpuratus*	8	7	1	0	L: [64]
Craniata					
*Homo sapiens*	10	10	0	0	L: NCBI

NC column indicates the number of TLRs that could not be classified for each species. In the reference column it is indicated whether the survey was performed in a genome (G) or a transcriptome (T), followed by the reference or NCBI accession number in case the genome/transcriptome was published. TLR sequences extracted directly from the literature and, therefore, were not obtained in our genomic and transcriptomic surveys, are indicated with an L. NCBI indicates that sequences were collected individually from NCBI database. For further details, see Additional file 4: Table S2

### TLRs are absent in the genomes and transcriptomes of xenacoelomorphs and in some spiralians

Our surveys revealed that TLRs are absent in the genomes and transcriptomes of all Xenacoelomorpha, Platyhelminthes, Cycliophora, Micrognathozoa and Gastrotricha species analyzed (Table 1). Furthermore, TLRs are also absent in the transcriptomes of all the rotifer species investigated, except for *E. senta* (Table 1, Fig. 3). Moreover, although TLRs were present in the bryozoan *M. membranacea*, they were not found in the transcriptome of the bryozoan *B. neritina*. However, although TLRs were not detected, TLR-like proteins were present in all these animal groups (data not shown).

### The number of TLRs detected in members of Ecdysozoa is low when compared to Spiralia and Deuterostomia

The TLR survey of the ecdysozoan genomes and transcriptomes revealed only one TLR for the tardigrade, nematode, and onychophoran species analyzed (Table 1, Fig. 3). Furthermore, we detected up to 4 different TLRs in priapulids, 2 in loriciferans, and 5 in arthropods.

### Multiple TLRs are detected in trochozoan species

TLRs were found in the genomes/transcriptomes of all trochozoan species analyzed (Table 1, Fig. 3). Our results reveal that, in general, multiple TLRs are present in highly variable numbers in trochozoan species. The number of TLRs is not reflected by the phylogeny, meaning that species belonging to a same clade do not have a more similar number of TLRs than species belonging to another clade. This is explained by the multiple duplications and losses that have independently occurred in the Toll receptor family during trochozoan evolution [13, 65, 89].

### P-type/mcc and V-type/scc are not specific for any planulozoan clade

Previous studies suggest that V(ertebrate)-type/scc and P(rotostome)-type/mcc TLRs are restricted to vertebrates and protostomes, respectively [64]. However, our results show that both, P-type/mcc and V-type/scc type TLRs, are present in cnidarians, spiralians, ecdysozoans, and deuterostomes (Table 1; Additional file 1: Table S1). V-type/scc TLRs are the most abundant TLR type in the spiralian species analyzed. However, many spiralians also have several P-type/mcc TLRs. P-type/mcc TLRs are the predominant TLR type in the ecdysozoan species included in this analysis. For nematodes, tardigrades and onychophorans, which only have one TLR, this TLR was always classified as P-type/mcc. Ecdysozoan species analyzed with more than one TLR have one or more P-type/mcc TLRs and only one V-type/scc. Although the vertebrate TLR complement seems to only contain V-type/scc TLRs [14, 67, 119, 120], P-type/mcc TLRs are also present in other deuterostomes, such as the tunicate *C. intestinalis* [97] and the echinoderm *S. purpuratus* [64] (Table 1, Additional file 1: Table S1). This suggests that P-type/mcc TLRs were lost in the lineage to the Craniata.

### TLRs form three clades

**Fig. 4:**
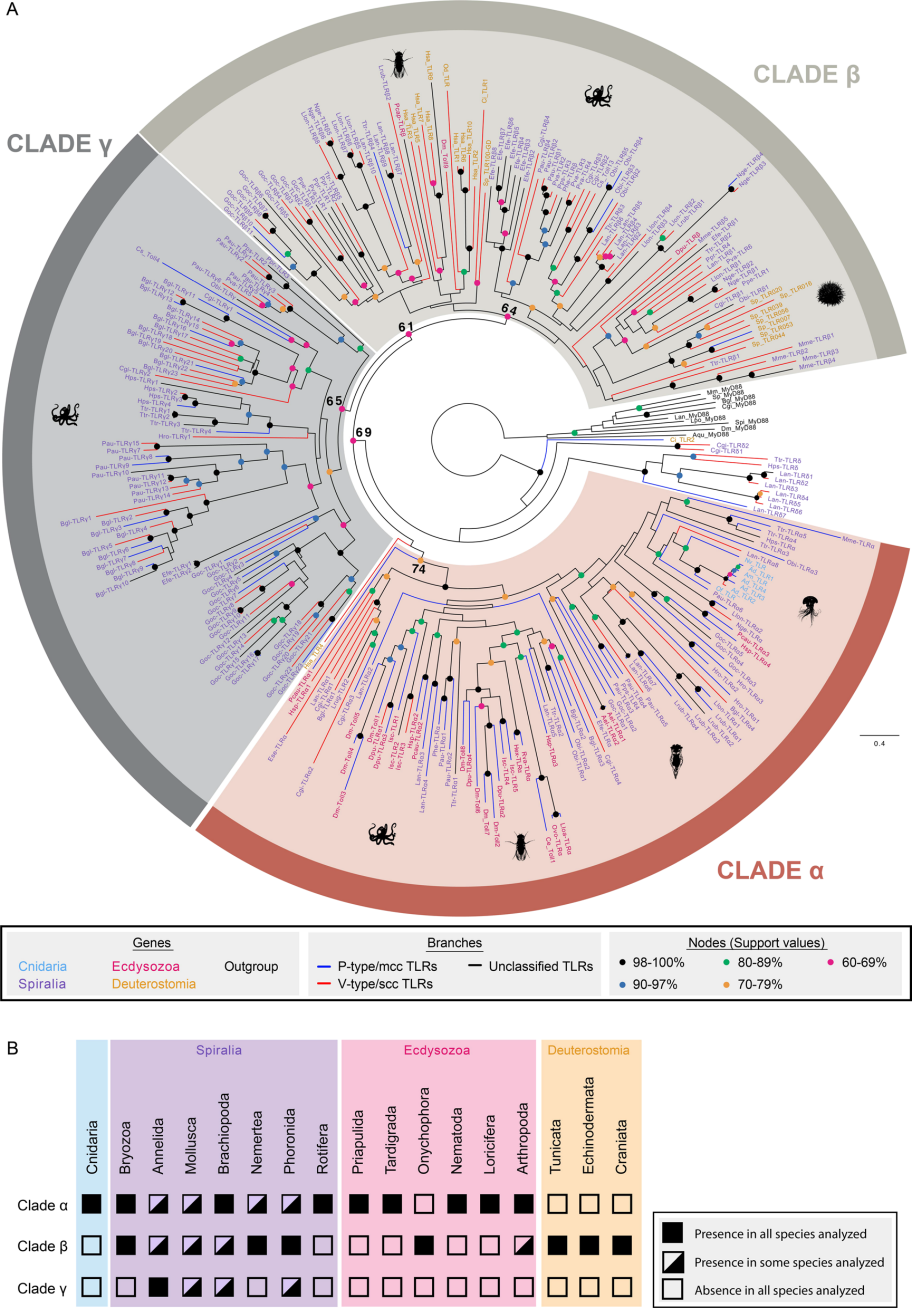
TLR phylogenetic analysis and distribution of P-type/mcc or V-type/scc. **A** Phylogenetic analysis of TLRs based on maximum likelihood. Bootstrap values are indicated next to the main nodes, being all nodes with bootstrap values > 60 marked with full dots and colored differently according to the support values. Tip labels contain an abbreviation of the species name and the gene name given in this study (for sequences searched de novo here) or in the original study (for sequences obtained from the literature). Numbers in the gene name do not imply gene orthology. Species abbreviations: Ael: *A. elegans*; Ad: *A. digitifera*; Am: *A. millepora*; Bgl: *B. glabrata*; Ce: *C. elegans*; Cgi: *C. gigas*; Ci: *C. intestinalis*; Cs: *C. sinensis*; Dm: *D. melanogaster*; Dpu: *D. pulex*; Efe: *E. fetida*; Ese: *E. senta*; Goc: *G. oculata*; Hex: *H. exemplaris*; Hps: *H. psittacea*; Hro: *H. robusta*; Hsa: *H. sapiens*; Hsp: *H. spinulosus*; Isc: *I. scapularis*; Mme: *M. membranacea*; Nge: *N. geniculatus*; Nv: *N. vectensis*; Lan: *L. anatina*; Lloa: *L. loa*; Llon: *L. longissimus*; Lrub: *L. ruber*; Lrug: *L. rugatus*; Obi: *O. bimaculoides*; Od: *O. dioica*; Of: *O. faveolata*; Ovo: *O. volvulus*; Pau: *P. australis*; Pcap: *P. capensis*; Pcau: *P. caudatus*; Phe: *P. hermeri*; Ppe: *P. peregrina*; Ppr: *P. prolifca*; Pps: *P. psammophila*; Pva: *P.vancouverensis*; Rva: *R. varieornatus*; Sp: *S. purpuratus*; Ttr: *T. transversa*. **B** Presence/absence of the TLR clades in the metazoan groups included in our study

Our phylogenetic analysis showed that TLRs group into three clades (Fig. 4A), which we named clade α (89 TLRs), clade β (102 TLRs) and clade γ (79 TLRs). Although these three clades are supported with support values > 60, some of the internal nodes have low support values (< 60). The phylogenetic analysis showed that clades β and γ are sister clades and together form the sister group to clade α. All three clades contain both P-type/mcc and V-type/scc TLRs, which makes it difficult to reconstruct whether P-type/mcc or V-type/scc show the ancestral state of TLRs. Furthermore, 2 deuterostome TLRs (from *H. sapiens* and *C. intestinalis*) and 11 spiralian TLRs (2 from species of mollusks and 9 from brachiopods) could not be assigned to any of the above clades. The 9 brachiopod TLRs form a clade with a high support value (> 60), but do not group with either the mollusk or the deuterostome sequences. This TLR brachiopod clade is the sister clade to the three main clades (α, β and γ). For these sequences, the alignment showed brachiopod-specific deletions in the amino acid positions 150–220 that are not present in the TLRs belonging to the three main clades (Additional file 2: Fig. S1). To investigate whether this insertion is causing the clustering of the TLRs into three clades, we performed a second phylogenetic analysis (Additional file 3: Fig. S2) with the same parameters of the main analysis (Fig. 4A) but excluding the 150–200 amino acid region. The second analysis (Additional file 3: Fig. S2) is able to reconstruct clade α with high support value (> 60). However, clade γ is nested within clade β and both of them have low support values (< 60). In the second analysis (Additional file 3: Fig. S2), as in the main analysis (Fig. 4), the 9 brachiopod sequences cluster together and form the sister clade to the three main clades. However, in the analysis shown in Additional file 3: Fig. S2, the mollusk and deuterostome sequences are included in the clade γ. In the main analysis (Fig. 4A), no distinctive motifs were observed in the alignment that justify the exclusion of these sequences from the main clades.

Clade α includes TLRs from all cnidarian, spiralian and ecdysozoan species analyzed, except for the onychophoran TLR (Fig. 4). Because all cnidarian TLRs cluster together, it is likely that only one TLR was present in the last common ancestor of Cnidaria. Clade β is formed by TLRs belonging to deuterostomes, spiralians and three ecdysozoans (two arthropods and the onychophoran TLR) (Fig. 4). This suggests that at least the ancestral TLR of Clade β/γ was already present in the last common ancestor of Nephrozoa (Protostomia + Deuterostomia). Furthermore, lineage-specific expansions of clade β TLRs are detected in spiralians and deuterostomes. Clade γ TLRs are present in all trochozoan groups except for the nemertean species analyzed (Fig. 4). Clade γ contains TLRs that radiated independently in several lineages. Our alignment shows that 159/181 TLRs belonging to the clades β and γ contain an insertion of 6 amino acids in the positions 349–354 (Additional file 2: Fig. S1). In Clade α, this insertion is only present in Pcau-TLRα1, the sister TLR to all the remaining TLRs belonging to this clade. To exclude that this insertion causes the clustering in three distinct clades, we performed a third phylogenetic analysis (Additional file 5: Fig. S3), in which we applied the same parameters as in the main analysis -shown in Fig. 4A- but eliminated the 6 amino acid insertion regions. In the third analysis (Additional file 5: Fig. S3), the three clades could be reconstructed with good support values (> 60). However, due to low support values (< 60), the relationship between the clades could not be resolved. Moreover, the clustering of the TLRs into the three clades (α, β, γ) was maintained with respect to the main analysis (Fig. 4A, Additional file 5: Fig. S3), except for eight phoronid and one human sequences. In the main analysis (Fig. 4A), the phoronid sequences cluster together within clade γ, with high support values (> 60). This clade of phoronid TLRs is the sister clade to all remaining TLRs in clade γ. Nevertheless, in the third analysis (Additional file 5: Fig. S3), these phoronid TLR sequences constitute a well-supported (> 60) clade within clade β, but it is not the sister clade to the remaining TLRs in this clade. In the main analysis (Fig. 4A), the human sequence is not included in any of the three main clades, but in the third analysis (Additional file 5: Fig. S3) it does cluster in clade α.

### TLRs are expressed during development in the ecdysozoans *P. caudatus* and *H. exemplaris* and in the spiralians *C. gigas* and *T. transversa*

In order to study the temporal expression of TLRs during ontogeny, we analyzed stage-specific transcriptomes of the priapulid *P. caudatus* [121], the tardigrade *H. exemplaris* [122], the mollusk *C. gigas* [104] and the brachiopod *T. transversa* [123]. All the analyses were performed using both RSEM [124] and kallisto [125] methods.

**Fig. 5:**
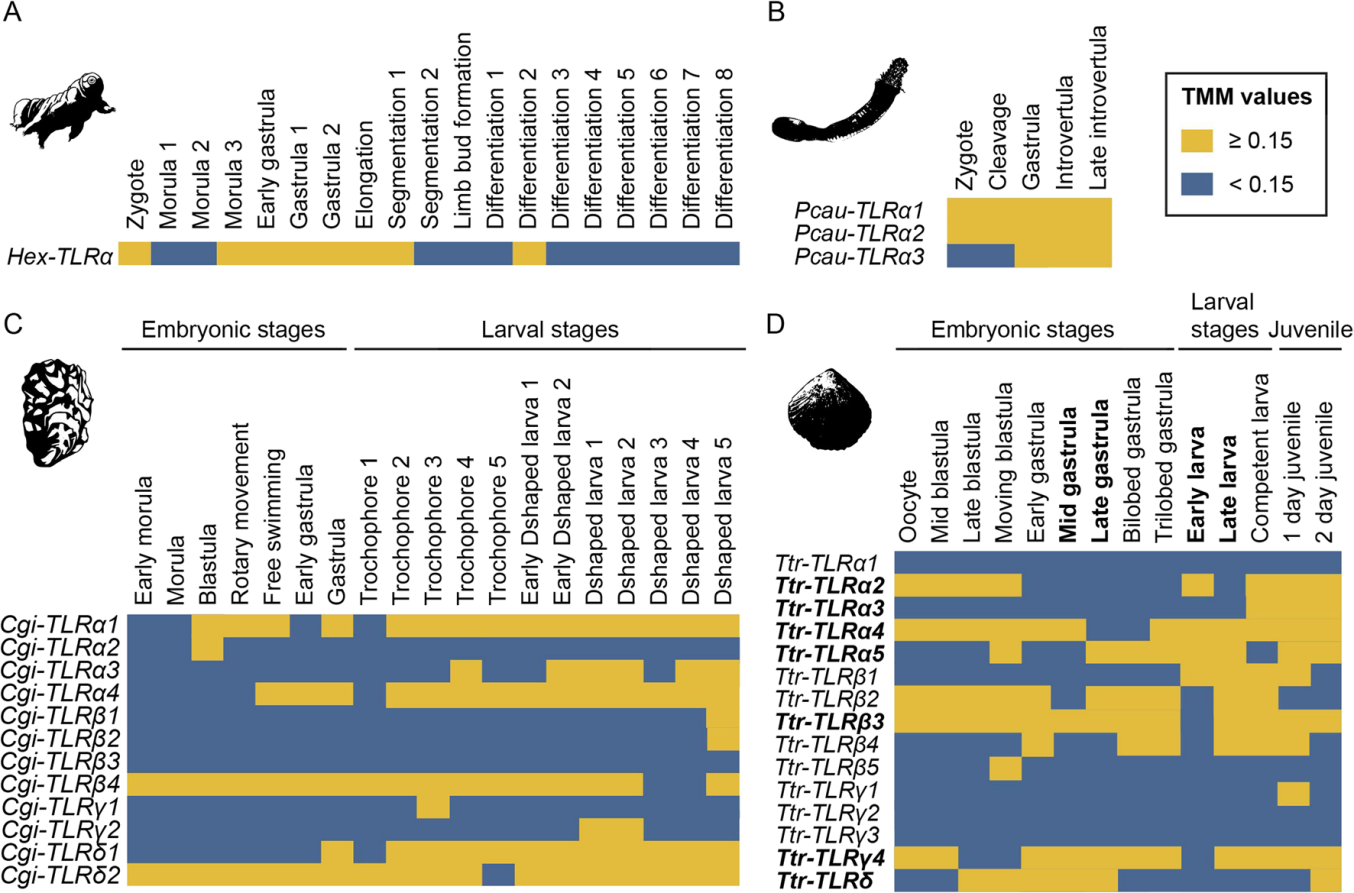
TLR expression in developmental stage-specific transcriptomes of (**A**) *H. exemplaris*, (**B**) *P. caudatus*, **C** *C. gigas* and (**D**) *T. transversa*. Heatmaps corresponding to the average of the RSEM analyses are shown. For heatmaps corresponding to Kallisto analyses see Additional files 6, 7, 8 and 9: Tables S3–S6. Bold indicates stages and genes for which in situ hybridization was performed. TMM: Trimmed means of M values

The expression of the only TLR present in *H. exemplaris* was analyzed in stage-specific transcriptomes of 19 stages (one biological replicate) (Fig. 5A; Additional file 6: Table S3) [122]. Expression of *TLRα* was detected (TMM ≥ 0.15) in time windows during development (zygote, morula, gastrula, elongation, segmentation and differentiation).

Three TLRs were identified in *P. caudatus* transcriptomic survey (Table 1). The expression of these TLRs was analyzed in five embryonic stages (two biological replicates) (Additional file 7: Table S4) [121]. Our results indicate that all three TLRs found in the transcriptomic survey are expressed during embryonic development (TMM ≥ 0.15). *Pca-TLRα1* and *Pca-TLRα2* are expressed in all developmental stages analyzed, whereas *Pca-TLRα3* is expressed only in the later embryonic stages (Fig. 5B; Additional file 7: Table S4).

The expression of the 12 *C. gigas* TLRs (Table 1) was analyzed in stage-specific transcriptomes of 19 stages (one biological replicate) (Additional file 8: Table S5) [104]. Our results show that at 11 of the 12 TLRs are expressed during development (Fig. 5C; Additional file 8: Table S5). Some TLRs are expressed throughout development (*Cgi-TLRα1, Cgi-TLRα4, Cgi-TLRβ4, Cgi-TLRδ1, Cgi-TLRδ2*), while others (*Cgi-TLRα2, Cgi-TLRα3, Cgi-TLRβ1, Cgi-TLRβ2, Cgi-TLRγ1, Cgi-TLRγ2*) are only expressed at certain developmental stages. *Cgi-TLRβ3* expression was not detected at any of the stages analyzed.

15 TLRs were found in our transcriptome survey of *T. transversa* (Table 1). Expression of these TLRs was analyzed in stage-specific transcriptomes of 12 developmental stages (with two biological replicates) [123]. Our results suggest that at least 12 of the 15 TLRs are expressed at certain stages during *T. transversa* development (Fig. 5D; Additional file 9: Table S6)*. Ttr-TLRα2, Ttr-TLRα5, Ttr-TLRβ1, Ttr-TLRβ4, Ttr-TLRβ5,* and *Ttr-TLRδ* expression is detected in time windows during embryonic and larval stages. All these genes, except *Ttr-TLRβ5*, are expressed in juveniles. For some genes (*Ttr-TLRα4, Ttr-TLRβ2, Ttr-TLRβ3,* and *Ttr-TLRγ4*), expression was detected throughout development. Moreover, expression was not detected at the embryonic and larval stages analyzed for *Ttr-TLRα1, Ttr-TLRγ1, Ttr-TLRγ2* and *Ttr-TLRγ3*. Similarly, *Ttr-TLRα3* expression was only detected in the competent larvae and in the juveniles.

Our analyses show that TLRs are expressed during the development of the spiralians *T. transversa* and *C. gigas* and the ecdysozoans *P. caudatus* and *H. exemplaris*. These analyses show that the TLRs expressed during development are not restricted to one TLR clade in the tree shown above, but they are found in all three main clades (e.g. *Ttr-TLRα4, Ttr-TLRβ3, Cgi-TLRγ1*).

**Fig. 6:**
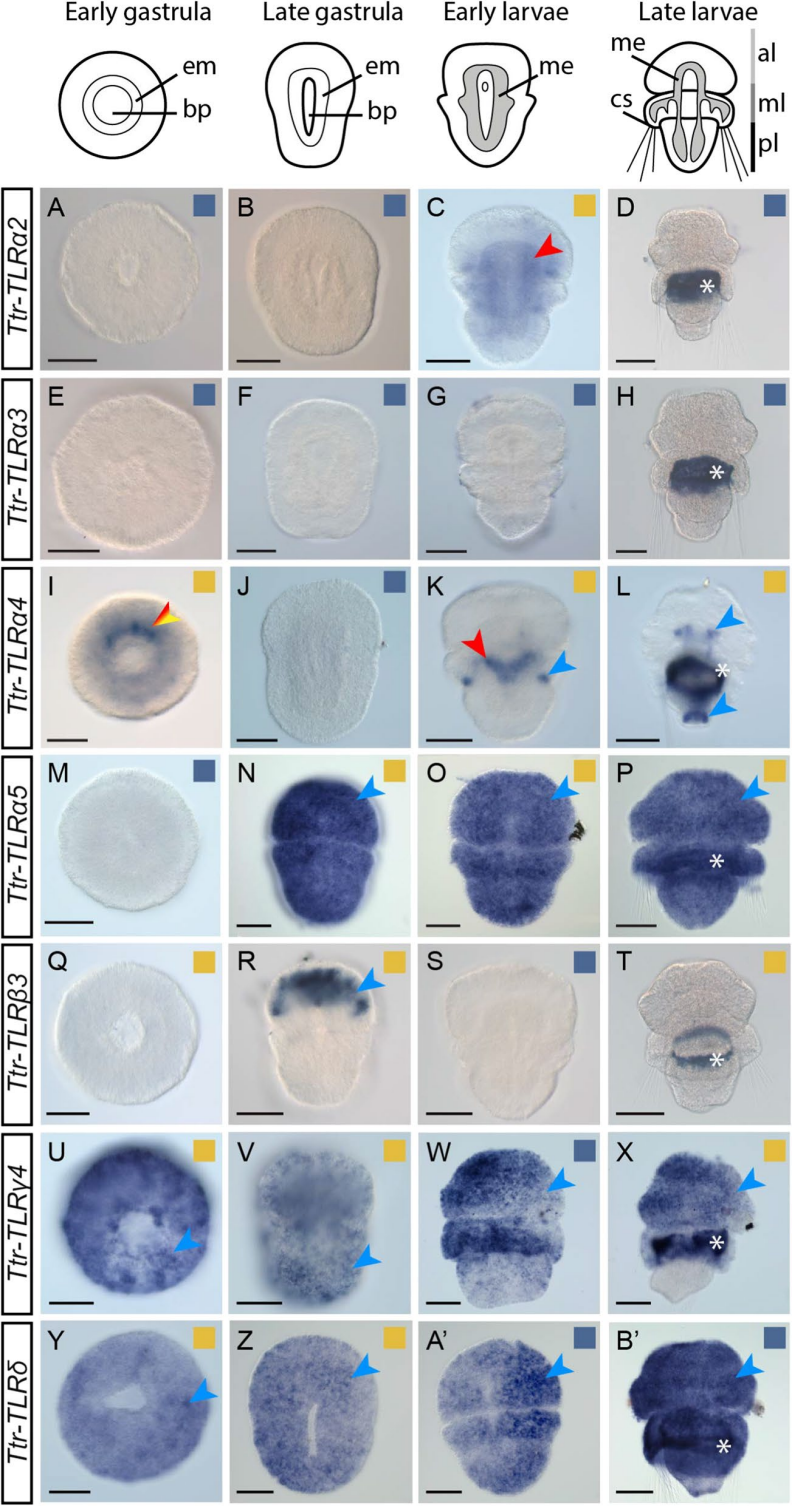
Expression of TLRs during the development of the brachiopod *T. transversa*. Whole-mount in situ hybridization (WMISH) of TLRs in *T. transversa* embryos and larvae. Above the WMISH plates, there are schematic representations of each developmental stage analyzed. These representations are not to scale. The name of each gene is indicated in the rectangles on the left. All panels show dorso-ventral views and anterior to the top. Squares in the top-right of each plate indicate whether the expression was detected (yellow) or not (blue) in the stage-specific transcriptome analysis. Ectoderm, mesoderm and endoderm is indicated with blue, red and yellow arrowheads, respectively. The red and yellow arrowhead indicates endomesoderm. The ring-shape (indicated with white asterisks) staining present in the late larvae is background staining (Additional file 10: Fig. S4), probably related with the spicule formation described by Stricker and Reed [126–128]. Scale bar indicates 50 μm. al: apical lobe; bp: blastopore; cs: chaetal sacs; em: endomesoderm; me: mesoderm; ml: mantle lobe; pl: pedicle lobe

Furthermore, in order to validate our stage specific transcriptome results, we performed whole mount in situ hybridization (WMISH) for the *T. transversa* mRNAs of *TLRα2, TLRα3, TLRα4, TLRα5, TLRβ3, TLRγ4* and *TLRδ* (Fig. 6). Consistently with our stage specific transcriptomic analysis, our WMISH results show that *Ttr-TLRα2* is not expressed at the early and late gastrula stages (Fig. 6A,B), but the expression is present in the mesoderm and in two pairs of lateral domains in early larvae (Fig. 6C). This gene is not expressed in late larvae (Fig. 6D). In agreement with our stage specific transcriptomic analysis, we did not detect *Ttr-TLRα3* neither in the gastrula nor in the larval stages analyzed (Fig. 6E–H). *Ttr-TLRα4* has a dynamic expression pattern during *T. transversa* development. This gene is expressed in the mesoderm at the early gastrula stage, but, consistent with the stage specific transcriptome analysis, it is not detected in late gastrulae (Fig. 6I–J). In early larvae, *Ttr-TLRα4* is expressed in the inner lobe epithelium and in a medial V-shaped mesodermal domain (Fig. 6K). In late larvae, this gene is expressed in the brain and in the pedicle (Fig. 6L). *Ttr-TLRα5* is not expressed at early gastrula stage (Fig. 6M), however, mRNA of *Ttr-TLRα5* is detected in a uniform salt and pepper distribution at the late gastrula stage and the two larval stages for which WMISH was performed (Fig. 6N–P). Furthermore, although *Ttr-TLRβ3* expression was detected in early gastrula in the stage specific transcriptome analysis, expression was not detected by WMISH (Fig. 6Q). However, this gene is expressed in the anterior region of the animal in late gastrulae (Fig. 6R). Moreover, WMISH shows no expression of *Ttr-TLRβ3* in the early larvae (Fig. 6S). However, similarly to the early gastrula stage, *Ttr-TLRβ3* expression was detected in late larvae in the stage specific transcriptome analysis, but its expression was not detected by WMISH (Fig. 6T). The expression of *Ttr-TLRγ4* and *Ttr-TLRδ* have a uniformly salt and pepper distribution at the gastrulae and early larvae stages (Fig. 6U–W and Y–A’). This salt and pepper transcript distribution is similar in late larvae, although it is absent from the pedicle lobes (Fig. 6X and B'). These results conflict with the stage specific transcriptome analyses, as, in this analysis, neither *Ttr-TLRγ4* expression was detected in the early larvae nor *Ttr-TLRδ* in any of the two larval stages tested. Differences between the results of both analyses could be explained by differences and variation of the developmental stages of the specimens used for the stage-specific transcriptome and the WMISH.

## Discussion

### The evolution of the TLR family is characterized by losses, expansion and conservation

As shown in previous studies, TLRs are absent in the Platyhelminthes *S. mediterranea* and *S. mansoni* [92]. Here, we show that this receptor family is also absent from the genomes of three other platyhelminth species (*M. lignano, E. multilocularis* and *H. microstoma*). Thus, TLRs are absent in species belonging to four different platyhelminth lineages (Macrostomorpha—*M. lignano*; Cestoda—*E. multiocularis* and *H. microstoma*; Tricladida—*S. mediterranea*; and Digenea—*S. mansoni*) suggesting that TLRs could have been lost during early platyhelminth evolution. This hypothesis is reinforced by the lack of TLRs in *M. lignano*, member of Macrostomorpha, an early-diverging platyhelminth lineage [107]. In rotifers, even though TLRs could not be detected in *A. vaga* [70], *E. gadi, R. tardigrada* and *M. hirudinaceus*, our transcriptome survey revealed one TLR in the monogonont rotifer *E. senta*. This suggests that TLRs would have been independently lost in some rotifer lineages. So far, we did not detect TLRs in the genomes and transcriptomes of the species belonging to Xenacoelomorpha, Cycliophora, Micrognathozoa, and Gastrotricha, suggesting that TLRs were lost in these lineages. How the immune response is achieved in animals that lack TLRs is unknown, but it could be triggered by other components of the Toll pathway - e.g. TLR-like molecules [14, 70–72] -, similar to what has been shown for LRR-only TLR-like and TIR-only TLR-like in *Hydra* [75, 76].

Another outcome of this study is the remarkable expansion that the TLRs family exhibits in trochozoans. Evolution of this gene family in trochozoans is characterized by multiple duplications and losses, having as a consequence a very variable number of the TLRs complement in trochozoans. Moreover, in our phylogenetic analysis, TLRs of the same species and clades mostly group together, indicating the existence of multiple independent duplications (Fig. 4A). The same has been shown also in previous phylogenetic analyses of TLRs [13, 65, 89].

In contrast to trochozoans, our results show that the number of TLR in ecdysozoans has been relatively conserved during evolution. At least, few TLR gene duplications have occurred in this lineage, including recent independent duplications in arthropods, priapulids or loriciferans.

### The evolution of the three clades (α, β, γ) of TLRs

**Fig. 7:**
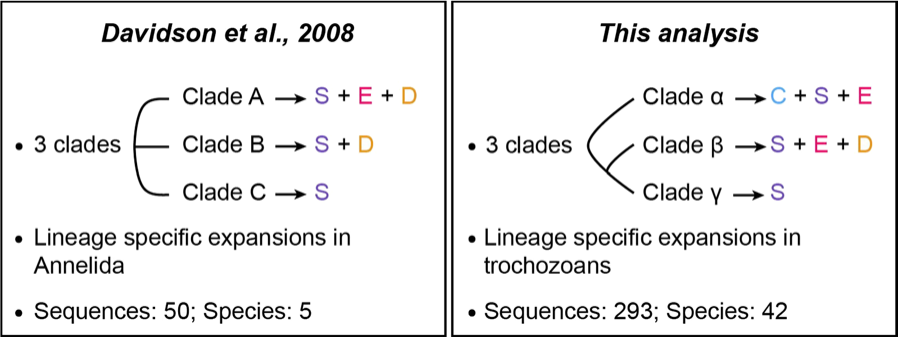
Comparison between Davidson et al. [65] and this study. The main conclusions and the number of TLRs and species included in the two studies are compared. Cnidaria (C), Spiralia (S), Edysozoa (E) and Deuterostomia (D)

There are very few studies assessing the phylogenetic relationships of TLRs within the main metazoan clades (Fig. 7) [65, 89]. The study of Davidson et al. [65] recovered three clades of TLRs. However, the relationships between the clades remain unclear. Furthermore, the composition of the clades slightly differs in both analyses (e.g. while our study shows that deuterostome TLRs belong to one clade—clade β—their results suggest that deuterostome TLRs are present in two clades—clades A and B) [65]. However, their phylogenetic study is limited by the number of sequences and species included. Similar to Luo and Zheng [129]; and Luna et al. [130], our results suggest that ecdysozoan and deuterostome TLRs evolved independently from a common TLR precursor. However, our phylogenetic analysis has also some limitations, as the support values for the main clades are not optimal (with support values 61–74%). This is also reflected by the rearrangement of the tree when the alignment is modified for the phylogenetic analyses shown in Additional files 2, 3: Figs. S2 and S3.

Previous studies suggest that TLRs originated likely by the fusion of an *LRR-only* and a *TIR-only* TLR genes in the branch to the Planulozoa (Cnidaria + Bilateria) [7, 14, 67]. However, this hypothesis is challenged by the presence of TLRs in choanoflagellates, indicating that at least one TLR could be already present in the common ancestor of choanoflagellates and animals [68].

**Fig. 8:**
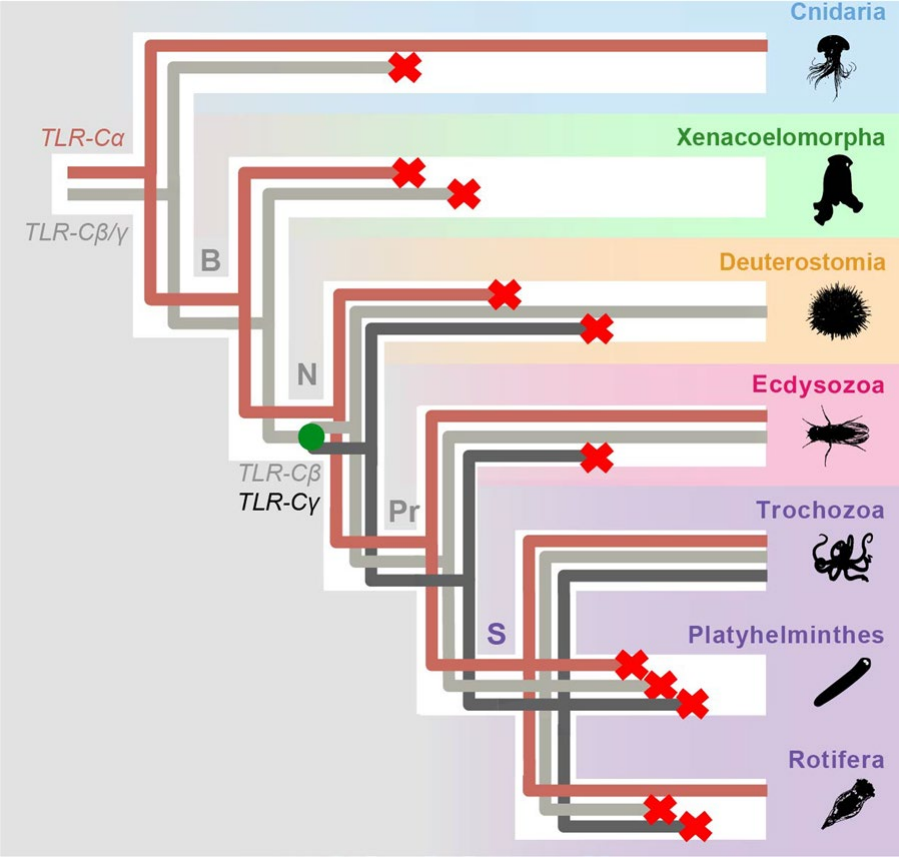
Origin and evolution of TLRs. Gene lineages are depicted in different colors (*TLR-Cα*: light brown; *TLR-Cβ/γ* and *TLR-Cβ*: light grey; and *TLR-Cγ*: dark grey) within the metazoan tree. Gene losses are indicated with a cross. Phylogeny according to: [98]. For other hypotheses see Additional file 11: Fig. S5

Our data suggests different hypothesis on how TLRs evolved within the animal lineages (Fig. 8, Additional file 11: Fig. S5). The main differences between these hypotheses reside in when the duplications that originated the ancestral genes for the TLR α, β and γ clades (*TLR-Cα, TLR-Cβ, and TLR-Cγ*, respectively) ocurred. First, from our data, we can hypothesize that there were present either one TLR (a *TLR-Cα/β/γ*) in the planulozoan common ancestor—hypothesis 1A -; while it could be also inferred that two TLRs (*TLR-Cα* and *TLR-Cβ/γ*) were already present in the planulozoan common ancestor and *TLR-Cβ/γ* was lost in cnidarians—hypothesis 1B -. Since cnidarian TLRs are well nested within clade α (Fig. 4A)*,* we suggest that the most probable scenario is that the planulozoan common ancestor had already a *TLR-Cα* and a *TLR-Cβ/γ*—hypothesis 1B -. Second, according to our analyses, the duplication of *TLR-Cβ/γ* that gave raise to *TLR-Cβ* and *TLR-Cγ* could have occurred either in the nephrozoan common ancestor—hypothesis 2A -, the spiralian common ancestor—hypothesis 2B—or the trochozoan common ancestor—hypothesis 2C -. Since TLR clade γ is not nested within clade β, we suggest that the emergence of *TLR-Cβ* and *TLR-Cγ* probably occurred in the nephrozoan common ancestor—hypothesis 2A -. The only possibility for clade β and γ to be sister clades (Fig. 4A) but have emerged after the split of deuterostome and protostome lineages would be if only one *TLR-Cβ/γ* would be present until the point of the splitting event. Since duplications and losses have occurred frequently during TLR evolution, we suggest that the most probable scenario is that *TLR-Cβ* and *TLR-Cγ* emerged in the nephrozoan common ancestor. In order to see other hypotheses for TLR evolution, see Additional file 11: Fig. S5.

Therefore, here we hypothesize that the planulozoan stem species already had two TLRs (Fig. 8, Additional file 11: Fig. S5B): a clade α type TLR gene (*TLR-Cα*) and a *proto*-TLR gene of clades β and γ (*TLR-Cβ/γ*). This is supported by the fact that all cnidarian TLRs included in our analysis cluster in a monophyletic group within clade α, which is consistent with the results of Brennan and Gilmore [13]. During cnidarian evolution, this gene was lost in some lineages, e.g. *Hydra* [75], *Clytia* [84], and multiplied in others, e.g. *A. digitifera* [72]. Furthermore, under this scenario, *TLR-Cβ/γ* was presumably lost during early cnidarian evolution, as TLRs belonging to this clade are absent in all extant cnidarian analyzed. Moreover, after the split into the Xenacoelomorpha and the Nephrozoa lineages, both *TLR-Cα* and *TLR-Cβ/γ* were lost in xenacoelomorphs.

Before the split into the deuterostome and the protostome lineages, *TLR-Cβ/γ* was duplicated in the lineage to the nephrozoan common ancestor, giving raise to the *TLR-Cβ* and *TLR-Cγ* genes (Fig. 8). However, our results indicate that both *TLR-Cα* and *TLR-Cγ* were lost during early deuterostome evolution. Later, expansions of *TLR-Cβ* generated the TLR diversity found in deuterostomes. Furthermore, as vertebrate TLRs diversified within the vertebrate lineage, it is impossible to make one-to-one orthology gene assignments between the vertebrate TLRs and the invertebrate TLRs [67].

Similarly than the nephrozoan common ancestor, the protostome stem species and the spiralian stem species had likely at least one TLR belonging to each clade (Fig. 8). Our results show that only TLRs belonging to clades α and β are present in ecdysozoans (Fig. 4), suggesting that *TLR-Cγ* was probably lost during early ecdysozoan evolution. Although, in general, the number of TLRs in ecdysozoans is low, few duplications of *TLR-Cα* occurred in some lineages (e.g. arthropods, priapulids, loriciferans). Furthermore, our analysis shows that the surveyed priapulids, tardigrades, nematodes and loriciferan lack TLRs from clade β; whereas clade β TLRs are present in the majority of the arthropods and in the onychophoran surveyed. This would imply that TLR clade β would have been lost several times independently in the early branches and preserved in the other lineages [98, 131].

Within Spiralians, the evolution of TLRs followed different strategies in trochozoan and non-trochozoan organisms. In trochozoans, TLRs from the three clades were maintained (Fig. 8), followed by episodes of gene duplication that generated the large diversity of TLRs. These expansions could have occurred in correlation with the necessity to adapt to microbe rich environments [132, 133]. Nonetheless, all TLRs were lost in other non-trochozoan organisms (e.g. platyhelminthes). Losses of TLR β and γ also occurred in rotifers, although a TLRα gene is present in the monogont rotifer *E. senta*.

### Are protostome TLRs involved in immunity and development during ontogeny?

TLRs are well known to play a key role in adult innate immunity in planulozoans [11, 22–26]. During ontogeny, this gene family has also been shown to be involved in a great number of developmental processes both in arthropods and vertebrates [2, 8, 9, 36, 38, 39, 42, 44–46]. Here, we identify TLRs expressed during ontogeny in four protostome species (the ecdysozoans *H. exemplaris* and *P. caudatus* and the spiralians *C. gigas* and *T. transversa*) (Figs. 5 and 6). Expression of TLRs was observed for some TLRs in short developmental time windows (the *H. exemplaris Hex-TLRα*; the *C. gigas Cgi-TLRα2, Cgi-TLRα3, Cgi-TLRβ1, Cgi-TLRβ2, Cgi-TLRγ1, Cgi-TLRγ2*; and the *T. transversa Ttr-TLRα2, Ttr-TLRα5, Ttr-TLRβ1, Ttr-TLRβ4, Ttr-TLRβ5*), suggesting a possible role of these genes in development, as genes involved in developmental processes are usually expressed for defined periods of time in tissues in order to participate in specific developmental processes [134–136]. For instance, expression during early embryonic stages of the *T. transversa Ttr-TLRα2* (Fig. 5) might suggest its involvement in dorso-ventral axis specification, as it has been shown for the *Drosophila Toll* [8, 9]. Later, in the early larvae, transcription of this gene is transiently activated in the mesoderm (Figs. 5 and 6), suggesting that this gene might be also involved in mesoderm development. However, our analyses do not exclude the possibility that these genes might also be involved in immunity, as these TLRs could have a dual role, as it has been shown for the *Drosophila Toll* [10] and the only TLR in the cnidarian *N. vectensis* [27]. Discerning the role of TLRs expressed in broad time windows or during the whole development (the three *P. caudatus* TLRs; the *C. gigas Cgi-TLRα1, Cgi-TLRα4, Cgi-TLRβ4, Cgi-TLRδ1, Cgi-TLRδ2*; and the *T. transversa Ttr-TLRα4, Ttr-TLRβ2, Ttr-TLRβ3,* and *Ttr-TLRγ4*) is complex, as these genes could be involved either in immunity or in development, or both. However, detection of immune processes in our analyses is not possible with the data available. Therefore, further investigations are required to gain more knowledge on functions of TLRs during development. Immune roles of the TLRs during ontogeny should not be underestimated: Many marine invertebrate embryos and larvae live in environments rich in microbial pathogens [137, 138]. Pathogens cause mortality of embryos and larvae but also provoke anomalies during development [139, 140]. Therefore, these embryos and larvae need immune defenses to fight pathogens [138]. Actually, few studies have shown that the Toll pathway is involved in immunity during ontogeny in arthropods, mollusks and amphioxus [18, 140–142], and other immune-related genes have also been found to be involved in immunity during mollusk and echinoderm development [141, 143–145]. Additionally, in planulozoans it has been shown that TLRs are involved in adult immunity [11, 22–26]. Thus, TLRs are probably also involved in immunity during ontogeny in species across the metazoan tree.

## Conclusions

Based on our data we propose a scenario in which a *TLR-Cα* and a *TLR-Cβ/γ* were present in the planulozoan common ancestor. However, the later was lost during early cnidarian evolution. *TLR-Cβ/γ* later duplicated in the nephrozoan common ancestor, giving raise to *TLR-Cβ* and *TLR-Cγ.* Duplications and losses characterize the evolution of the three TLR clades in the main metazoan groups. The TLR complement was expanded during Trochozoa evolution, while it was lost in some non-trochozoan spiralian lineages (e.g. platyhelminths, cycliophorans, micrognathozoans, gastrotrichs and some rotifers). Ecdysozoans possess a low number of Clade α and Clade β TLRs; whereas all deuterostome TLRs belong to clade β, being originated by radiations in the different lineages. Furthermore, our data shows that TLRs are expressed during ontogeny in two ecdysozoan and two spiralian species, suggesting that some of these genes could be likely involved in development.

## Materials and methods

### Genomic and transcriptomic surveys

We surveyed TLRs 20 genomes and 25 transcriptomes (Additional file 4: Table S2). Overall, only high-quality transcriptomes (Complete BUSCO gene values > 70%—Additional file 4: Table S2) were selected, but lower quality transcriptomes were also included when they represented a species from a low investigated clade (e.g. the loriciferan *A. elegans* transcriptome (Complete BUSCO gene value 36.2%)). In order to search for the TLR sequences, hmmer profiles for the TIR and the LRR domains were generated using HMMER software version 3.2.1 [146] (www.hmmer.org). The hmmer profile for the TIR domain was compared to each genome/transcriptome using the hmmersearch function of HMMER in order to obtain a database of proteins containing the TIR domain. Next, the LRR hmmer profile was also compared to the TIR domain-containing sequences database by using hmmersearch. These sequences were validated by BLAST [147] (www.blast.ncbi.nlm.nih.gov) and SMART [148, 149] (http://smart.embl.de/). Sequences from the same species with > 90% similarity were considered to be polymorphisms or isoforms and only one of them was considered for the analyses.

### Phylogenetic analysis

The phylogenetic analysis was performed including TLRs obtained from the genome/transcriptome surveys, from NCBI database and from the literature. Since the MyD88 protein contains a TIR domain, this protein was selected as an outgroup. All sequences included in the phylogenetic analyses are found in Additional file 1: Table S1. The TLR and MyD88 sequences were aligned using MAFFT software version 7 applying the L-INS-I algorithm [150]. The alignment was trimmed manually in order to obtain a fragment containing one LRR domain, the transmembrane domain, and the TIR domain. This was followed by a second trimming step performed with TrimAl software version 1.2 using the gappyout trimming model [151]. The final alignment used to perform the phylogenetic analysis contains 375 amino acids. The maximum likelihood phylogenetic analysis was performed using IQ-TREE software [152] in the CIPRES Science Gateway V.3.3 [153] (http://www.phylo.org). LG + R8 was selected as the best-fit model (according to BIC (Bayesian Information Criterion) [154]) and was applied for the phylogenetic reconstruction. Bootstrap values were calculated running 1000 replicates using ultrafast bootstrap.

### TLR classification

TLR sequences from the genomic/transcriptomic surveys, as well as the ones obtained from the literature and NCBI database, were classified into P-type/mcc and V-type/scc. In order to do so, the number of LRR domains was analyzed with LRRfinder software [155] (http://www.lrrfinder.com). Next, sequences were classified applying the same criteria followed by Brennan and Gilmore [13]. Some TLR sequences were incomplete and they could not be classified into P-type/mcc or V-type/scc.

### Stage specific transcriptome analyses

In order to assess the expression of TLR genes, we examined publicly available stage-transcriptomic data of various developmental stages for the spiralians *C. gigas* and *T. transversa* and the ecdysozoans *P. caudatus* and *H. exemplaris*. For *C. gigas*, we examined 19 developmental time-points from early morula to D-shaped larvae, being the transcriptomic data previously published in [104] (accession numbers: SRR334225-SRR334243). For *T. transversa*, 14 stages from oocyte to 2-day juvenile were analyzed, being this dataset available from [123]. For *P. caudatus*, only 5 embryonic stages (from zigot to late introvertula) were analyzed. The transcriptomic data was obtained from [121]. The 20 *H. exemplaris* embryonic transcriptomes analyzed (from zigot to differentiation) were obtained from [122] (accession numbers: SRR1755597, SRR1755601, SRR1755603, SRR1755606, SRR1755610, SRR1755612, SRR1755621, SRR1755623, SRR1755627, SRR1755631, SRR1755637, SRR1755644, SRR1755647, SRR1755650, SRR1755656, SRR1755662, SRR1755666, SRR1755706, SRR1755715, SRR1755719). We first performed quality-trimming on downloaded RNA-seq raw reads using Trimmomatic v.0.38 [156], removing low quality or N bases (parameter settings: LEADING:20 TRAILING:20 SLIDINGWINDOW:4:20). To estimate the transcript abundancies, quality-trimmed reads were aligned to reference transcriptome assemblies (*C. gigas* [104], *T. transversa* and *P. caudatus* [100], *H. exemplaris* [112]). We applied two quantification methods: an alignment-based method using Bowtie2 [157] and RSEM [124], and the ultra-fast alignment-free method kallisto [125]. Both methods reported normalized expression values in transcripts per million (TPM), and we further executed cross-sample normalization among different developmental-stage samples by TMM method [158]. To define a criterion for gene expression value in this study, we performed in situ hybridization of selected TLR genes at different developmental stages in *Terebratalia*, as well as examining expression values in our analysis corresponding to in situ hybridization data of *Hox* genes in *Terebratalia* [123] and *Wnt* genes in *Priapulus* [121]. We considered expression for values ≥ 0.15.

### Animal collection and embryonic cultures

Adult *T. transversa* specimens were collected in Friday Harbor, USA. The eggs were fertilized, and animals were fixed for WMISH at different developmental stages with 4% paraformaldehyde for 1 h at room temperature, as described elsewhere [123, 159]. Next, the samples were repeatedly washed in Ptw and stored in 100% methanol.

### Gene cloning, probe synthesis, in situ hybridization and imaging

Specific primers for *T. transversa* TLRs were designed using the MacVector 10.6.0 software [160]. TLRs were amplified and inserted into pGEM-T Easy vectors (Promega, USA) and transformed in competent *E. coli* cells. Minipreps were prepared using NucleoSpin®Plasmid kit (Macherey–Nagel) and sequenced in the Sequencing facility of the University of Bergen. RNA probes were transcribed using digoxigenin-11-UTP (Roche, USA) with the MEGAscript™ kit (Invitrogen, Thermo Fisher). Whole mount in situ hybridization (WMISH) was performed as described in [123, 161]. Probes were hybridized at a concentration of 1 ng/μl at 67 °C during 72 h. Next, they were detected with anti-digoxigenin-AP antibody [1:5000] (Roche) and developed using NBT/BCIP (Roche). Samples were washed twice in 100% ethanol and re-hydrated in descending ethanol steps (75%, 50% and 25% ethanol in PBS). Samples were mounted in 70% glycerol. Samples were imaged using Axiocam HRc camera connected to an Axioscope Ax10 (Zeiss, Oberkochen, Germany). Images were analyzed using Fiji and Adobe Photoshop CS6.

### RNAse treatment

Specimens stored in methanol were rehydrated in ascending concentration Ptw washes and incubated in 1 mg/ml of RNAse A (Purelink RNAse A, Invitrogen, #12091-021) in Ptw for 1 h at 37 °C. Next, the specimens were then repeatedly washed in PTw, mqH20, and in MeOH; and stored at −20 °C. WMISH was performed as described above.

### Illustrations

Figure plates and illustrations were made with Adobe Illustrator CS6.

## Supplementary Information

**Additional file 1: Table S1.** Sequences included in the phylogenetic analysis.

**Additional file 2: Fig. S1.** Phylogenetic analysis alignment. Regions rich in gaps located in the positions 150–220 for TLRs not belonging to the three main clades are marked in magenta. In cyan, we mark the positions 349–354 characteristic from clades β and γ; and the gaps corresponding for these positions for the TLRs belonging to clade α.

**Additional file 3: Fig. S2**. Second phylogenetic analysis, excluding the 150–200 aminoacid region. Parameters applied for the construction of this phylogenetic tree are the same than the ones applied for the main phylogenetic analysis (Fig. 4A). Bootstrap values are indicated next to the main nodes and all nodes with bootstrap values >60 are marked with full black dots.

**Additional file 4: Table S2. **Species included in our study.

**Additional file 5: Fig. S3.** Third phylogenetic analysis, excluding the 349–354 aminoacid region. Parameters applied for the construction of this phylogenetic tree are the same than the ones applied for the main phylogenetic analysis (Fig. 4A). Recovered clades are named α, β and γ. Comparison with the main phylogenetic analysis is represented with blue and magenta dots. Bootstrap values are indicated next to the main nodes and all nodes with bootstrap values >60 are marked with full black dots.

**Additional file 6: Table S3. ***Hypsibius exemplaris *stage specific transcriptome analyses (RSEM and Kallisto methods).

**Additional file 7: Table S4.** *Priapulus caudatus* stage specific transcriptome analyses. Analyses for the different methods (RSEM and Kallisto) and replicates (Rep 1_and Rep_2). For each method, average and standard error (SE) of the two replicates is provided.

**Additional file 8: Table S5. ***Crassostrea gigas *stage specific transcriptome analyses (RSEM and Kallisto methods).

**Additional file 9: Table S6.** *Terebratalia transversa *stage specific transcriptome analyses. Analyses for the different methods (RSEM and Kallisto) and replicates (Rep 1_and Rep_2). For each method, average and standard error (SE) of the two replicates is provided.

**Additional file 10: Fig. S4. **RNAse treatment experiment followed by *in situ *hybridization for the *Ttr-TLRα4* gene. A. RNAse treated animals only show the ring-shaped staining (white asterisk). B. Control animals show expression of the *Ttr-TLRα4 *gene in the brain and the pedicle (blue arrows) and the ring-shaped staining (white asterisk). Therefore, as the ring-shaped staining is present in RNAse treated specimens, we conclude that it is nonspecific staining.

**Additional file 11: Fig. S5.** Different scenarios for TLR evolution. Our phylogenetic analysis suggests different hypothesis about when the duplications that originated the ancestral genes for clades α, β and γ occurred. First, we can hypothesize that in the planulozoan common ancestor there were present either one (*TLR-Cα/β/γ*)—hypothesis 1A – (panels A, C and E) or two TLRs – hypothesis 1B—(panels B, D and F). Second, the duplication of *TLR-Cβ/γ *that gave raise to *TLR-Cβ *and *TLR-Cγ *could have occurred either in the nephrozoan common ancestor—hypothesis 2A–(panels A and B), the spiralian common ancestor – hypothesis 2B—(panels C and D) or the trochozoan common ancestor—hypothesis 2C—(panels E and F). The panels in this figure show the different combinations of these hypotheses.

## Data Availability

The datasets supporting the conclusions of this article are included within the article and its additional files.TLR and MyD88 sequences obtained  in the genomic/transcriptomic surveys and used in the phylogenetic analyses are available in Additional file 1: Table S1. *Terebratalia transversa* TLR sequences for which in situ hybridization were performed, have been diposited in NCBI database with the following accession numbers: *Ttr-TLRα2*: OL353900,* Ttr-TLRα3*: OL353901,* Ttr-TLRα4*: OL353902,* Ttr-TLRα5*: OL353903,* Ttr-TLRβ3*: OL353904,* Ttr-TLRγ4*: OL353905,* Ttr-TLRδ*: OL353906].

## Abbreviations

AMPs: Antimicrobial peptides
BCIP: 5-Bromo-4-chloro-3-indolyl phosphate
BIC: Bayesian Information Criterion
BUSCO: Benchmarking Universal Single-Copy Orthologs
IL-1: Interleukin-I receptor
LRR: Leucine-rich repeat
LRRCT: Leucine-rich repeat C-terminal domain
LRRNT: Leucine-rich repeat N-terminal domain
Mcc: Multiple cysteine cluster
NBT: Nitro blue tetrazolium
NOD: Nucleotide oligomerization domain
PBS: Phosphate-Buffered Saline
P-type: Protostome type
PTw: PBS with 0.1% Tween® 20
RSEM: RNA-Seq by Expectation Maximization
Scc: Single cysteine cluster
TIR: Toll/IL-1 receptor
TLR: Toll-like receptor
*TLR-Cα*: Clade α type TLR gene
*TLR-Cβ*: Clade β type TLR gene
*TLR-Cγ*: Clade γ type TLR gene
*TLR-Cβ/γ*: Proto-TLR gene of clades β and γ
TLR-like: Toll-like receptor-like
TM: Transmembrane
TMM: Trimmed mean of M values
TPM: Transcripts per million
V-type: Vertebrate type;
WMISH: Whole mount in situ hybridization
