# Supplementary material for: The evolution of the metazoan Toll receptor family and its expression during protostome development
Source: BMC Ecol Evol. 2021 Nov 22;21:208. doi: 10.1186/s12862-021-01927-1 (PMC8609888; doi:10.1186/s12862-021-01927-1)
Supplement: Supplementary file 2 — Additional file 2: Fig. S1. Phylogenetic analysis alignment. Regions rich in gaps located in the positions 150–220 for TLRs not belonging to the three main clades are marked in magenta. In cyan, we mark the positions 349–354 characteristic from clades β and γ; and the gaps corresponding for these positions for the TLRs belonging to clade α. [file 12862_2021_1927_MOESM2_ESM.pdf]

Additional file 2: Fig. S1. Phylogenetic analysis alignment. Regions rich in gaps located in the positions 150-220 for TLRs not belonging to the three main clades are marked in magenta. In cyan, we mark the positions 349-354 characteristic from clades β and γ; and the gaps corresponding for these positions for the TLRs belonging to clade α.

>Mme-TLRβ3
NYQTIKNMVYLDLSDNKISIIP----NDIFYQMVKMSHLNLIGNQIVSLDNNMFLYNANL
QSLYLSNNRLTTFDSKLLNHCKALERLMISQNKISLFDVEFVNEIDSLRSVRIDENPFDC
SCGQLFFQSWVQRSKK----KFGQDLQCVQPSNLLNQKISEY---QPQQCV-KWVVDGVA
----LVGLVLIIIVTYQLRWYLTNIPYTRSMRY------RQQDECHYDAMVIFSNKDTDW
V-KELMRELEEDRENKIYIRVRDDISGTITFEECSKVMNQSRKIILILSNSFLAETECIS
EVEFAGNELFSTANGRVMILVLEELQESRIDL-VRSLMIEANVIELLESTRKMREVWQKL
KKFVNHQKP
>Mme-TLRβ4
NFKTIKHLTYLDLSDNSLPSIP----NDLFDNMPKLKTLKMISNLISSLDRRQFIHNSQL
RTLDMTKNLLETFHVSVFANDTLIKALSLRTNKISMFDETFTQFVSTLTYLRIENNSFDC
SCGQLYFQKWANSSKK----QYGDKLICHSPGQLRNQRIVIY---QLFDCY----WVLVG
LA--LVGIITTVLLMYRFRWYLAHLRFALSVAE-RLVDIKQQDQCKYDAMVLFSDEETNW
V-KRLLIELEEDRENLIYIRARDDITGDVNFDSCCKIMKQSRKIILVLSNSFLREDECIS
EATFAGSELFSTAKERILILVLEELQEPL-NP-VSSLLVETHYIDLYETTRKMRAIWQKL
RRFVSHRAN
>Mme-TLRα
-LTAFPNIKI------------------------AMEELSLSHNTITELPEDPFYWLRHV
VHLNIQHNELTPQNIPIFDRLGKISYLFLAYNNIVYFPPSIR---KNFKALSITGNKITC
-CNGHWMKTWLQEQNE--TIWNSLTAHCKD----QAHPIIQLDPDGFS-CP-LYLAPIII
SIALTIATMMTSVAVYVYSFELKIILFKLNLHPR---SV-DSESLDYDLYLMYNYADSPW
ATEKLLPGLE-
KFGYRVYVPERDMGIGEITAEARANAFASTHRVLVVVSQKFIDSGESMK
EFFHAHEHENSTTRRYLVLVKLEKI--NRTD-IFKKYMSTNFFVSVKS------KFWYNL
RYWLPREST
>Mme-TLRβ5
LFHGMKNLKTLLLMHNHLGLSFSDDYTGFLSRLPRLMVVNMSSNGITVLPKQMISNTS
AL
EVLDLGMNHIYSWDSQTFQGAVGLKKLLLNTNRLALFNETSFSDLKNLTVLDLGNNPF
AC
TCDMRWFRDWLKTT--KVHVNNSHTYTCTSPAKMQGTHLIEFELTTLQ-
CVPVWIVSGCF
IS--LLLILIMVCVLYRYRWRIRFALYKCSKSCKAYQRLPQTDRPLYAAFFSFCSEDENI
IEEQILPNIDNDAGVYPLIHRIKYDPSRTYLDCLEKALLTSPATVVMLCQHYKEDRQCEL
ELAAS----LQEEDRRIILVVLDDIVQRKLPVALRVMLNRNEAIEWHRNEQQARMLKGKL
AEALED---
>Mme-TLRβ2
NFKAISNLEYLDLDTNNLTFIR----NHTFDHMPNLNTLILSANNLKHIDDQAFIHNYNL
ATLLLQANKFSVFNVTLLEGPKNLRKLCISTNLITHFDSSFVKFMGTLQTVKIANNPFDC
SCGRKFFSDWLNRTKL----HESVGLECTTPENMAQKKVYNY---EDLECT-PLIWSAVV
LC-LIIVTIMIAVPCYRYRWYISHMVIMQAVKD-RAMDIKHSDECKYDAMILSSEADMKF
V-KTLLLHLEEDRSNRLYHSLRDAIPGTYRFESLCEVMRQSRKIIIVISNSYLNSSECMS
EAAFAGEELFGTKKEKIVVLVLEDLNEDLMPS-IAGLLTET-VIDLPESKKTMAPVWDRL

KKFVENKP-
>Mme-TLRβ1
FYPQLKQLQKLDISGNSFKYLD----PNAFNEMKYLSSVIAQSNPLSILPDTIFISNMKL
VKVDFSNCFLDELDNTIVKSLPKLEHLYLKYNQFTSFAPSVISVVKALKTLSLLGNPFDC
DCNIGRLQDWLSETEK----IDVINITCGGPEHAADTSIFEY---PPPRCK----LPLII
GC-AVVGFVLLILLCICSRWYISHRKILPELKG-ILKNIRYGYKCDYDAVVCYSDIDQQW
VGGRLVPALESKKA-RLYIYERDSTIGAEKTVQIRDAMERSRNVIIVLSKSYLASEAFLP
EVDIVADVMRQNEKGRILLLALDDLDNRKMDP-IKLLTLTEKTLNVV------------

>Goc-TLRγ23
SLSCWPRMRKLLLGNIELQQIFDHGNETIFNNCTYMKTIDLQNTGIKRLPQNTFLDMEN
V
QYINISNNKLTSLDI--LTSTKNL-TLNISSNLLDHLSNKMTQLLDQMEILDLSYNPIMC
GCPQIDFIVWLKNT--YVEIYNLNQYQCVYQDGTK--YINDISLSQLKQCN-IIIQAVCS
IGS-VVVIGIISIIIYSYRHKLEYLLLIARHAAKSKKDKHNDKTFNFHGFVSYSSEDDLW
IVEQLHMKMEQDFGLKLCIHERDFLPGYFITENISSFMEASRKTVIVLSNNYLKSKWCTF
EFELAKCKLIEATFNTMVMILLHDLDQRKVSPALHKYLKQKTYLKWPKDSSQQPAFWL
RL
KEALDQKPE
>Goc-TLRγ17
VLNCFPSLTEVRIGGNQL----NT-HIMMFANCTKLTHLDVSHNKLVSMPKDAFHETPNL
IHLNLSGNLFANLEIHEVTQLTKLQVLDLSYNRFQAIPESWRHTIQLLGKLYISGNPFMC
SCDTVDHLIWLQSI--QDLLDDPTHLTCRDTNGKEY-TIMQIHIGRFKECIKSMVQAGCI
PSAIVLVIVGISLYIYKRRFRFQYLALVARANIN--LHAPQIPDYTYDAFISYSSLDIEY
ML-TLYQKLEQEHNYELCIDMRNFRPGNPIDDEITNGIMDSHKIILVISQNFLRSGWCLY
EMQLAHGELAVRGGDGLLLILKEPRPQELITDKLQGLLDSRIYLEWSEEGDRQQVFWQ
RL
RDALGMPLQ
>Goc-TLRβ11
VLSNSTMIESVDLSGNLLFRYTDEFLCDLLKNLVNLEEISLFDNYLTHVPSCLFRASSRI
IGIYLSRNRIAYIQKGVFDSLYQLEELDLDDNSITFIDPSNFYNTPSLSWLTIENNRFSC
DCRLTGFRDWTAEH--QDIIEGP----CETPKQLKGEAVHAYTTTWLE-CNTVFIICGSL
----LFFLLVVTGLLFYFWKDIKYIKMVHRAKGKGYIPLNDNNQVLYDAFISYHPEKKFW
VEVDLIPTLEDDVQFNIMYDER-FDTG-SIFTLTEENIAQSRKILFVVSRGWIQAGWNQF
ELDMAMIKLIDDHRDMIIVLLMEHIPKKEMPDKLKMMVKYNKCLKWSDNEHKQRIFRRD
L
KLELGKE--
>Goc-TLRγ16
VLNCFPSLTEVRIGGNQL----NT-HIMMFANCTKLTHLDVSHNKLVSMPKDAFHETPNL
IHLNLSGNLFANLEIHEVTQLTKLQVLDLSYNRFHVIPESWRHTEKILGKLYISGNPFMC
SCDTVDHLIWLQSI--QDLLDDPTHLTCRDTNGKEY-TIMQIHIGRFKECIKSMVQAGCI
PSAIVLVIVGISLYIYKRRFRFQYLALVARANIN--LHAPQIPDYTYDAFISYSSLDIEY
ML-TLYQKLEQEHNYELCIDMRNFRPGNPIDDEITNGIMDSHKIILVISQNFLRSGWCLY
EMQLAHGELAVRGGEGLLLILKEPRPPELITDKLQGLLDSRIYLEWSEDGDKQQVFWQ
KL
RDALGMPLQ

>Goc-TLRα2
-LHNLATYP-LNVSYNNLNKIEHCLIPYLFDSLYALESLDLSFNLLTSVPSQLFSSLISL
SALYLDHNDIRFLPQGMLFNSTHVGKLTLHQNKIETLQINIFQSLQFLTTITLADNPWVC
NCSMFDFCKWLHSNWT--KVEDKSSLTCKNGSN-----LIQFT--YNCTCKQDNRIVLGI
CGSLITLLSIGLGLVYYYHDNLRFFLYLFGWRF----PVRNNGEAYFDIFICYSSKDNKY
VITKLLRYLETKPPYKVCIHERDFIPGDYIIDNIVRCINKSKTIILVLSNNFVNSMWCLG
EFQMAYHNAFENRHNNIIPILLGDLNLDHLDPTLRTFVGMNNYLRKDE------LFLQRL
LVALPEPSN
>Goc-TLRγ10
ALTCMESLEKLNIANNNF----NETDINIFENCTKLHILNLSYNELENIPKDTFNETTNL
ANLDLSGNKLSNIAF--LENQRNLTFLNLAGNSIQYISPPLTQDISRLMKINLDDNFLRC
GCEDIVFIDWLKNN--EDQIINWEKLKCIDDAGLY--NIQTINTEWTQQCNMNIIIAMSI
MTGFILFTTIIACCLYRHRYKVHYLYLLFRSWFH---KPDDANQYNFDGFISYSSLDKTW
ALETMYANLATKYGYNICVDERNFRPGQHLVDIIIETINTSNKIMLVITQNFLRSGWCLY
EMKMARGELATRGRDCLILILKDPIPQELITPTLRQLLESRIYLEWSEDRDRKALFWRKL
CDALGEPRH
>Goc-TLRγ9
ALTCMESLEKLNIANNNF----NETEINIFENCSKLHTLDLSYNELENIPKDTFNETKKL
VNLNLSGNKLSNIAF--LENQRNLTFLNLAGNSIQYISPLLTQDISRLMKINLDDNFLRC
GCEDIVFIDWLKNN--EDQIINWEKLKCIDDAGLY--NIQTINTEWTRQCNMNIIIAMSI
MTGFILFTTIIACCLYRHRYKVHYLYLLFRSWFH---KPDDANQYNFDGFISYSSLDKTW
ALETMYANLATKYGYNICVDERNFRPGQHLVDIIIETINTSNKIMLVISQNFLRSGWCLY
EMKMARGELATRGRDCLILILKDPIPQELITPTLRQLLESRIYLEWSEDRDRKALFWRKL
CDALGEPRH
>Goc-TLRγ3
SLDYFPSLKILLLGSNGLGPLIHDNDGRLFANLSSVVSLDIADNSIQTISPNACSNMSNL
QFLNLSQNEMFTFHL--ISHIRSLKLLNLSNNRIHLFSQDTMDMFDDLATVDLTGNLFTC
GCSDL-YIIWLTDTMIRSRWLLYEHYTCLFNNG--TITLSQVDVSQLWDCHKPYIVMASM
II-
FALLVAVLVKLLHYHRWTLQYWYFMFKRAYRRQQELEQNLVKTYDAFVSYHTNSAQW
VYEHLLP-LERDENLKLCIHQRDWIPGQFISEIIVESVKQSRKTLMVVTKEFAESKYCLY
EMQMARNVLFDEGVDALVVVLIDEILSRRINSTLRYLIQRKNYIQWPDEN-----FVPKM
KAALARE--
>Goc-TLRγ1
VMHYFPSLKYLNMANTNLKMHLKDTNGSFFSKLSNLQTLDISKNKMETFSKKTFCHLT
KL
KHINLKSNNFVAFEL--MNSV-HFLQMDLSDNKISHLTKSNLRLFELMTTINLSDNAFMC
ACEEQEFLKWLKERRIVDIMQHYSKYDCLVTQTQNKVAIRSINMDEFNQCD-
AILMAKVV
AGLVAILVGVLTKVIHYNRFTIRYWKFGIAWMWRRRREEQDQVEYKYDAFVCFQNDDI
DW
IYNELRPNIEQEGAFKLCIHHRDFSPGEFIIDNIVNAIEGSRYAILIISKNFLKSGFTKL
EMQLAMKVMIMRQAEMIIPVMLDDVQHPDMYRALKYHIEKKTCITTNE------HFWEKL
RAALKRE--
>Goc-TLRγ5
VFDCLPKLKYLNLANNKF----TNIELELFSNCSNLTYLDVSYNQLKTLPENLVAQTANL

QHLNLGGNQLRQFDV--
LVPVKTLSTLNVSNNLLGYLKDEMCLQLSKMHRLDLGNNPWLC
TCDNVEFLRWVQQS--TNMLLKPDELICNDPHGNFV-LMANINISKLSGCYTTTIITVSA
TIVITLLIILLAVLAYRRRYKIQYIYLIIRAKMRGFR---QDRQYTFDGFMSYSSLDCNW
VTGVLHKTLEDELDYKICIDQRNFMPGSYIAEAIAEGINESKKVILVITQNFLRSGWCTY
EYNMARGELANRGRDCILLIMKDPIPKEHITQTLQTMLESKIYLEWSEDDDKKQLFWRK
L
QDAIGEPQG
>Goc-TLRβ10
VMSNCRCLKIVNLKENLLFSY-EKELCRLFKWGKSLQKISIPSNYLAVLPKCIFRGLSQL
TSLYLEKNRLHVIGKGLFTDLINLEFLDLSFNAITYMDSGNFLAMTRLKSLNLNRNNFHC
TCQLLPFRNWIREK--VNLKNFRYNDTQCSLLDRKHVFVHNYTISWLE-CNKVFVVSASS
IGGIFIVVALIVTLLYNYWRDIKYYRMVHKARRHDNRQE--IAEIEFDAYVSYHPEKELW
LRVDLINNLEDDISFKVTFDDR-LEPGRSVIGSMAEAIHKSRKILFVVSRGWLRAITTQL
EIDMALVKMIDDHRDMIIVLLMEHIPKDEMPDKLKMMVKHNTCLKWSDNEKQQAKFWR
DL
KLELGKH--
>Goc-TLRγ20
LLHCRQNLTVLKFAQNDLSPVF-NSKQPIFAGCNNLKELDISRNSISEIPNNAFVDLKNV
EEIDLSGNELTNVVV--LENCKQLRVLNLSSNPLTNLDDPTMTVLDKLQTIDLSRITLGC
GCNDVAFVHWAQTT--RVKLFNSDTYKCTYLDSSR--ALMKVSIFSLRACMKDVIIASTV
PTITLILIFIAGLYVYHKRWRIQYHCLLLREVARRYE---QLDELTYDAFVCYCSQDEDW
VAEILRPKLEDELNFKLCIHEREFIPGMDIQDNIVSFMQDSRNTILVLSEHFVESRWCQW
ETRMARNKLLDSPRDNLIMILLQDVLKQKMNPTLKSLIEMKTYLRFPQKAEELPVFWLR
L
KNAMSEHVK
>Goc-TLRβ9
VLNHSNAISLINLKGNILFRY-DSQMCSMFKGRANLSHIDISNNYLFRLPSCIFKGLKNL
KTLYLQDNRLTYIQHDIFIDLYKLHRLNLSNNAITLISPITFAPLANLKVLRIGHNNFQC
YCEMKELRNWLGHN--IKKL-GHHKEKCSGPLTRQDEFIHSFTVSWME-
CNGLSTFGSIG
IISIVLLSVITFTVLRHYWRDIQYIKMVRRARKHKSHPEN-NCLIEYDAFVSYHSDKQIW
VIRDLVNELENDVTFRVMFDER-
IDLGTNIFTSMEEAIDKSRKMLFVVSRGWVAAAMNKL
EVDMALVKMIDDHRDMIIVLLMEHIPTNEMPDKLKMMVKHNTCLKWSDTERSRAKFWR
DL
KLELGKH--
>Goc-TLRβ8
VLNNSHDIYKINLKGNLLFHYFDYQLCDLFQSKSNLSHIDISKNYLSRIPACMFTGLSKL
HILYLQENRLTYIHKDMFKDLHNLQRLNLSNNAITSIDASAFVPMTLLNRLWINQNNFDC
NCDMKGFRNWLGHN--KKILSGSIKEHCSHPLIRRNEYIHNYNVPWME-CNGLSTISTIT
ISLMVVLSVATLTVLKYIWRDIQYIQMVRRARKHGNYPLG-DIQTEYDAFVSYHVDKQIW
VMRDLVNELENDIQFRIMFDER-
IELGRNIFTSMEEAIDKSRKMLFVVSRGWVAAAMNQQ
EVDMALVKMIDDHRDMIIVLLMEHIPTNEMPDKLKMMVKHNTCLKWSDTERSRAKFWR
DL

KLELGKH--
>Goc-TLRα5
-FSSLPALPKLHIANTSLTNIN--------------------------------DYFAKV
TLLDVSNNSISHISQDVLQGMTVLKTLYLHGNKLQYIPEYMMNLK--LDHLSLSDNPWAC
DCKNAWIKPWLNANVS--ITIGFEGIKCHGG----GKQLLHYD--FEAMCNL--VVIGVP
VF-IVIFIILMVAIVTNYRQVLTLMIRHHIF--------EEPAGKTWDAFLGYATDDVEY
VQNVIIPLLE--PKYKLCVHNRDFQPGVPIIDNIAEAVDKSQRTIMILSPNFLQSQWCLS
EFRIAHMQYLNHPSKLLIPILLDDFSPAECTA-IKCHLQAHTYLEAKD------WFDRKL
LQQMPKVSL
>Goc-TLRβ6
LLEKSKDLIQLDLSDDMLFTYSDNELCRIFSSQSKLEILTLAGNYFSSLPVCMFQNLHHL
KDLDLRKNRIPLIQRNLFSDLRNLSVLDLRENSITFIDVTDFLKLNKLKTLYLKNNLFAC
TCDLRPFQSWVLGVSQ----KTDGPLRCSSPEQRKNNTVRNFTATWIE-CNELVMYLTIT
LSSILIITSIILLTLYNFRNDIRYRRLLQQVKRKTYTKLQDA-AIKYDAYVSYHV-EKQW
FMEEISKKLEKEIQFNL-IHDDNIVAGESIFGSMSKAINRSYNIIFVISRGWIHDPARAI
EIDEVNGILNREKRHNIILLIMEHIPPEQIPGNLKMMLRNNVVLYWNEDPKRQRIFWRDL
ILELGKTKD
>Goc-TLRγ15
FINCFPSLVELNLSGNQ-----GMIDVVMFDNCTNLRVLDISNNKLASLPYDMLHHVPYL
EKLNMAGNFFTHLDILNFMETKTLQSLNISSNHWRTFPDTWQKVISGLVELDIHGNPLV
C
TCDTIDHLIWLQNI--QVALYKPSTLTCMDLYGQEH-NIMDINMYKYRDCL-PYLMAGFI
PCTVTLVLIGLILYAYRRRYRLLYWLLELQAKLR--QPHAEERNFVYDCFISYSSNDIDW
MIEMFQKL--EQHNYKMCIDMKDFRPGSPLVDEINQGIIQSRKVILIITQSFLTSGWCNY
EMDIAHGELALRGEDCLILVLKEPRPQALITPALQRLLEERIYLEWSNDHDRQAVFWRR
V
QDALGEPLQ
>Goc-TLRγ19
FLACRPNLTIALLSQNNLSPIFDTPKQTIFHGCSKLKVIDISENQIKTIPFSTFIDLIQV
EIINISGNYLRQLDV--LELCTSLNLLNLSSNLLTTLSSRMTSSLDTLQTLDLTHNSLMC
GCNDIAFIDWAQQT--NVRLHNGHRYTCTDKDSHQ--QLLDISVYHLSECKKEILIASIV
PSVGLVIIFSIGLLIYNKRWRLQYRYLVAREMVRGYV---EIINLPYDAFVCYCSQDQTW
VAEQLRTKLEDEFNFKLCIHDRDFIPGMDIQENIVKRLEESRNTILVMSQHFVESRWCQ
W
EARLARNKLLDSPRDNLIMILLDDVLKGKMNRTLKSLLEMKTYLQYPHNEGEKQLFWM
RL
RNVLMENRR
>Goc-TLRα4
-LTQLPTIPPLLLSKNSVSRFE--------------------------------PYLANV
SVLDLSWNGLHEIVIEALNSTRDIEQLFLDNNALTELPKSIKDMPPMLQLITMHGNHFRC
ECESAWMKSWLQAQVDNGRVNSSLKIQCADTQT----EIIH-D--FHELCL-NFLVVGVP
LA-LVSLIVFAFAVLYKFREVIILTIR----KK----PTEKPEGKLYDAFIGYATEDVLW
VQDVLIPILE--PQYKLCVHNRDFVPGTPILDNISEGIEKSQRSIMVLSPKFLDSHWCLE
EFLQAHRQYMAHSSQILIPILLDDFEPDNIVAYIRCYLQSHTYLQHAD------LFARKL
RIHMPKLTV
>Goc-TLRγ4

IFNCFTALRYLDIANNQL----KSKDSVFFANCSHVEHVDLSSCTIGEVPRHLLAQLPNI
TYFSLSGNRLRKLDI--LNK--KLNLLNVSSNLLSAISPGMLSQLTHLHTLDMSSNPLQC
MCDTTTFMTWVQQS--TELLHNPSDLLCMTSAGDMV-AIVHVDVAAIHQCILPTILATTL
TTTGILGIIISLLIYRKRYRIQYIYLIIRSKLS---ES-KQARFPFDAFISYSSLDSRW
VVNTLYSTLADTHAYNVCIDQRNFMPGAYIADAIVEGINDSNKVILVISQNFLRSGWCVF
EMNIANGELANRGRDCLILIIKDPIPQELITKTLQALLESKVYLEWSEDPDRQRVFWLKL
MNAIGPKRD
>Goc-TLRγ6
----FPKLKTLQLRNNKV------SNVEMFANCTALAHIDLSHNGIYSLPLQLFRHTPNV
NYVNLAGNKLHTLA-FEVVFLTNLALLNLSNNNIQILTRNFQENVDQMSFIDLDNNALIC
TCDNVDFVRWIQGS--WHFLLQSNQIECKDGGGVS-HSIIDIDVNHFHGCIRSTLIASLV
PNVFFILCILLGVLFYRKRHKLHYLYLLARAQMRQRRNV-
DRGNYCFDGFISYSSLDTDW
VIEHVYNELADHHGYNICIDVRNFMPGEFIADVIIESINQSYKVILVISENFLRSGWCTY
ELNMARGELSIRGRDCLVLIFKQPIPRELITPTLRSLMETRVYLEWCHEADKQQVFWRK
L
LDALGQPRQ
>Goc-TLRβ7

----MKENRLTYIHKDMFKDLHNLQRLNLSNNAITSIDASAFVPMTLLNRLWINQNNFDC
NCDMKGFRNWLGHN--KKILSGSIKEHCSHPLIRRNEYIHNYNVPWME-CNGLSTISTIT
ISLMVVLSVATLTVLKYIWRDIQYIQMVRRARKHGNYPLG-DIQTEYDAFVSYHVDKQIW
VMRDLVNELENDIQFRIMFDER-
IELGRNIFTSMEEAIDKSRKMLFVVSRGWVAAAMNKL
EVDMALVKMIDDHRDMIIVLLMEHIPTNEMPDKLKMMVKHNTCLKWSDTERSRAKFWR
DLKLELGKH--
>Goc-TLRβ4
FFTDFPSLKMLDMSNNDFSFL-HQVLAKMILGFENLEILRLSNCQLSDVP-NFFDGGEKV
TELDLSWNLIGHFRNGVLNKLRSLRKLYLQHNHITHIDPLNFERMNDLQYLDMTNNRFT
C
DCEQREFINFVKGNQ-HRIIFHGRRTKCH-PDSAYNTFLLHYTSPWIE-CDGNFIMILSF
TIILVVIITCIFIFLYNYT-SIRYRSALCKIKCNQYKSL--GHQYDFDAYVIHHPTDISW
ILYELIPHVEQHFSFELCIEERNFLPGPFKTDNLARAILRSRRALLIISKDFLESDWFRL
ELEMAQLQHLNGREKYIIIIFLDEIPASQLPMKLKCLMGFTTSFVWPKNRSKRNEFWRG
LLLELNKKPP
>Goc-TLRγ22
FFDHFPKLKTLLLGNNELGLLFASDDFLFFRNSTTLESIDLAGNNLSRIPPLLFKDTKNL
QYLNISNNYLDSFNI--LSTLSHLKYILLSNNKIRVLSSTTRHQINTLAHVDLSGNPLLC
DCNNLDFLHWLRDA--PLVFDNKDSYQCTGMNNFR--KVYDINVKDFEQCKINMIKTIAS
TAGTALITMAVVIAVYRKRYRLEYLWLVSKATVKKREGNDENGRYIYHGFVSYSGRDD
LW
ICDQLHIHMEQVMGLSLCLHDRDFIPGEFITDNIIKSMEASRKTIIILSNNFLESRWCEF
ELQMAESRQAEMTYNTVITILLHDVNQNKIGPLLKKYLKQKTYLAWPRDHHQRPAFWL
RLKDAIDREPD
>Goc-TLRβ5
LFRS-SNLSEVSFSGTYVGHSDVTLLKDIFHGHHNLQTLRLDTTHIQELKSGTFWSLTRL

EVLILTSNHLKTLPTDIFQGLVSLQYLDLEDNDFIHLDPNMFTQLPNLKLLWISSNSYHC
GCDLRPYREWLNHS--KVVL---PPGRCYSPSKLNNQIVDEFSLPWLE-CDDTLLISGTL
AG--
LVIILSHGYAVWRFRFDMKYWYYITRAKRAAEQPLQDGGNIQWDAYVTCTPQDQKF
IYDHVIPNLEEDFKFKLCYGPRDFLGGSEI-GNRENALNNSHRAIFVISKEFMKNNWGKF
ELEMTQLKLFDDHKYMTILFFMETIPKSEMPELLKLLKRHSLCLYWKSENREQNVLWK
RLKLDLFKARQ
>Goc-TLRγ14
VVNCFPSLKYLYLSGNNV----QGHSPRMFENCSKLKVLDISQNKLQVIPFHTFNETPNL
EEIHLSGNYFSDLEILDFKDTKSLRLLNLSHNQFHALPEFWKDTIAEFKHLDIHGNPLVC
SCDTVDHLLWLQSI--RPLLYDADNLTCKNNQGRQ--FIMEINISEFKECIKPLLLAGCI
PSAIIVITLTLALCIYRRRYRLHYLTLILRARLRKYINSEQRQDFLYDSFISYSSLDVTW
MVDILYKNLSERLNYELCIDVQNFRPGEAIVDEILAGVLESKKIILVISQNFLRSGWCNY
ELKIANGELALRGEECLILILKEPLPKELITPTLRRLLKSRIYIEWNDIEDRQQLFWRRLQD
AIGEPMA
>Goc-TLRβ3
SFKGLLSLEKLSLARNHLDNS-DGTIVTLLKSLPRIKELDLSWNHLTYIPKSSLDSMENL
TKLDVSGNRLTSFTVDKVRTNTKLNQLNVSRNALASIEALEIGKVTLLNNLDIRYNKFKC
GCELRYLRNWLLEKNHKFSL---YSEKCFAPIEMINTSVMDFKINWIY-CDHLIISLSSA
GG-FLAIILFCIVLSFVFYWDIKYWWALRRKGLGGYLPLDEESKLSYDAFVSYHTSSESW
VADKMVKNLEDDVNFKLCLHGRDFLPGRYIADNIIVTMRNSAKIIFIITQKFLESQWCGY
ELEQAHIRQFDEEKHLVILIFLEKIPKAKLPKKIRLLMRHVTYLEWDKTSRAQNLFWKKLK
LCLLDKPT
>Goc-TLRβ2
SFKGLANLEKLSLARNYIGSS-DDIIVMLLHLFPKVKDLDLSSNHLTWIPKTALDAMKDL
TILDFSVNRLTSFPLENVNHNTKLERLNVSRNALVNIEAPQIKADTKLNNLDIRYNKFFC
GCDLRPVRDWLLAQRFKVSI---FSERCSAPTELRGTPILYYKINWIN-CDSLIISLSST
GG-
FLVIVILCIVTVCIFYWDIKYWWALRKRGVRGYIPLDQQVQLSYDAFVSYQTSSQEW
VAEYMTKHLEDDVNFKLCFHGRDFLPGRYIADNIIVSMRNSAKIIFVITQQFLESQWCGY
ELEQAHIRQFDEEKHLVILIFLEKVPKSKLPKKIRLLMRHVTYLEWDNSSRAQNLFWKKL
KLSLLDKPT
>Goc-TLRβ1
SFQGLSSLKDLNLARNNLDFSVQSLLSKMFSALKTLKKLNVAFNHFMSLPGDVFDGLQ
SL
EYLDLSMNKLSFFGKRYIRNVKSLRVLNLRRNMIIKFDMNILKPPYKLVLLNISENKFIC
SCDLRGFRDWLDSKPKHITI---FDQNCSDPDTMKTSRINDFTIPWID-CDNLTISFVSA
GG-FLIIFSVTVVVLVHFRWELKYWWFLRLSRRRNYIQL-
HDDGFQYDAFVSYHEESSRW
VYDYMPKELEDDMSFQLCFHGRDFIPGQSIQTNIANSISQSRKIIFVITQGFLDSNWCTY
ELEMANIHQFDKKKNLIILIFLENIPKYKLPKKVKLLMKNVTYAEWEENNRSQRIFWKRM
KMALMDQPT
>Goc-TLRγ12
FFDCFEKLRVLNIANNDI----VNLTFTVFTGCNRLEYFDASYNNLKDIPTSAFQQVLNI
KQLVLSGNHLRDFDA--LQGLNNLQTLNLSDNILTNLPLQVRNSLDDIASIDLYGNRLSC
SCETIYFIEWLHNF--KENVYKYEGLECSYIDGVF--PVASVSIFRLWHCWAPLIMSIVS

----TIAAILFIFAIYKSRYKIQYRYLIVKGKFKRYQAAPSHDNINFDAFVSYSDHDIIW
SVETLYCKLAHEWRHNVCIEGRSYRPGGFRNEVVMEGINESNHILLVISQSFLKSGWC
AF
ETRIAHGELVHRGKSCVMLILKEPKPESLIGTVLRSLLDNGCYIEWSNNPDKQRLFWYK
LQDFLGEPIN
>Goc-TLRγ2
AFCFLSNLKMLVLNDCKLNDILTDTETSLFKNLLMLESLQLRYNNLTYFNVSHLNSLSHL
KHLDLSQNKLSKFDFAQVSK-PNFTNLDLSSNRFRMFSSDSMKSLDAIKTINLHDNILTC
ACNMLKFMLWMHNN--
KGHMVQYSTYQCVFSHNDTEMNLIDIDMVSFSECHKDYIIFSVI
SVIITLLCCLIGLILYKKRWTLRYWYFILRQSWRRRRDA-DVMHYHFDAFIVFHSDDYEW
ILNELLKQSEEPHGLKYCIHLRDWRPGNFVSENIVQSVERSRHTVLIVSKNFTKSKFCY
Y
EMNVARSLLTSHGRDVIIAILIDEIRKAGTTATLREILRQKTYLQWPSEN-----
FWERYHTMMDDNEE
>Goc-TLRγ8
FLTCFESVETLIIGKNYV----NGREFNVFQNCSKLHYLDLSFNGLTGIPWRAFNETPSL
IALNLSGNQISYPRF--LEAASNLTSLDLSNNKIHYMDESMRTNIGTLMSLNLDNNILLC
NCNGITFIEWLQKY--KQNIVNWDKLKCIDSKGTEK-KLIDINITLKKECLMDIIIASST
VSGIFLLVVFIAVCTYRRRYKLQYLTLLLRAWCR---KPDDGDQYNFDCFISYSSLDRIW
TLETLYTTLATKHGYNICFDERNFMPGQHLVDIINESIFTSRKIILVITQNFLRSGWCLY
EMKMARGELAARGRDCLILIMKDPVPKDLITPTLRQLLDSRIYLEWNEDVDRQQLFWR
KLRDVLGEARH
> Goc-TLRγ21
VFHCWPNVKTLLTGGNDLSHVFYEENIDYFENCTHLEVLDLANNKINKVSKNLVKTAIN
LRQLNISRNQLSMFDV--LDNNQQLELMNLSNNILGSLPISLTLTLDHINIVDLQNNPLLC
SCATLDFISWCQTT--KVHLNNLPSYTCTDGTQRQ--YLMDVSVSHVDKCKEPVVIAAVS
TSLITIIFVIIAITIYTKRWSLNYYLLLSKIFLRRRNRYSENTSYRYDAFVSYSSNDDDW
ILRNLHPILEDEHGLKLCLHQRDFIVGNDIQDNIIESIEASRKTIVVLSNNFLESKWCYF
ELQMARNKVLDGKLDLLVLVLLDDLAKDLVTATLRTLLATKTYLRAPREPQQEALFWLK
LKDAISTDRS
>Goc-TLRα3
-LDKFPKLPTVNVRDNHITILP--------------------------------SYMPMI
KVLDASYNSIHNINIDSLSNLTALEIFLINDNDLNYLPNTWGSQTSSLKKLCYHDNPFHC
DCSSLWMEKWTTTFED-LLCSE-D-IVCQNG-----RPITDQG--INEFCHI---FASVI
IGILVTTIIV-TFLLFYFRQVIVLWTRR---RC-----ADDPANKLYDAFVCYADDDFDW
VNDNIIEHLE--PKWKMYIPDRDIQPGELRVILIQEVIETSQRTIMILSQNFLQCTELVN
TFRFAHQQYMVDPSKVLIPILAPNFEVGTMPLFVSCYLKAHTYLEASD------FFMRKL
QLQMPKIRV
>Goc-TLRγ13
VLNCFHSLIELNISGNVI----GQSKWKMFQNCTQMTRLDMSFNKLTSVPLGAFNELSGL
KDLSLAGNDLRSIDI--IQNNAELRFLNFSHNRLDTIPDKWREYLDEVKILDIRNNPFIC
SCQTIEYVEWFQRR--KSMIFQPNALSCVDITGKH--SLMDIDIAEYEECFHPFIIAGTI
PSVVIIIILLIALFIYKRRYRLQYLSLVLKAKMKNYVNAKESKTYLYDSFISYSSNDVYW
MVETLHNKLDDELGYKLCIDVRDFRPGNPIGDEIETGILQSRKIILVITESFLRSGWCTY

ELNIANGELALRGEECLILILREPRPEQLITPTLQRLLKSRIYLQWPEEEDKRLVFWQKL
QDALGQPYS
>Goc-TLRα1
-LSSVPGLP-LNMTGNNFGNITNCYLNDVFNGIYNLKTLTLKDNFITTLTSGPFKSLVFL
DTLDVSNNILNTISDNAFSNVRLLKHINMVNNNMTVLKPEVFNDLSKTGTMFLSGNPYN
C
-CESLPLKNWLNTHQA--QIIDIDITLCQKE--TDTVPIYSMA--DANTCDLTFYVIVII
LS--LLFIIICCILVVRFRQAIRLRLYWFKWRF---EAFEDDSKKKYDAFISYTGHDGDW
VREDLLNFLEG-NNFNICLHERDFRAGELIIDNIDRAIEDSKRSIIVLSNNFLNQDYTMY
EFEASYRDWKLGLRDPVIVILYEALNKEKIAEHLQTHLNTRTYIDKSK------YFWENL
LLAMPRQKH
>Goc-TLRγ7
VFNCFEKIKIINFAQNKV----YNGDLELFLNCTYLEVLDLSFNALNSVPKNTFTNLVNL
KKLNLAGNKFIHID-
FDLRRFWKLESLNMSRNSMDMLSVRTRKELTDLYTVDLHGNHFSC
KCADIDFVEWIQNN--FGMVAMPDSLPCSDERYVKR-RIMLISVTHLRRCWSHTILAAVV
PVALLLLLLFVVLLAYQRRYKIKYLYLLLRAKLR---DHQDRQVYLFDGFLSYSSLDQNW
AVN-LCEKLEQDFGYNLCVDQRNFGLGYQLVDLIVESINQSRKIILVISQNFLRSGWCLF
EMNMANGELAARGRDCLLLVLKDPVPQELISPSLRALLDTRLYLEWSQDPDQEQLFW
QKLRDALGDPRP
>Goc-TLRγ11
MLDCLQSLTVLKMRENQLEVVSNNLNNTLFNNCTQLEYVDLSFNGLTFLPKDSFIGTH
RMKTLNLSGNRLQHSDM--
FPKWQYLDALDLSMNYFTTIGEELRRQMEYLKTLNIQGNPFSCDCQSLDFIEWIQNT--
QVHVTNRDGLSCSLSNQINTIKVLSLNIGQAKKCWSPIILAAVVPCVILIVMITTITIIYRSR
HRIQYYYLIIRAKLR---EQKVQDDYHFDAFLGYSSKDVGW
TIDILYKTLANEMGYNICIDQRNFRPGNYIADTIVASIAQSNKVILVITQNFLQSGWCNF
EMNMAHGELGARGRDCLILILKEPQPENLITPTLKALLGTRVYLEWSDDPDRQRVFWR
LLQDALGQPKP
>Goc-TLRγ18
VIHCLPNLKTLLVSENNLSSLFGKYNQTVFYGCSQLQTLDLSKTLIQAIPSDAFKDLLNI
EHLKIAGNDIQTFDV--LEQCKSLILLDLSSNLLSKLSERMMSRLDALQTVDLMHNPLSC
GCRDIAFITWFQNT--HVHFLNGQEYTCTDENYKE--HLSDIYVFHLNECKMDIIIASTV
PSVCLILIFSIGLLIYRKRWQLHYRYLIAREMVQAFT---TMGNYTYDAFVCYSSQDQTW
VYEQLWTTLEEKHGLKLCIHERDFMPGVDIQENIVQSLEESRNTILVLSKYFVESRWCQ
WEARLARNKLLESPRDNLIMVLLDDVLKPKMNGTLRSLLEMKTYLQFPACPDQQKLFW
MRLKNAISEERP
>Efe-TLRα
----FPNIHSIHFEGNALTTFMYNDLPYAFLPFPNIEFLSLNGNRITHLKFGTFAKLKKL
TNLYLHNNLIKEIDSSVFDDLHLLNQLTLHSNSLELLENDTMDLLSSLSNLTLDDNPWVC
PCDNATFKYWIQQHSE--IISSPFSLKCNE----T---ILRI---DEDLCYKQYLTGPLY
TASLFCLLLLTLVLVYRYRYIIQVIVYKFELRR----KQQESESCAYDAIAVYDSSNLKW
IKDILIPRLE--PKFKLYILDRDMLPGSVQCNEVVENIKRSRRTLVVLSGAEDLQE-IGF
GFDVAHHRVTQERHHRLKLILLHNVAKQDLHANFKAYLTTGQYFSVVD------LFWQKM
LYFLPR-PP
>Efe-TLRβ1

IFQPLLNLQTLNLAGNRLGRVITDIDGQLFRGLSKLRWLRLDNNEMRVMQYTMFSDLTS
L
QMLNLSNNYLSSWAPGIFNASGKLSIVDFSSNKISIVTEQELLTVPTNTSLNLTDNPFAC
YCDLIWFRRWIHNS--STKLAHLQSYICNSPDQMAKKPLLEFNPDDIARCHLMWILLGAC
SG-VVAIMLFFGTMMYRYRWQLRLRLYYAQRRIRGYIEVDEGYD--
YDIYVSYSDSDREW
VRTELMPRFNLLGELRVFIEEADATFGFLEFDTLAEAIYKSKKIMLVVSDEYLHDGRRLF
EREWAIRSRFEKQDDSIIVVCLEPDADVKVPAVLLPIC-
RNQGLEWKQDEAGQEFFWRKL
ADVIQY-RD
>Efe-TLRβ8
IFQEAQNIEKINMSFSRI----ESNLIAPFHHLLKLTDLDVSGTGISNLS-HMFADLPNL
KRLRLSRNPILTLERKDFEGIESLRDLDLSSGKLSTISFESLKVWKNLRVVDFSDNPFLC
DCNFIWLRRWLKRANAKVEVRGWDKYYCQTSKG--QVNMFQLSPSDID-
CFQHWLLTVRL
LTSLIWITATSASALHRFRWHLRYWYFMKTVHAQRFKDEEEPDFFAFDAFVGYSSSDS
NW
VITQLLPRLEQECNLRLCIHERDWLPGRDIAENILESIDNSRKTLLIVSNAFAVSHWCHF
EMTMAQTKLFEDDRDNLILVLLEEIADCNMNPRLQLLMQNKTYIEWTDNNIGQQLFWA
RLRQVLAKQSN
>Efe-TLRβ7
VFRGLSHLRVFNISRSKS------KTIYPFYYFRRIEVLILRDVGLRNLER--ARYNRRL
RYLDISQNEISVLMSSSLARL-KLEVLLVRDNWLSVIDLETLSTWTNLKRVDFSENTLNC
DCKIIWFRRWLQNRQSNVTVENLHLTQCTAPAKAKDKPVHLLEPTDLE-
CFKPYMVAVFL
TAFFAYLIAPTVSMLHRLRWILKYWYFKHKTKAKEYRDIDDNKPYEFDAFISYSESDRN
W
VVSQLRPRLENEFGLRLCIHHRDWLVGRDIVDNVVDSIEHSRKTVLIVSNAFALSPWCH
F
ELTMAQTRLMEEDRDSLVLILLEEIADCNLTPRLQIQMQRRTYIEWTKQSVGQQLFWA
NLKHALAKPSD
>Efe-TLRβ2
----F--LEPQYSSNNPLNN--------------SMTSLDLSGTNLNTLLNGFIECYKAL
RTLNVNQNKLEILTIETFRNLPSLEELRAANNFLTQISESSLKLWTQLKTVDLSGNPFRC
DCKLLKFSQWIKENNFKKLL---DNMQCVSYSSGKRQSIVS-AENRLKLCLEWFLWALTA
TVFVTSFLSTFASIVHRFRWNIRYWIFSHKIKTRRFKQVRDKKHYTYDAFISYSETDSRW
VILQLLPRLESEYHLRLCIHQRDWLAGRDIAENIVLSIEQSRKTVLIVSNAFAVSQWCHF
EMTMAQSRVFQDDRDNLILVMLEEIPDCNMSPRLRMLTERQTYVQWDDHALGQQLF
WVKLQQALAKPAE
>Efe-TLRγ2
LFR-ISNITSLVLINNDF----QN----------SLRPLDLSGSGTSFIPKSMFSELSAL
QFLNLSRNIIESFQV--LPPSGNLSLLNLSDNSIRILTSEMISELESLQTIDLSRNPLSC
LCNATEFIAWLKSTK-KVSFENIEEYTCLHPNGTK--SVNSLNMTELMDCKKSFFIVPLI
IGLLLLLLGVALIVIYKRRMDLRPYIMFIRYLS----PA-ESDNYEFDVFVYFHADDVLW
VRGALLEKLQ----HLKVITPDNFRIGASMADAILDGCRKSRVIVLVLSSSFKRDDWC--
-----TLRSYTSHPGSVIPVVINDTDLSDFEDDYSNLIATHS-IDLAD------FIWNEF

TSRVDRQSS
>Efe-TLRγ1
LFR-ISNITSLVLINNDF----QN----------SLRPLDLSGSGTSFIPKSMFSELSAL
QFLNLSRNIIESFQV--LPPSGNLSLLNLSDNSIRILTSEMISELESLQTIDLSRNPLSC
LCNATEFIAWLKSTK-KVSFENIEEYTCLHPNGTK--SVNSLNMTELMDCKRSYFIVPLI
IGLLLLLLGIALIVIYKRRMDLRPYIMFIRYLS----PA-ESDSCEFDAFVYFQDDDEPW
VSRVLLEKLQ----HVKVITPDNFPLGAAMVDAILDGCRKSRVVVLVLSSSFKRDDWC--
-----TLRSYTSHPGSVIPVVINDTDLSDFEDDYSNLIATHS-IDLAD------FIWNEF
TSRVDRQRS
>Efe-TLRβ6
IFNRVPNLITLNIARSRA------KSLSLLFGLKRLEVLNMRATGVTSLPY--IAKRRHL
RILDVSENEINVLQSPVLQNL-KLEVLLLRDNWLSVINITTLDTWDRLTRVDFSENTLYC
DCQVVWFRRWLRKRK-NTTVENLHLTRCTGPAEVKDVPIHLLHPTDLE-CFSAYLLSVFI
AVFMTTLVTFLVAILHRLRWMLKYWYFRYKARAKEFRELLDQQHYEFDAFLSYSETNY
EW
VVDQLHPRLENEFGLRLCIHHRDWSLGSDIVDNIVNSIERSRKTVLIVSNAFAVSQWCH
F
EMTMAQTKLFEDDRDNLILVLL-------------------------------------

>Efe-TLRβ5
LLWGIPKLKELNVSRSVG------RSIAPLTNQTSLEVLSVRQDDLFDEDVNMLTNVPHL
RHLDLTDNNINVLENIPFHKLKSLEVLLLGKNWLITVNATSLCTRTHLNRVDFSANPLIC
DCGIVWFRRWLNTT--QVIVDNREDIRCSAPEEVKNRILSEIHPTDLE-CFQELLALGLI
LVQMVYLLSLIVSVLYRFRWRLKYWYFRHKTQGNEFENWIETPHYDYDAFISYNESDS
KW
IVTQLSPRLETEYHLRLCIRERDWLVGLDIVDNIVDSIEKSRKTVLIVSNAFALSPWCHF
ELTMAQTRLMEEDRDSLVLILLEEIADCNLTPRLQIQMQRRTYIEWTKQSVGQQLFWA
NLKHALAKPSD
>Efe-TLRβ4
IYEGMPNLEQLRLANSRR------KAAIPFSSLSNLVELNLRGVGIRAVHLNITKNLPKL
RRLDLSDNELSHVRASFFRNL-ELDILLLRRNWISTISKTTFGMWSRLKEVDFSENPYFC
DCRMVWLRQWLRNKTRATAVRNKGRMVCIGPHEAKNTKLYLLKPTREE-
CFAYYRLAVLL
FTLVIWFLAPLLSIVHRYRWFLKYYYFKYKIQQRQLHDLLDKKQYSFDAFISYSESDSKW
VINQLRPHLETEINLHLCIHHRDWLVGRDIVDNIVDSIEKSRKTVLIVSNAFALSPWCHF
ELTMAQTRLMEEDRDSLVLILLEEIADCNLTPRLQIQMQRRTYIEWTKQSVGQQLFWA
NLKHALAKPSD
>Efe-TLRβ3
VFNCIPNLEELLLINVQV------SVRHPFRNLTKLKKLNMGGTSLGDVEVKMIPDLRQL
EWLSLAHNTIQKLRRGMFQNFKTLKVLSLGNNRITTLNVTSLSLWKSLERIDLSGNPFT
CDCQLVWFLHWLSTT--NVTVSDEKQYQCNSPAALKRKSLKKLHPNEVE-
CFEWWLLAVLL
ISITASASSTIGSVLYRFRWYVKYWYFKYKIQQRQEALSTDNHSYQYDAFVSYSKHDTK
WVVTELRRHLEIEEGLNLCIHDRDFLVGEDIVSNVISSIEQSRKVLFIVSNAFAASQWCH
FELIMVQTRMLENDRDNLVLILLEEIDDATLSPRLKLQMEKQTYLEWTSSEVGRQLFWD
RLRQAVSRPPE

>Hro-TLRα3
-LKTLPLLP-LTFSRNMISSMD--------------------------------FYLSST
TVLDLSYNELNQFDLSTLMFSTTLEELYLHSNNLVSVPPEFLQKSPKLKVLTLHDNPWD
C
SYENKFLKSWMMNGNV--SLLHENSILCRTP--LSGKSIFFVK--DEEANDP-RNRVILC
ILIPLFAIEIFALAIFLILKKFKVKLYYLNIHPR----ECTDEYMEYDAFISCAFSDRCR
AIE-LVSTLEN-RGYKVCYPERDFIPGEPTTSFVSK----SRRVIYLLTLDFVNTPRCLF
EFQISLQRNLEVKHKRIIVLLDSSLKVQLLPNDMFNFLTTHHCIDLLK------NWTNQL
FYSLPIKPL
>Hro-TLRα1
-YESLPLIPLLYFNKNLLTSFN--------------------------------HVFNDT
KILDLSENKINEISPHTWVQLIQEDSVFLHNNNLSYLPRILEKMNTS-KRVTLHGNPWSC
TCQNAWMLDWLKSHVH--VV-KPEKMVCAYP--HESKSLFEVDF-----CYS---ISCVT
VGIVVLIVAVT-----------YVWYRRFGPQKK--PPVPPNPKLTNDVFIFCSDEEQVP
LVKEIIRWLENEHRFSTICGLRDFDSK-PKVVNINEALTTSKRIIFIVSKDFLKDNWCIS
GTMSA-FSLVEDKRRFIVIFCGVHLQSTNIPVELEIYIRTYTYLSFDD------SFWKKL
LRAMPKE--
>Hro-TLRα2
-MSNLPMLP-LTFSHNSIETID--------------------------------FYFNNT
VVLDLSYNKITKIDLQVFKSLKVLQELHLHSNFLTTVPRDFLKNDRMLKHISLHNNSWDC
SCGNKWLKQWMMNQSI--TLLTPDSVLCRTP--LSGRSLFSVS--EEECPKP--SRLLIS
LLIPLLTGVLLLLALFVLIKKFKVELNYLNIHLR----ECIGENMIYDAFVSCSYSDRRR
GIE-LVRLMEG-KGYHVCYHEKDFIGGQSIAANIVEAITFSKRVVCLLTSNFLKSTYCMF
EFQTSLHRNIELKRKRLIVLLDESVEVEVLPNDVHNFLTTHTYIELSS------KWTHQL
FYSLPLNPI
>Hro-TLRγ1
FLNFIS-LGELNVEKNELGEQMSDMFGLTFQCYSNLTILNLSNNKIKKLHKLSFKNLRQL
RILNLAENSLQTIE-FEISHMKYLQYLDLSRNLLVSLADDTCYVLSGLSSTSLYGNPLQC
NCETIKFMKWVQSK--KVTINNKNHTNCQNRSS-KFITLSNLAVNELRFCNLPLIIGGSL
VG-
LLIICSFLGFVLYKYRWDIRYFLMSMQKSKRRCTSFERNRYYKYDAFVCYEKSDRRW
VVTELLYNLEADDRFLLCIHDRDFELGLGIKHNISMAIHASRKTLLVLTNNFLKSKWCRH
ELEMASLESLDRECNLVVPVFLEPV---ETWDSLSWLTKRYTYLEW--------FAYDKL
VVALN----
> Cgi-TLRγ2
AFASLRKLEYLDISNNEH----------------GLAVLGLNQTNIKVLKVNI-------
----LQRHHLSYLDN------TSLLELSIATNRIETLEPCVLSRLKSIKRLSIARNRLIA
AAYVLEYHSLV-----NVEVINA--------------SLRNFTIS---GCKE--------
---LILKNTVYHPALYRNRWKIRYMRYTLFQRAR---SSSSDDLFLYDAFVSYTSKDRDF
VIKDMIQKLEQDNGVQLLIRDRSFIPGEFKCQQIVRSIQESRKTICVVSKRYLKSAWRDY
ELNMARVEGVEVRKRYVILILLPEVCSGGYPKKISDFLKRDCFIEYPDDPAGYEEFWQR
LCSALQENQD
>Cgi-TLRα4
-LTKIPGFP-VSLDRNNISEIYHSTLNNSFIGLLQLKTLYLNNNELQEINRGVFNKLWNL
TELHLEYNNIAYIEEGAFSALTSLSTLFLDHNLLISLPQSATNHF--LSNIRLGENPWSC
SCDVMAFIPMVMNRSM--VISDYSDMFCKETGE--NFSMKDVLVK---RCTSNILKILVI

VAAIILTTFFIIIVICLWR---PIVLFHRKCKCR---RYPEDGDKSFDAFLAYSHKDDDY
VTREFIPRLENELKYRLCVYYRDFPIGGTIADTVASSINRSKRTILLVSKHFNDHEWRNT
AFQHSFGGLFKQKDNHLIIVLLDDAKGMKLDRQLKVLVKSHHVISYRD------CFWEQL
QYKMGSSKR
>Cgi-TLRα3
-LYNIPSLRH---WRLNFPKL
KVLDLTNNHISDLIIDHDPDSSDKGVINLQYNNLTSVSDNQLKNFHSF-YIDVQNNPFSC
GC--MRVKHFILNNTKSSEYSYLRGLKCQNP--VAGRELITLS--DADGCGSQ-SGPIII
LCVLVFVLFVCLVVIIRYRVEIKILAFRFNI---PCQQQDNLDNKKFDAFVAYSQQDSDW
VLKNLVWQLETLQRFHLCLHQRDFTVGAPIAENIINSIERSRHTILVISSNFVRSEWCLM
EFRTAFHQSLIEKRRHMIIIVMGDLPHGELDTDIKRCLKTLTYLETHD------LFWDKL
VYALSDKQR
>Cgi-TLRα1
GFQDMPNLEMFGILDRKI--------NEIFGSFQKLHTANFSDANLEFIPANWFRQFRSL
RIIDLSHNRIKEIPYRR-NHFGKLIKLILRHNNISRITKTLIEKLSNM-AVDFSKNKFVC
ACESLEVLQFVRNQIEAINYHYLANETCYYPSSLQGMPLRSL---DSLLCPNSWTFQELY
IGLIVLTFITIVCLVVKFRKEIKILTYRLGIRF-PHR---SGRLKEYDAFVSYSALDESW
VMGTLCKRLEG-PPLRLCLHHKHFVLGACISDNIIESVEKSRHTIIVLSQNFLQSEWCLL
EFRKAFHQTLLERRRHLIVILMDQINLDTLEPEMNYFLQSHTYLKRTD------LFWDRL
IYAVSDP--
>Cgi-TLRα2
SFKNLQ-LETFLTESNRF----------------ELRILNISHSSLYYVPENWIIYFPKL
EYLDMSHNKIQDIVLSMYDPTSARLTLDLTFNDIRQISVRFLEKIARL-YVIIDNNPINC
SCTDMRVLEYIRNSVK----QYIRDLKCQFPENIKGRRLRDL---DNDGCG-KMLPIIVV
LSILICFLLIFLFLIIRYRLQIRLFCARLSGISN---DMDAEKSFKFDALICHGLFDEEW
ARSTFIENRHK-
SHLKLGFYREDATDQKNNFEKLIDQMKSSKYVVVLLSRQFLEGEFLTP
GFQEALQQSNEHTRKRSILVLMDDIPTQEETICLRRSLQTFTCIHKND------RFTDKF
LYLLSSK--
>Cgi-TLRδ2
ILQGVKNIRQLRAVNVQFN--FNLISESLFKNLKYLTNLDISRNSLNFLPQSLRDQKLSL
KELNLDHNMFSSLS-SSLKQFTNLRKLYVRYNLISKINEKDQELFKSLTLIYIEGNPISC
TCSNIQSLKWMKDH--QHLFSDLSKTKCVGSNNL-TVELNEW-LLKFEICQADWLIFSIV
LIVSTLTMLIILAAIKKYHVHLEYVILRVKQRLMPVGHVCVEGDFQYDVYISYNDDDTSW
VANNLNPKLE---NIKAWFKEKDSIPGGWESEEIVNCINDSRKVMFIVSESFLDKGWHSY
AVQMAITHAFHNQRRSIMVIIKDGLPLERLPKEFKHIWWCIEHLRWPEDETNDETLLLNL
SNVLVSE--
>**Cgi-TLRδ1**
MFQGVRNLHHLYVLDVGLNNTAHSISNSLFKNLKNLLTLDFSKNGLAFLPSLLMDQKHS
L
TEIRLDHNRFSAVP-SVLTELKELKTLYVRFNLISKFSRNDQRLFQSLSSIYIEGNPITC
ACTGVQSLKWMKAH--QNIFYDLNKVLCVESKIP-IVQLYEW--RKFENCQTDWLVFSVC
LLFFTIVSLTIIASVKRYRVHLEYVILRLKNRWKGV-QKSNEDMFLYDVYISYNDADCSW
VIETLYPKLE---NIKTWFGDKDSIPGRWKSEEIVGCINESRKVMFIMSESFLERGWHSY
AVQMAITHAFHNQRRSIVLIIKDGLPLDRLPNEIKNIWWCIEHFRWPENEQHDEMIFSTL
SKILKPK--

>Cgi-TLRβ4
LFK-APNITNIELFDNQIS---GSTLKTLLWNLIKLQKLNLQGCGINYLARGTFDRMPDL
RTIILKGNSLYGWDPTMFNKLFNLRALYLSGNSVAVVNRTSLIGKINLKFIDLADNPFAC
TCQQLWFRDWLKTAKNITVAFYPKRYVCRSPPKWDNTLVALFNYTEED-
CREPWILIGSV
LGSVVFVCMVVVIVIYTHLPTVRNIIYLIRLRRKGYVRLVNSEEYMFDCYVVSCETDEQW
VFQTLSSTLEVKHSYRLCIPTRDFDIGASIADQIEEKMRECKKIIIVMSNDFAQDEWCQF
QLEKAQERIRNQGEEAVVSIMLHDIDHKHMTSTIKNLLRKSSYATWVKGKIVSKLFWDIV
VAAIEK-PP
>Cgi-TLRβ3
LFNNTRNLRVLDMTGVQFSHNLEEKMFQLFKPLTGLEELTLKKTSLSTFPVSVFQFMP
NL
TKLSLQDCYFNQSYLRKLSAPASLKVILLDNNLITSINETNI---NNIDQMSLKSNPFLC
TCDLVFFRKWIETNS-KRLLGWPNDYTCNLPQEWKGKNLADFHLSYLS-CHPPYIIMAIS
ISFAVLAIATVSCIIYKKRWHIKYYLYLLRAKKRGYEVL-GGDDFAYDVFVAYNSDDRIW
VISEMIPRLENEEHLKLCLHDRDFQVGKLIVDNITDAMHRSRKILIILSNSFAQSHWCRF
ETMMAQLRSINHGENTVVVVILENILTKNMNNSLHMLLKSTTFIEWTNERAAKEMFWTR
LVSSIKT---
>Cgi-TLRβ1
SFNHLQSLKHLKFDDNNLGGLISDNIGTLFAGLHKLETLSLSKNFLHNLPISIFKDLSSL
QTLTMKSNRISGWNNGLFKQTSALRSLDLSDNSISLVNSSSLADLSNFQMLNLSNNPL
ACTCDLRWFRDWVNQT--RVNIANVGNYVCNSPNVWKGKPFLSFDRTKIN-CV-
LYFVVGVS
IA-SGLAVLVFCVIIYKKRWWILYRCYRLKNCC-RYQPIQDGQELVFDAYISYADDDYKW
VLEQLLPDIDSKGEFKLYFHDRDSVPGSSMISSISDNIEMSRKVIIVLTEKYLSSARHKF
EIDLAVMLKSQGVIDDIIVINVCGVSFACIPKSLQRKVSKDEFLLWKDDVDAIWLFKQRL
KAELKR---
>Cgi-TLRβ2
IFRYCRGLNVLDMTRIRLTTYDGVMLYELLHHLTNLTKLVLQSTLVLTLPENLFSRMPFL
GSLHLDHCYLSQWKLGVFRNVASVKTLYLDHNEIAIINQTSFELLRGLKQLSLGYNPYL
CTCDMVWFREWTGNNT-KVMLNWPYAYKCKSPREWATKLFSDFSLSYSY-
CHPPYVIAAIS
TAAGVVLIVIVVGLFYHYRWHIKYFFYLMRARKRGYEPLPGDDDFIYDVFVAYHSDDRV
WVISELIPCLERKEKLRLCLHDRDFEVGKLIVDNITEKINSSRKVLLLLSNNFIQNRWCKF
EMAMVHARNVEEDRSDIVVVILENIRTQNMSNSLHVLLKTTNFLEWSNKKSAKELFWK
RLVASVK-PES
>Cgi-TLRγ1
FFIPFVGLEILNLSNNALSQMFSDENGDFFQSQRRLTDLDLSLNRIAHLPGHVFQHNSKI
SRLNLSFNSLSDFNV--INHMKHLSQLDLSHNQLTQLSKNVRASLDAIAKVYLLGNNLKC
ICGTLDFLKWLRDSK-SIYFVGINNYTCLFENA--AASFNEIIVQVLEKCSSTLIIVLMT
TLIIVTMTTTVSRILYRYRWKLRYMYYVAKEKYKTHSEEKDRSSFRFDAFISYAEEERLF
VFK-LVKYLEEKCNLRLCIHHRDFIPGTGIADNITNAIHCSRHTVCFMTSHFLQSHWCMF
ELNMARMEAIYARQNVLFLVALEK-
TMKHLPLQLMDLVDSNSYLEYPGEESGIEAFRTKLGETLAS-SD
>Obi-TLRβ5
IFKSCPQLTNLILKNVSN------YPNDLLKPLTKLENLMITDGQVSKVPD--ICNMNNL

TELSIFYTTVSKWNNANCSVMRVLRKLVLKDNKIIYVNPKLFSHLSNL-HIDLSRNPFVC
DCKALWFRDWSRKN--AGRLKNYRNYRCFSPNSLGHIHLRNFSLSWDY-
CENSIAIAGVS
VGVLAVMFVFLAILSYTKRWSIRFCIYQSLVRKRKYKALVNNGRYKYDAFICYCSTDVS
WVLNKLLPIIEEENHFNLCLHDRDFLVGNDIVDNIVDSMQQSRKVVLVLSNDFAQSSWC
QFEASIAQQKILEEHYDIIIPVLLNEIPSNLQTKRLGVLLKQKTYLEWPNDEQYEGMFWE
RFIGRLNANNE

>Obi-TLRα2

-LTQL-GLP-IDLSGNKLNYLYNSIVNKTFIKFTNLKKLYLQNNLIEALQQKSFEGLKNL
LELILYNNKIQYIPENTFSETPKLKYLDLRNNKLQTITSEMF---KALQKIYLSDNPWSC
ECNDISFEKIFAKDTE--LLVNGEQIFCRKYDA----NIFNYGQE---FCQNVLISSVSA
VSSVIILIFLITFLVYAYRQEIQLLLFHFGYRFK---LIIDEENKLYDAFVSFDNSLDLF
VLNELLPQLEQNPPFKLCVHFRDFEVGLQITENIINSIENSKRTILLITDNFLKSEWCKY
EFQTAHYDGLSQKMNTLIVVLFENINEELLDPDLKLYLKTKTYLKYDD------WFWNKL
RFALPAKKD

>Obi-TLRβ4

IFKSCPQLTNLILKNVSN------YPNDLLKPLTKLENLMITDGQFSKVPD--ICNMNNL
TELSICYTNVRKWNKPNCSVMRVLQKLELKHNKIRYVNQELFSHLSNL-
NIDLSNNPFVC
DCKALWFRDWSREN--AGRLKNYRNYRCFIPDTFHHISLENFSLNWDY-CENFIAIFGGI
IAVLVVIFVFFAILSYEKQWSIGFCLYHSLVRKRKYKTLVNEVQYKHDALVCYCSADVN
WVVNKLLPIIEEENHFSLCLHERDVVVGNDTVDNIVDSMQQSRKVVLVLSNDFSQSSW
CQFEASIAQQKILKDHYDIIIPVLLNEIPSNLRTESLVDLMNQKTFLKWPNESKYE-------
----

>Obi-TLRβ2

LFR-FPSISLQLIVTTLLSD--SKSINQSLAFLPNLVTLQMSFSNLKTIP-KVICNMVNL
TSLNLKGNAIVWWNDTNCFVMKKLHFLSFSENRISIVGDKTFLLINNL-RWDLSLNPFLC
NCENAWFKSWVEKNHQKFLYYPK-DFTCDTPADLRGKQLSDIDLGNNI-CGVVGITIGIV
LGSLMFVFVVVASISYYKRWALRYICYLLKSRKKQERSQQDEKSYVYDAFICYHNSDSK
YLLEKLQPKLEEENNFRLCIHDRDFVPGWDIVDNIVESIEKSHKIVLLLSNNFALSEWCQ
FESTMAQQRLFNEKKNTLIPILLEPIKIKNQTSRLTILLKEKTYLEWTDDKNGQKLFWARL
LNTMRGP--

>Obi-TLRγ

FFTFFTGLRLLNIENNAIGQVAEEGFSKAFLNLTNLEELYLSNNHIRYLSNNSLLQLKNL
RILNLHINLLESFDV--ISHMLNLSYLDLSKNILQELSENTFNAIEKISTVNIQSNNLKC
GCAQIRFLTWLHKVRNHLKI---MYSKCTHPNGTV--KLTDLIITYLNSCS-SFIIITVA
CLIVLLGCFSFGALVYHFRWKLRYLYYMIRERY-AYQRI-QTGEYLYDAFVSYAEEDRGC
VFEYLIPELEEKDTFKLNIHHRDFPAGKQIAENILSAIQSSRKCLILLSRSFLSSEWCMF
EYNMAKMECVHAERDLVIVIMLEELSVDILPLQLQHQIKMQSYICFPTTNPTSDVFWNN
LKKSIQE---

>Obi-TLRβ1

FSKELTRIRKLYLDSNKLGKFLTDKKGFLLSG---------------QLSKKIFQNNKHL
KYVYFRGNKITGWENNTFVTNLTLMELDVSNNFIFTFDSDSLKYINRKKKFNVTGNPFA
C
DCNLRWFRDWLNTT--TVDIVDKNGLTCNSPPDWQDKQLLDFTRSKID-
CTDLYYILGGV

GG-GFLLTVVIVLFAYTKRWYIRFKIFKLYQYVQEYEAI-PGDDMYFDGYISYSDKDADW
VEKYLMHTFDNNGNFKLCFRNRDFAYGKYIIGMIESSLAVSKKMIMVLTPYYKKDKRCE
FELQLGIMKLNI---KNVMPIVLKNLQPNQIPNSLKEIFETNKFIEWDNN-----------
----
>Obi-TLRβ3
IFNACRHITLLKFQAVSI----NASINELLMDLKNLEYLELTGNTMRNVPD--VCDMKRL
HTLILSRTWIRKWQTTNCTVMNILRVFSLSYNRIFNVNKTSFALFSNL-KWDLSHNSYIC
NCRILWFRDWMRQN--SARLHYPKSYLCSNPAPVRFLQIAKYYVSWDY-
CANPIAVSGCV
LGTLSVIFIITGLVSYIKRWSIRYWVYLFFARRRKYQLL-ETSEHNYDAFVCYCGSDVGW
VTKYLLPILEEENDLHLCLHDRDFAVGNDIVDNIVDSIQQSRKVVLVLSSDFAQSQWCQ
FETSLAQQRLFEEKKDIIVPILLEEIPTELQTMRLALLLKQKTYLEWSNETRGQMLFWER
LVEILLETKE
>Obi-TLRα3
-ITVFPTMP-VWLQFNNITKLV--------------------------------PYLSQI
THLNLTKNSITSLNYTVMRNMVNLKQMILDWNLLTTLPKGIQNVQ--FEVLSINHNHFFC
DCTNIWLKKWLQKSRE--SILNWRRIVCNTV--DKVLDIVVVP--NDKICNPKTLTLGLS
LALAILILLVCLFLLIHYWLEIKVILYYLNIHPSLGSANTLNEQKKYDIFISYPDQTYQF
ATGPLLSTLQS-RGYSICLPDRDFVVGSAKEENILRAIKSSVRTLIVITKSHVEDEWQLF
TLRTAVQCSLKKPFNYLLCILDG-VDKSKLDLETQAYVTSHVVIDKDD------LLWKKL
FRSIPPART
>Obi-TLRα1
-LSD-SMLP-IYLSGNRLISLSRSNINGTFMTLINLKQLYMHDNDLTILTKETFQGLENL
EVITLNSNSISYIAPGMFAPMPKLKIVDVSSNRLHILDNSFL---KYLESIAIHNNPWIC
KCPFVMLQELYINKPD--LVVLSESVICDHEDV--AYPLFEF---DVQHCL-KVICALAI
FSAVFLTLIIAVISIACYREELKVWLFQYGWRIP---AKLDDSNRRYDVFVAYTSKNAMF
VEHELTPRLEREPPYQVCLTYRDYDVDISYAQNTINCIQNSKRTIMLVSNDFFQTEWFR
YDFQINNHDILKTLSERLIVILMEKVDRKKLECDLMFYAKTKKFLKYQD------HFWDKL
YYMLPKVRG
>Bgl-TLRγ10
-----------------------------FENLSDLKKLHLI------------------
--------QMRDI-------------------------------------TVNGATLSV
----------IKI---SLSGDHAGDST---------------------------------
------------------SLALPSSLFAFT---------QD---YKIDVFLGYSDTDYRF
PCQDLRAYLEDTLKLTTFLNDRDLLATLNKASGIVEAINSSWRVLLVCSEGFLKDEWSL
F
TMRSAMYAQSPANPGRVV-VMVHQRCLRLLPTELLSAVEEDNILV----------SEWK--
----
>Bgl-TLRγ3
FFDSFPEVEVLVLDHCELDSEFSQHSYSLFRNLVKLKQLDLSYNALDILLPNTFSANLNL
RSLNLAFNRFRTIP-FDLSQTLGMNKLDMRQNSLETLSSKDMALLDELQKLLISGNVLSC
GCEHIQFLQWLHLT--DVRLDENRNYTCINNQG--LSSTSAYNIEVLWECWGYFNIALGM
FAFVNIGFVFVFMLTKNKTLIISGVLQLFT-EFKKRP--V---DYQYSVFIGYSDFDYQF
ACLTLRKFIEDDLKLSTFVGDRDLLPSIAMAEGIMAAMDSSWRIVLVVNKSFVNNNWFL
F
MVRSAVFSVSPANPLRVV-ILVEECCLPRLPSELLSSVPEDNVFVV--------TEWK--

>Bgl-TLRα2
-LTDVPKIP-LRLDGNNLPSLRNSTVNNTFKGMKSVRSLFLNNNLLTIISPGVFSGLENL
ERIFLQNNFISLIDPQAL--LPYLYLINLRENDLNTLPIDGLGFVRELKRFSLSQNPYSC
QLDFVCFVLFIRDSAD--CIEDISDIKCSSNSL--GFTLLDFQIE---LCSE--TYALIA
ACVVIAFGLALLIVAYMNRDFLQVLCFRFGLRVM---KATEDNDRPYDAFISYSSKDEDF
VIHQLAPRLENDKKFQLCVHYRDFPVGACIAETIVRSVEASKRTILVVSDNFLDSEWCR
FEFQTAHQQVLNERRNRVILILMHDLDTEKLDSTLKVYMRTRTYLKYDD------WFWEKL
MFAMPDVQH
>Bgl-TLRγ23
FFDTLSTLKKLNISNNLLGSFLVLVSPRIFSSLRNLTVLDLSENFIIDFTCDLFSNLTSL
EYFNISKNALIRFEV--ISRMSNLIFLDFHLTRMTGLTSEFRDSIDRLSSIDMSNAPISC
NCKNYDFMTWMTSS--KAFSQGFKNYICVYPDQTGHV-
VNDFDMNLLNQCASVLLFSMIA
IAMIVVVGAVVGGIVYKYRWKLRYLYNAAYLQFKSSRRG-
EDDEFDYDAFISYDQEDGVF
VTQTLVPELEKR-EIHLCIHASEFTAGEYISSNIVKAVNRSRKTVVVLTQNMLSSYWCNF
EIQMANMEALHTGRRVLVFLLVDNIPTKDLGLELLYYIRSNTYIPFPKDFNGMSWLWDK
VANDIRND--
>Bgl-TLRγ20
FFKNLPTLTYLNLSINLLSRCHNVKKKYIYEALVNLEVLDLGLNNIDEFTPHILDHLISL
KKLFLDYNPLKSFDV--ISNMPQLEYLSLRHSRLHRLSVYTMKAIDEITSIDMAFNPILC
ECSNLDFIRWMTAS--SAFDPKFESYFCMYSDGSMQF-IDDFTLMILSECASVIIFFSVS
SGTTFLIILILIAILHRFRWKLKYMYYAAYLHYKSAR--DNGKAFSYDVFLCYHEDDESF
VLDTLCVELEKR-
GLKTLVHKRDFVSGKPIVSNIVEAVNCSRKTLVVLTDNMARSKWCQF
EVQMATMEAVSYKRPVLIFLLMSDVPCCIMGAELSYCVQNNTYLQYPSPSSEMDNFWI
KLVSDLKN---
>Bgl-TLRγ19
FFENFASLEFLDLSANTFGRKVRKGSKPIFSSLKNLRELNLRFVDLITVDKNVFEGLENL
EILHLQLNGIYYFEV--VSYLKKLQFVNFSFTELTGLRPQVTNFFDSIATLDFSETPIHC
YCANLEFISWLSRALQYIRFQRLKWFKCVYEDTTEKY-FHDFLHQFLGECTPVTLFFIVT
SATFLLVCIIIALVVYRFRWKLKYFYYSAYLYFKSYKRFHDDKDFEFDVFVSFANEDERF
VLKEILPELTTR-GLKVHIHTTNFRAGEYITTNIVNAVQCSRRTLIVVSSNLQKSQWCHF
ELQMANLESVHTGRPVMVFLLMESLPEDVLSREMLYHIQNNTYLQLPDEVRVMDIFWT
KLCSDLKD---
>Bgl-TLRα3
-LEKVPEIP-VYLDGNSLNKLTRSYLDGLFDNCTSLLHLRLDYNYLISISKSLFDKLIEL
RSLYLNDNLINFIAKEAFANLNSVEIITLDKNRLIMLD-------SSLKSLTLSGNPWQC
QCNTSTVLRVLHALND--IIVDRGNMCCYYVGTVQ--KLSELDRTPYELCVDTLLVCLVV
AMFVLLLIVVVLVIIFKGR-EVQAWVYNLGVRVK---DKTDAGNKYFDAFISYSNKDSEF
VSKVLVPALDE-KGYRLCVHYRDFPVGQNITDTIFRAIEESSRTIMLLSRHFVESEWCRF
EFQTAHYHILKEGSHRLVFILLDDLSDDELDPDLKVQLKSKTYLKFGD------WFWEKL
FFALPDVRK
>Bgl-TLRγ22
FFDSFTSIRELNLSNNLLGEFFQSNETLVFSKLKNLEILDLSSNGIHLLHFDLFMDLPRL

HHLNLAFNTLTTFGV--ITKLSKLMYLDLTKTGISKIPETARTFIDGL-SVFMGKCSISC
ECDNLDFLLWMVNS--KAFDKTFKNYMCFYMDSSS--PITDYTIEILRKCTSEMLFFMVG
CGTLFLFFLLLFGIIYRFRWKLRYLYYAAYLHYKKSGGE-GGAKFKYDAFVSYDHADEET
IVIHVCNELEARGLKLCVHGRDFRAGDYIASNVVKAVCSSRKTLVVLTKNLMNSYWCK
YELQMANMEAVHTGRQVLIFLLVENIPQGELGVELLYNIRNNTYIPYPTEPAFWDALWN
KLANDIRD---

>BgI-TLRγ21

FFSELSSLLHLNLSSNLLGSFFRYESETVFYPLTNLMTLNLSFNDISELRPNIFANLINL
RQLQLQKNNLQKFDV--ITSLIKLVRLNLKLNRLSTMSSNITDHIDTLKVVDLSFSPISC
QCNNLAFINWMVNS--KAFHPNFINYQCVDSNTIQ--NITDYTVEKLNECSSVTIFLISS
GFSFVILCFVIGSVIYRFRWRIRYLYYAAYLYYSKTNSG-RDSDYKYDAFISYDQNDWKF
VVNKLMPEMEKR-RLKVCIHSKDFVAGDYIASNIVKAICSSSRTVVVLTRNMIKSYWCGY
EIQMANMEAVHTNRKVLLFLMMEDIPSSELSVDLLYNIRNNTYLQYNQDGVHMSRLWD
KLAYDIKH---

>BgI-TLRγ18

FFNNLTSLKHLSLFQNLLGDCLNDKNGLIFSQLTELKVLNLSFNNLYYLGWEVFQGQAD
IEVIDLSVNRLDHITF--VSHMRKLRHLDLHKNDIETLPTGLTDHISSLKTLDMRQNPISC
GCENLDFLQWVVNT--
RVFGSDLYLYYCKFPDSDRAVRVPGYVVKRLVSCSSAVLYTVVS
CVTVLIMLILLAAVIYRFRWTLRYWYHAAKLKISSNQQM-DSDQFKYDVFVSYASKDIDF
VVKELCPRLKER-
NITVYVHGEKFKVGCYIADNIYTGIRKCRKTLVVVTQNMLASRWCNY
ELQIAREQARNTGRNVLVFLFLEELPTSRMGMGVLTHIKSSTYIMYPKLPQHRGAFWD
KLADDLRSS--

>BgI-TLRα4

-FLEMPIIPNLYLDHNPLQSLN--------------------------------PYLSRL
SEIYIDNCLLTTVMPSAIAALKNIRVMTLHNNLLQKLPTSTRNITEKATNITLHNNRWAC
SCESLWLPRWISRHKA--VLWKPGNILCDYFQK-----LEDVS--EADNCK-SAMDNFLT
VILFVLSTVATVILFFCYNTDICAIVYKLGIEFR----LYGDQYCPFDILISYGQDNYKW
VVDTLVPYLEKPGGYRVCLNHREFPSSDCVLETLPTAVRLSRSAILVLSKEFLQKEWC
ML
EVRVAIQRLLLVGS-KLLIICMDKVNVDELSPELRAYIHTHHYLRYDE------DFWVKL
DLFLPRKLI

>BgI-TLRα1

----IK-LRHLDESVAKL--------VTLFDALYVLEHMNFSTIGITTFPREWRRFFPKL
TYIDLSNNFISQVQFQNFPS-KTVVTFNLQRNNITVINMDVLNSWEKL-EVDIRNNPIHC
GCELESFLPHLQDTTTLAPYEYVKEMECSTPDALKGRKLYSL---HSSPCP-VYQVALIA
LGVTLSFLLVLVIILVRYKFEIRILLYRLHVRL-PCDADE-RHSKTYDAFISYSNDDDSW
VFENLVKFLENEKPFRLCIHQRDFVPGKTIFDNIVDSIEASRHTIIVLSPSFMKSHWAME
ELRQAYRQSLVEKTRHLVVLLLHKV---NL--N---------YCSF-------------------------

>BgI-TLRγ16

FFMNFSSLDQLFLGHNTLGDFLSHYNITPFIYLKQLTRLDLSYNGLTKVYRNLLSGLNAL
QELHMEENIMWDFNI--IDHMSNLRLIDLSHNQIKELPIHVREHIDNLKLIDLSFNPIRC
ECQYLYMILWMVSS--RAFNPAFENYMCVYPDGSYKI-IDDYTLQYLNACADYSVLLVVI
FSTLTMIILVIAGILYRFRWHLRYLYYAAYLKVKEGHHNQETRSYVYDVFVSYAHQDETF
VVQRLMPELSNR-GLNVFVHGRDFVVGHYIASNILTAIRESRKTLVVLTKNLINSTWCNY

ELQMANMESVHTGRQVLVFLIKDSLDITDLKTDLLYHIKNNTYIDYPHGPLALNLFWDKL
SLDLKN---
>Bgl-TLRγ14
MFQYLDELQELRLRNNQLNNFIK-TRKPVFQYLKQLKILDLTNNALTVVQSSIFEELGSL
EIIDLSRNNMRHFNL--LTNMSSLNFLNLSHTQLSSLSVETRQNIDLLTRVDMSRNPVRC
ECDNIDFLKWMVSS--RAFDVNLTDYMCQYKDTST-IVIKDYTLVYLARCADSTLFLVVL
SVTLCMVSFVVAAVVYRFRWRLRYMYYAAYLVVKGKRKDNEAELFRYDVFISYASEDE
EFILGKLLPEFDSR-
DLRVLVHGRDFAVGEFIASNIVTAVKESRKTLVVLTRNLLNSTWCNFELQMANMESIHT
GRPVLLFLIKESIPTTELTSDLLYHLNKNTYIVYPQ--EITDVFWDKLARDLLQ---
>Bgl-TLRγ8
FFDGFFGLEKLFLSKCELQRDFALHSSRVFQNLSNLQSLDLSYNYLNDLSQGTLYYNP
KLVWLNLSDNQFNRIP-
FDLKDTPNLLELDVRNNAISTVSKSITTELDQLANFWLSGNILSCGCQDLNFLHWLSST-
-MVTLDQGGNFTCMDRNG-ERSYTMRYHVDTLWECWGFLYLAIII
LCFYVTGVFLLVLVQRNKTFLVSFFLQLLG-NFKLKR--G---DYPIDVFVGYSDEDYHF
PCRDLRLYLEDVIKLKTFLNDRDLLASLSKASGIVDAINSSYRILLVCSESFLKDDWSLF
TMRAAMYAQSPANPSRVV-VVVHESCLHLLPTELLSVVNEENILVV--------SGWK--

>Bgl-TLRγ7
FFDELTGLEYLALSKAGLNRDFSSFSRRLFQNLSNLTRLDLSINYLNALSKGTFSPNSKL
QWLDLSGNQFKDIP-FDLQYTPNLLELDVSSNALTTIDDDIARDLDHLVHLSLGGNILSC
SCSDLRFLQWLNLT--SVTFDHSRNYTCLNKDG-EKAYTLFYDLDSLWECWGFLYVAVII
VCLYVIGFFVILLLLRNKHFLVSYFLKILG-NIKLKR--T---DYPIHVYIAYSDIEYKF
SCSDLREYIEGTLKLNTFLNDRDLISSLNSAADIVKAMNSSWKILLVCSASFLNGDWAM
LTLRSAIYAQSPTNPARIM-VLVHQNDLLLLPHDLLSVVDDENMLII--------SEWK--
----
>Bgl-TLRγ6
-------MENLALSNCRLERDFSQHSHVLFKNLTRLRQLDLSSNSLNYLSKNTFLFNSHL
QFVNLSRNLFREIP-FTLRYTPELRALDLSVNSLSSIDVSTTKDLDHLVKLYLQGNVLSC
GCNDITFLQWMKTT--LVTFDLNGNFTCINEKG-ERTYILFHDLESLWECNGFLYLSVII
MCLYFIGLCIVFIIYRNKQFLISYLLQTFV-GFKSTR--K---DYKIDVYIGYSDRDYKF
PCKDLREFFENSLGYKTFLIDRDLIASVDKASGIVDALNDSWRILLVCSESFLKEDWSMF
TMRSAIYIQSPANPARVV-VLVHKDCLHLLPTTLIGSVNEEKIIVV--------SEWK--

>Bgl-TLRγ1
FFDDFSSLRYLILQSMMNEDFFRVSIDRIIQNMPELRYLDLTDNKLNFLPPNLFSRNSHI
THVILAKNRFSSFP-ITMDLVPNLKTLDLSGNAIIYLTEEETSSLTKHSYLLLAENNIAC
VCSQIKFLLWLNI---TF-LDNKGAYSCTSQDG-QLILTVLWDVLGFYQCYGYFMISIVL
LLVMSFIFLMAYLVHRFRTAIEAYLVRIFIKAVRMKS--SD---YKTHVFIGYADEDVGF
VRHILLRYLEEDLKVSTFVHHRDLGPGYTDQQ-MFESISDSWRILLVITQRYLNNYLSDI
IMKYASHSMSPANEKRLV-LLVQESQLYNIPGYLYDVLEDSRIIV---------SDLSA-

>Bgl-TLRγ5
YLDTLPALENLALANCQLDREFSIHSGRLFQNLTRLQQLDLSSNLLNYLSTDTFMYNKH
LKWLTLAQNQFREIP-FSLKYTPELEVLDLRQNSLNTIDMASIHQLENIVKLLLSGNDLSC

GCNDLQFLQWMRST--AVTFDQDGNFTCTNKDG-KTTYTLAYDIEYLWECTGYFYIVLIV
FCLYLIGCSIVFIMMKNKHFITVYILKRIF-GIEHTR--R---DYPIDVYIAYSDTDYQF
PCNELRQFIEQSLGMTTFLIDRDLNASFDLALGIVNAINKSWRVLLVCSESFLREGWSM
FTFSSAIYAQSPANPARIV-ALVHRDCLPLLPMELFGCINEDNILYV--------SEWA--
----
>Bgl-TLRγ13
FFPQ-SSLISLNISNNILGEYFALGRKKIFLGLGYLRFLDISMNLIYKLPRDFLSGLKSL
EVLLATKNRLQALNV--LSQMSSVWFMNFSQNSITWIDKVTRDDLDLLASLDISFNPLPC
TCDGIEILNWLAFT--NVRLVNQMYMKCQTSTG-ETVSLGDLRAQQVQACASAIILVISI
SSTVVVTLMVSLATLYRFRWKLRYLRNIALTKY-GFRPKKTGKKFQHDAYILYEDQTIKF
VFRDFIQELEVKRGHRLLLVDRDIMPGTIMTTAILSAVQNSYKTIPVVTPYFFDVWYSEY
AVQMAIMEEHYEPRQILHLCLYQATDPKDMPKDLLSVMKRNRYTEFPPETEMVKQFW
DQLSSTIQQE--
>Bgl-TLRγ2
FFDSYPALELLALESCRIDGLLSQHSFRVFQNLHSLQSLDLSFNSLDMLSPQTFSTNPN
LTSLNLAGNRFRNVP-FDIKLTPNVKFLDIRQNALTTIDISSRKALDELNRLLLSGNILSC
GCENLLLLQWLQET--RVELDGNRNFTCMNIKG--
LSSTLAYNLDGLWECWGFFNLSMAL
LCFTLLAYILFFTWIKNKTVILSSILQIFT-DFKKKP--S---DYQSGVYLGYAESEYKF
PCSELRQYIEDELCLNTFIRDRDLLPSLDIAQGVMDAINSSWRILLVINERFLHQDWFLF
TIRAAIYSISPANPSRVV-VLVEKNKVHSVPTELLSSVPNENIIVVSQ------------
-----Q---
>Bgl-TLRγ9
FFDGFSGLETLALSKCLIQRDFAFHSHRLFQNLKELRQLGLSFNSLNAFSNATFSFNSN
L
QFLNLSDNQFNYLP-FNLKHTPELRVFDVTNNSIITINVDARHELDRLARLFLRGNILSC
GCSDLLFLQWLKNT--LVELDQGGNFSCIDKDG-ERSYTLCHDLESLWPCWGFLSIAVII
VCLYVIVFFIVFLYIKRKTFIITYFLQLLG-HFHRSR--Q---DYKIDVFLGYSDTDYRF
PCQDLRAYLEDTLKLTTFLNDRDLLATLNKASGIVEAINSSWRVLLVCSEGFLKDEWSL
F
TMRSAMYAQSPANPGRVV-VMVHQRCLRLLPTELLSAVEEDNILVV--------SEWK--

>Bgl-TLRγ15
FFHNFPNLKKLMLGNNKLETYFNLPNYTLFSKLKKLKTLDLSDNAISKMPTDILAGLTSL
KVLYFEHNTLWTFNL--LSHMMNLRYVYLRHSQVNSLSEDVRQHIDSIGRFDLSFNPIHC
DCENYDFLKWMMNS--RAFDPKFTNYMCQYPDSSYK-NITDYTLRILRKCTDSFIFLFVL
AATFVMIAFVLAGIIYRFRWKLRYIYYATYLRLKSVDEE-NSEQFRYDVFISYAHQDEEF
ILKVLYPELGSR-GLNVHVHGRDFVAGEFIASNIVTAVRESRKTLVVLTLDLLKSKWCNY
EIQMANMESVHTGRQVLVFLLKDSLNNKQLGTELLFHIRNNTYIVYPQNDEELAVFWDK
LYKDLRK---
>Bgl-TLRγ17
FFSCLNSLRNLTLSVNMLGDFIGSSKERLFENLSSLSYLDLSFNSIDKMQVYFFHGLSN
VTEIDLSRNKISEFNV--ITKMNQLRRLNLSDNKISRLFSNVTDQIDRIKQVDLSKNPIDC
TCANLEFLKWMVNW---VNVSQSQGYLCKQDDGSI--AMPDYTVLSLNQCASVVIFLIII

GATLVLACVIVGMIIYRFRWSLRYWYHVAYLNYQQKRKSDRRQKFEYDVFISYVHNDE
TFVAQTLSTELEKR-
HVKVYMHGQKFVAGNYIASNIVQAVKSCRKTLVVLTNKYVRSQWCYY
EVQMANMEAISAGRPVLVFLIKEKIPNHKLG-EILTFIKTNTYIPYPQEDRELKIFYDKL
ASDLL----
>Bgl-TLRγ11

HITRLQLLDFSRNSITWITESTRDDLDALAELDLTFNPLPC
TCSGIEFIKWLATT--KVKLIDQVNLRCRLKDG-GSTSVGDLMLLFLQSCISSWILSVSI
LSAVFMAVVLGLVLMYRYRWKLRYLRNVAIAKF-
GFEPKKHQGLFKYDAFLVYDSDDMQFVLNECVQELEVRRGIKLCIGDRDFMPGTYVAS
DIVSAVQNSYRTVLLVTPEFYDDDYVEYAVNMAINEEIHTSRQVLYLCLYQPVALAEMP
RDLVAILKRNEFIEYPPEEGLIENFWDQLTAAVRQE--
>Bgl-TLRγ12
FFRP-NSLISLNISNNILGESFALDSGKVFSRLGYLRFLDISMNLLYRLPRGFLSGLKSL
EVLLATNNKLQALNL--LSHMSSVWLMNFSQNSITWIDKVTRDDLDFLASLDISFNPLPC
TCDGVEVLNWMAFT--NVRLVNQMYLKCQTNTG-EIVSFGDLRAEQVQACASAIVLVISI
SSAVVVTLMVTLALVYRFRWKLRYLRNIALAKY-GFKPKKTGKKFQHDAYILYEDQTINF
VFNDFIQELEVKRGHRLLLVDRDIMPGTYMTTAILSAVQNSYKTIPVVSPYFFDGLYSEY
AVKMAVMEEIYEPRPVLHLCLYQPTDHEGMSKDLLSIMQRNHYTEFPPDPELVKQFW
DQLSNVIQQD--
>Bgl-TLRγ4
FLDELYGLENLALSKCQFDRNFALKSARILQNITKLKVLDISNNSLNGLSKGTFSRNSEL
LYLSLSGNQFKDIP-FDLKFTPNLKILDLSSNIITTLTTDTTDALDLLNQLMLNGNILSC
GCHDLSFLQWLNST--LVSFDNNRNYTCMNKDG-
ERTNTLTFDLESLWQCWGFFYVAMITLCLYVTGAVLIFLMLKNKNFLVSYFLQIFG-
NFKHTR--S---DYKTDVYIGYSDEDYRF
PCIELREHLERNLKLSTFIIDRDLLASLDKASGIVDAINSCWRVLLVCSKSFLKDEWSIF
TMRSAMYAQSPANPAKIV-LMVHTSCLSLLPADLLSVVNDENILVV--------SEWK--

>Ttr-TLRγ4
FMSSFPNLKYLSLAYNNLGHMFDDVSKCVFLSLTELQTLDLSHNQIAKLPVDLFLNQHN
LKELILNHNKLKTMG---VASMASLQYMDLSYNEIRD--KSML---ESIATINLTKNAVSC
TCLNVVFLTWLINT--HINISGKETVYCLQQQK----MLIHFNFN---DCKKDYVIIGSV
VG-LVLVLIVIAVIVSNDR--VKYHIYLLKYKLR---NISRN-TEEDRIFISYCSEDRIW
VLRKLKPELEA-MGYKLFIHELDFEVGNFIADNIVHAIDTCFKTILVLSDNFVSSGWCMF
ELKMTLAK----S-DCAIPIYYKPVTKKNNTLLLKYLNKVKTYMKWPEDDREQYYFWQRL
KHALDKQED
>Ttr-TLRα5
-LTVMPQAP-LLLDNNNIEHLE--------------------------------YYLNDV
TKLILRHNAIADVPPNFVKLVTDMTLLDLSYNRIRYIDDDVLSSLKPTLSIAINHNPLAC
DCHSHSLKQWVSDHRK--RIVNLADITCFGG--AGGISILEAS--DLSICLD---IILPS
VIVPVVFICILILLVYIFRNEIKVILYKFNLHLN-----EEDETAVHDAFISYCSTDENW
VIKELANKLEMN--YKVCHHQKNFEPGVAIADNIVKSIDQSRRTILVLSNDFLNSDWCKY
EFQAAHYRALKNRQKYLIIVMLHKIDVSKLDNTLRLYVKTNGIIKVNE------LFWQKL
FYEMPIRTL

>Ttr-TLRβ3
LFEDLGLLKELVLKAADIANFRSKDIRAMLNVLIGLEHLNLEKVRLYSIPPTTFHHMHNL
SKLVLSDNFLSHLPEDLFFNLTNLKVLQLNHNRISQVSTKTFGFLDSLESIDLSGNPFAC
GCSLHWFLQWMNSTNVKVVGSQRFSYKCSSPPALRGKSLHEYYYKYRQNCLPIILVA
SVSGSCFLALLVSVICIVYRSRWYIRYLFYLLRARRKRQRKRNDEKDFAYDAFVCYNKD
DQDWVVRRLLPELEYNGEFKLCLHDRDFMPGIDIIDNIIESMEQSRRTILILSNSFAQSQ
WCQWELSMAQHKVLQDEGDILVLVLLEQIRSDNMSLKLHYLMRTKTYIEWTDNEDGR
KLFWEKLKGTLKAKPE
>Ttr-TLRδ
SLSGMEKLTTICLASNCLGSVP----PEIM-SARNLKQLDMSYNKISSIPP--IGGLKEL
RYLNMKSNRLRQLPN--LCQLKHLEIVCFSENTISDPNVDELLDMSKIKMLCLHSNRIPA
N---K-VQNLLKKASRDIRLEN-----C----------VEETHVKYLKKCL---------
-------------------------------------AMDENAVHKWDVLILHDDKDEEI
IENEIRPKLEEEMDFRVCIPYRDETMGMSKVAERSNLINFSKTIMLVITEKFNSSK--IL
GLDEVMLNGLDSETKCLIPVLWSKV---QVPKELKGRTMVRR----DS--VQEKYFWQKI
RKAIQSH--
>Ttr-TLRα2
-LNKIPKIS-IDLSGNNIPLIRHSHIDGSFTNMSNLLLLYLNNNNLKVLSRYTFEALPVL
EELYLHGNKLTFIEDETFLGLKKLRIISLKSNLIKTLPYTDF---SHLTSVSLAENPYDC
DCNFSRFKSWIFSSLA--TVIDSNDVFCVFYGLFPGSRLFNF---DLNYCELSSMIAIII
ILIVFVVIVALATVAYYYRNLIKVWLYNYGLRPR-----PDDSDKIYDAFVSYSSFDEST
VVHTLAPKLETNPKYKLCLHYRDFPIGSSIAETIVESVENSKRVIMLLSENYLSSEWCIY
EFKTAHHQVLKDRTNRLIVILYDEINMDNLDPDLRLYLKTNTYLCWKD------WFWQKL
YYAMPDVSD
>Ttr-TLRα3
-LTSLPFTEDLDLQNNSIRELT--------------------------------PYLKHV
KILNIANNKLEIVSAEAIQSLKTVQKFNLSGNRLTKL--NVNHFKTNLETLDIQDNQFTC
NCEDQWFQEWLLQINN--AVVNADSVRCHNK----DVAILSAS--HTDCGLANHTILTIC
VSVGAVMVCVAVVMVYIFRKEIKVLINHFSWHPR--RENDDNRHYLYDAFISYNLLNLDF
VRNSLIKNLE--PRYQLCIHNRDFLLGNEIADNIVTSINASKRFIAVVSKAFIESEWCQY
EFQFAHNDAMKDKRNNIIIILMEDSDLGEIDNCLKIYLRTHTYLSYKD------LFLQKL
LYSMPQVRT
>Ttr-TLRγ3
LMSSFPNILYLSLANNKLGELQEKQFKDVFYPLTKLEEINLSGNNITYFPVNVFLAQTKL
KRLLLHDNSLKVWH-INMSTMTSLEYLDLSENQITIIGQMSMNYFKTIVSINLNDNKIDC
LCFNLEMITWIQK-S--KFIHQRDNLKC--GDTK--KSILSYDPK---SCEVAELVIGVT
LGISALFIGVLFLIFYKIHW-LKYKLHLLKWRWRGMMA--DQNIEQEMIFISYENRDRCW
VINTLLPKLEG-MNYKTYIHNRDFTVGRPIADNIVHAIDICARTVLILSDHFAQSEWCVF
ELNMALV------KNSVVPIQYAP---
>Ttr-TLRα1
-VEDVP----------------------------SLEELSLQFCNFSVITRNMLQNYPNL
KTMYMHNNRINYIETAALTRKVRINVITLDNNRLTHID-------KQVIIVRLQGNPWDC
QCHLKPLSDYVRNHIK-------GNITCYSPPS-----LASTPLQQVNTCEQGLLFLPIM
LGLLLALVLASTCCVYWYRYEIKIMWNKY-------RKAYKTEKHTYHAFVSHSSVDFKF
VKDNLLVSLE--PTYKLYVYYRDSIPGSTIVEDIVKAIDDSAITIILLSQNFLHSDWTKL
EFKQSYFKAMKSKSNNMIIILMEDIPLDSIKPQIKAYIRTKTYIHKND------RFFEKL

TSSMPKEEM
>Ttr-TLRγ2
---NDISYFPDNIFIHQTKL
KKLILRRNAFQVWN-VNMSTMLSLRYLDLSKNLLTVIGETSLTFMDTFMTINLEDNLFIC
SCPYLPTISWIQEN--NKSIRQAQNLKCKMGEN--EIKLMSYNAE---SCHVIYDIIGIT
SSISVIIVMATIFISYKFHW-IKYKFHIIKWRLRNCFGIHDQPANNERIFISYENRDRRW
VLDTLVPKLENTMNYNTCIHAWDFMPGYPIADNIVRAIDICTKTIVVLSDHFAESNWCQL
ELQMALV------KHSVIPIRYAPIEKQNKTRLLKYLTKANVYIDWYDMHNKEDAFWDKL
KYTLDRDDE
>Ttr-TLRγ1
---NDISYFPDNIFIHQTKL
KKLILRRNAFQVWN-VNMSTMLSLRYLDLSKNLLTVIGETSLTFMDTFMTINLEDNLFIC
SCPYLPTISWIQEN--NKSIRQAQNLKCKMGEN--EIKLMSYNAE---SCHVIYDIIGIT
SSISVIIVMATIFISYKFHW-IKYKFHIIKWRLRNCFGIHDQPANNERIFISYENRDRRW
VLDTLVPKLENTMNYNTCIHARDFMPGYLIADNIVRAIDICTKTIVVLSDHFAESNWCQF
ELQMALV------KDSVIPIRYAPIEKQNKTRLLKYLAKANVYIDRYNMHNKEDAF----

>Ttr-TLRβ5
--LTHLIVTGNKLTTLSPELF---
THLKYLDVSNNSITSFSREIVGDLGYIERFIFDDNKIECDCELSHFQQWLLTTLI----
DTSKTERCYN---YEGVRIIDYQPTWID-CDNTYVVVGSI
GS-
FCLLVATVAALLVYYRWDVKYWFILRKIKAKRYHNMHDENNVMYDAFVSYSSLDEGW
IYNELIPNIEDDIKFQLLMDQRDFLPGHYIIENIVQGIDSSHKVLLIISLNFIESQWCTF
ETRFAEQSSIETG-QRLILIFLEPLKKSEMSRHLQRL----------------------------S---
>Ttr-TLRα4
-LNALPYVP-LYLQDNHITHLT--------------------------------DYLALI
TELNLDHNAISEIPLAFLNSIPKMKTLKLAYNQIKYFPEEIEETR--AFNWSMHHNPIAC
NCYSLWLKKWVSANRK--RIDNLHDIVCFSG--AGGVAILEAS--DHLICID---IILAA
TITPASIIIIMVLLGCIFRKELKVILYKFNWHPK-----RENESLPFDAFVSYCSADEHW
IVTQLAKKLESNPPYKLCLHYKSFEPGVAIADNIVTSIDNSKRTILVLSDKFLESEWCRY
EFQAAHYRALKNRRKYLIIIMLNKIDPSKLDKNLRLYLKTNGYIKPTE------LFWEKL
KYELPMKSS
>Ttr-TLRβ2
IFMNLTQLQWLVANHNNIGMCLTKGMSKLFQNLKSLQWLDLSSNQIETLPKELFQNLK
SLKYLNLSSNRISYWASEQFTALKKLQTLDFNSNVITTINKSSIGQLENV-
HLNLSNNLFSCDCDLRWFRNYINYT--KIDFTYIKDYLCAAPPDFQGKHFLKFHSNMII-
CSPYLIRYISIGGAVVLIVLMISLATYNWRWYLKLKLFRLKNTLRGFQ--
EDDDIVTYDAYLSFAEEDRDWVTRTLLPKIDNEGRYRIYYDDRDDMPGDNIINAIDSGIE
KSEKSIVVFSKKYATNGRIDVDLTLI----
LDKPHQRVILIMLEEVPLRMIPRCLHSTLWSNQHLLWTEDVNGQALFWEKLNNKLMED
--
>Ttr-TLRβ4
------------MGDNSLYR--PNDLPALFAPLHSLTYLSISKNKLDYLHEDTFNGLYNL
EKLILTTNKLEYLSTDLFKNTTKLKTLYLAKNSLKTINAGTFEKLTFLKDINLGENQFDC
HCDIRPLRDWLKYKQKKKAIKIQGDLNCTTPPNLRNSLIVDYNPSWLD-CDNEYLLISSS

C--SMGFVLITITVIYIFHWNIKLFFAIRKANRKIDGENNPLLRKRYHAFISYANDSLWW
IKKHLLPNLQDNFEFNLCIRDRDFRAGQAEVDNIIDGMQNSTCTIFLITAEFIDSGWRQF
EMNVILRGLIDDPNNRFILVFLEDIPNNKLPIVLSTLKKNVDCLYWP--KVKRIQFWAKL
KVRILGK--
>Ttr-TLRβ1
IFFNISTLQILNLNKNLLSELP----DVLFTNLENLQCLDLSSNLLEVIPEKLFANLKSL
TDLNLANNMLYNTN-GIFHPLIHLTFLNLSSNSLTMITKDTLAGPKKLKTVDLNKNVFKC
TCDLQWFVDRLRQSKQCPYIVQLREYKCTN---LPGTCVANFMPSQWE-CHSIFIVVISV
LGS-ICITLLLMGCCYRYRFYLLHFCFVLKRLRETYEELYDNTQYRFDAFICYNDEDLNW
VQSQLLPKLRE-ATIKICINFMHFRIGAPRIDTIMEGIQTSRKTVLVISRHFLDDDWCLF
EMNVAAHRLFEEGKDNLVIIFLEPIQYSEMPLTLQAVVRTKRYLEWSTNEQGKDLFWET
LCYLLKTRPS
>Hps-TLRγ4
FMSSFPNLAHLSLAKNKL----KK---NVFWPLKLLEYLDLSDNQISMLPKGVFSQQSSL
KYLILKDNALTKLS-LGLKNMKCLKYVDVSVNKLETLEPQTRSFLEKKMFIFMEDNVFQC
SCSNIDMLYWMRNMNLKSSVQRWSQVKCHNYEN---VNLTDYDIS---KCDSIRTLVVTL
IGVLV-LMLVMGVVIYKSDR-LRYKWHLLKWRLRNNR---D-HRQNFKIFFSYGSRDRQW
VWEVLKPKLEQ-DGYSLFIHEIDFHVGECIADNIVYAIDVCDQIVFVLSDNFVSSEWCMF
ELNMALV------KHCIVPIRLSPIMKRN--RLIKYLTKTRTYLEW-KDKESADEFWARL
YSRLNRK--
>Hps-TLRγ3
YLSSLPNLAYLSLAKNKL----NI---DVFTPLKLLEYLDLSGNQIAILPKNVFSQQDNL
KYLIMKNNALKTLN-
FQLKNMNSLEYIDASENKLGTLGQQTRFFLEMMMSISLEDNVFQC
SCSNVDMINWMTKTSLKVSRQRWSQIECFNLRS---VNLTDYDIS---KCDSVTTIIATI
VGVLAPAMFCMGLVIYNYDR-IRYKWHLLKWRIRNYQAVVR-
HRERFQIFLSYDSCDRQWVWKVLKPKLER-
EGYSLFIHEIDFHVGECIADNIVYAIDVCDQIVFVLSDNFVSSEWCMFELNMALV------
KHCIVPIRLSPIMKRN--RLIKYLTKTRTYLEW-KDKQSADEFWARLYSRLNRK--
>Hps-TLRδ
DFSNLEKLRTVILLCNRLEKFP----TSLL-DVKSLAQLELANNRIREIPP--IGQLREI
KFLSVKCNRLTSLPE--LAKLEVAEVICFSENMIVDVPVESLLRFKNLKTLCLHSNRIQN
H---K-VMQLHE----DVRLEN-----C----------IGD-DIQ---KCR---------
----------F---AYHVR--------SSNFRL-KCRNM-DKTTWKWDVYIAYAEAAEHI
VDEELVPKLTN-MGLTACVYYKDSQPGKDIMADRRDMIDRSKKILVLLTKDTSYSDF-IS
EIQHIVSEGPKDQTARLIPVQWDE---AIIPDELKKVVVTSR----RT--AQEKVFWSRI
EKALKS---
>Hps-TLRα
-LTSLPQVP-LYLSNNRITELS------------------------------SYLGSL
TKLHLDHNSLREINPNFLSQLKNLTFLSITWNNIKYFPESIKGT---LFNLSIHHNPIAC
DCHSLWLKKWISRSRN--RFDNLKDIVCVDG--AGGSPVLEAQ--DNQICL---KMILLG
TIVPFVVIIIIVMLVFIFRKELKVILYKFHWHPR-----REDYTLPYDAFVSYSSGDEHW
VVSQLTKKLEGSRPFKLCLHYKSFEPGVAIADNIVTSIDSSRRTILVLSNNFLNSEWCKY
EFQAAHYRALKNRKKYLIIMLNEVNTDKLDKNLKLYLKTNGYIKPSE------LFWEKL
QYEMPVLEP
>Hps-TLRγ1

ATSAFPDLTYFSLAKNKLEMMFNINSTDVFHPLEKLQFLDLSENGIIDVPSNVVEKQVSL
RMLNLSNNNMQTFKV--LFSLRNLTYLDISNNLLKTIDVISTIGLDQILRINMDKNDFEC
VCSNVRTIDWIRKDSLRSTILRRDDLQCKVIKEGGWNKIVDY---QLNDCDTDLIVLPIA
SA--LAVITFIFGIIFWKRNQIKYKLHLLKWRW-GFLKT-NPHVERGQIFISYDHRDGDW
VRNTLRPNIQE-MGYRPYLHEIDFVPGESIADNIVHAIDVCDKTVVIISDYYAESQWCQF
ELQMAITKGL----GYVIPIKYAKLKRK--NKLFQYFMKCVTYLEWPADDDTRAKFWIRL
GRAIAKE--
>Hps-TLRγ2
YLSSLPNLAHLSLAKNKL----NI--TDVFTPLKLLEYLDLSGNQIAILPKNVFSQQDNL
KYLIMKNNALKTLN-
FQLKNMNSLEYIDASENKLGTLGQQTRFFLEMMMSISLEDNVFQC
SCSNVDMINWMTKTSLKVSRQRWSQIECFNLRS---VNLTDYDIS---KCDSVTTIIATI
VGVLAPAMFCMGLVIYNYDR-IRYKWHLLKWRIRNYQAVVR-
HRERFQIFLSYDSCDRQWVWKVLKPKLER-
EGYSLFIHEIDFHVGECIADNIVHAIDTCDQIIIVLSDNFASSEWCMF
ELHMALV------KHCIVPIRLSPIVEHNN-RLITFLTKTRTYLEW-KNKPSGEIFWARL
YGTLNRE--
>Lan-TLRδ7
EVENF-ELRSLCLACNVIDNLP----GKFF--MKQLQELDVSYNRLTQIPA--IRNLKNL
EFLRLTGNRLQTIPS--IESLDRLLYLCLSENALVDIPTNALHRLINIKSLCLNSNRLPC
E---V-VIQVIQESSPKVSLREN----C------K--EIRD-------------------
--------------AFRAK---------------QYFEK-DGKDFESDVYVMHADEDYAL
VDQEIVPHLEER-NLKVTVNIQALRPGLPVSDQLVHFIESSRKILVVFTKNDVFEHTCLT
KVKAALEKRKKES-ETSPIVLVE-CPKSKVPHEFKDLYVIHR----RT--THEKHFWPNI
INAITQ---
>Lana17091
-LIHMSKID-LDVSNNYLETIP--------------------------------SD-TMY
REVYLDNNSISTFPTSQV--LPHLTTLSLRYNSIRTISMRQIEKDT-VNDLYLGGNPWRC
DCHARSIKHWLLNNSN--IIRDLDDITCVSGELTLGKSIKNVP--DNNGCPI---IAAIV
GGLVFVVIIACMLLLYKCNLKVRIWLYKFRFRFK--D--KQDSDKIYDAFISYSSLDEKY
VVQTLVPGLENTPPFKVCVHYKHFIPGASIAESIVEAVENSKRTIMLLSQNFIHSEWCTY
EFKTAHHQVLKDRSNHLIVVVLGDIP-SDLDSDLKLYLSTNTYLRADD------WFWEKL
LYAMPKLEN
> Lan-TLRδ1
DFKC-LKLKALYLNANYIRELT----PNIL-KLEELIIFDGSYNELQVLPN--IDQLQSL
KYIRLKQNQLRRLPE--LGNVKSLEVICVSENRLQDIPAEKLAKLPKL-RLCLHSNRLGQ
----K-VVRTLKEAKFEVRFDN-RSVD---P------KISKI---KVEGCH---------
------MTVLT------------------------------RETPVFDVFMLYSEEDEKV
ITDEFLPKLEKKAELKVCFASRDYIPGHFELKEALTNMRKSRKIIALLTEHFDEQK--AV
EINHAVDADLARQSCSVIPVVWGNV---KMPVQFKRIVPLRR------------VDWDRL
ITAIKE---
>Lan-TLRα2
-ITTLPAMP-IYLQNNKLEIIT--------------------------------DYFSRV
HTLVASNNSIAKITSRIFWY---ISHVQLDGNNLKSLPQDIESMKHNITSLSLSRNPWTC
SCENLWLKSWLLKKRK--VI-HMDSVICTNE--VKGKPISQVT--EEMLCHPSYIQVAVS
LGVLLLLTLITIAVLYKYRFEVKVILHRFNWHPR-----QEMTEKLYDAFISYSSEDRLW

VHTTLAPTLENQLPYRLCMHCRDFLPGEAIDNNIIQAIQNSRCTILVLTKNFLRSNWCIF
EFQQAHYQMIHNAHFKVIVILKEDIPAEEMDDDLRAYLRTHTYLEAKD------WFWKKL
LYVMPTMNK

>Lan-TLRβ6

---------------------------MFRNLTQLRKLYIQNTGLSFLPPNVFVNNGMM
SELQLQSNFLSTWDPIVFQPLLSLRKLFMDHNNIRILNETSFFIWDNLTDLNLAGNPFSC
TCENLWFRNWIQST--NVKLLQLHAYLCYEPKKLSKSPFLDWHPTKAQ-CTPAWVIASAI
GVPTLLFLALVIVVSHRYRWYIRYWCFTLRSRYKRLEPFEDNGTYVFDAFVSYNCHDR
PWVIQRLLPKLEYDAGFKLCLHDRDFIVGHDIVDNIVDGIDVSRKTILVLSNNFAQSQWC
QLELTMAQHKLFDENKDILVLILLEDIKPENLSNRLTLLLRKQTYIEWPSEEEGQDLFWE
RVKAALQKPSG

>Lan-TLRβ9

IFNNVPTLTELCLDNNQFYRILLDILRDLLRPLRHLRCLSLTANKLTELPLGMFDGLANL
TTLDLNLNSLRSLPVEIFRHQRQMTDLHLDRNSIFTLSGHMFANLTALKNFNYARNKIIC
DCNIRSFQSWLATT--SVNV---PRELCFGPEWAQKTPIKEFRPSWFA-CDD--VYLAAI
SGGCVFFVIFLSAVLYSFRWDILYIYAIYRASGKKSKLGRREPHKTYDAIAMYSPTSVTW
IKKHLIPNLEEDIRFKLCINDRDYIVGDPLVDNVETNMEKSRRILFLLTREYFESQLHET
EINLAQVKLFDGFVDKIIFVFLEEVPKTTFKEPLKTMMRHGNCLHWPRKKRERTIFWKR
LKLALLEAKT

>Lan-TLRβ8

IFKNVPTLTELYLDNNQFYRILPDILKDLFRPLRQLRHFSLG------LPP---------
-----------------------------HQSMYRVPGNFA-----L---DPNGHK---
-RQLKSFHGSLA----MTTV-------CIWPPYREDA------------CF---------
------FVIFLSAVLYSFRWDILYIYAIYRASGKKSKLGGREPHKTYDAIAMYSPTSVTW
IKKHLIPNLEEDIRFKLCINDRDYIVGDPLVDNVETNMEKSRRILFLLTREYFESQLHET
EINLAQVKLFDGFVDKIIFVFLEKVPKKTFKEPLKTMLRHGTCLQWPRKKRERTIFWKRL
KLALLEAKT

>Lan-TLRα4

FLTSLPNFPNLEVQRNQL---------------------------ERFPN---IALPNL
WILSLKDNSITEIKNESLQHVPNLRYLSLEGNGITHIPEGFFNHTPHM-TANLTGNPIKC
DCSQRWIKDWMLQQEKRTIF---VEAFCSNSSG--AINIKDF---DFEACVPTFYVTLLV
VLVLLIIVAILILLIYICRKELQVWIIRGWWKA-NVLTNSAQRTYKYDAYIAYCDNNYSI
IRDHFIPRLEQKHGYRLFIRDRDSEAGQPIAENVANAISKSYCTIALLSNSAMESEWFPV
EFELTHSLSVEDKSRRLVIVKVGHLSKEALQKSIQLYLTTKTYLSWTD------DFWDKM
HKILPDKRE

>Lan-TLRα3

-LTGFPSLP-LYVNRNQVETFP-------------------------------TLFSDL
QELQAADNNIGSISNSSFTAFPKLQYINLDRNGIAEVVVGTFDSL--LSMVSLKGNSLHC
DCSQRWIQDWIIRNLT--FI-----ATCNDT--------------DFTQCDT-NVVALAV
VLVVLAVFIALVAITFFYRTEVEVLIYRYKQ--R-----DSDTDKDYDIFISYSNDDSVF
VRNVIIQKMETEWGYKLCIHERDFLPGEYIADNIANAVEKSRRTLTLLSDSYLHSEWCVF
EFAMAHQQSLKDRCRRLVVVKLSDLDSNLLAKEVGIYLKTNTFLHKGC------MFWEKV
RGTLPAKPL

>Lan-TLRα8

-LTALPFAP-LEMAGNLIEVLE-------------------------------PYLANA
TKLILSNNAIQTIDPAVFGLFAELRTLHLDGNHLTHLPKEITSVN--ISEIKLDKNYLSC

DCKSTWLKRWLNENGK--NIPRFTELTCAVG--QNGQRIIDVP--DSSTCDPLIVPIAIC
LAVVLVILAVNLIV-YRFSIEIKVLVYKFNWHPR--D--DDGPEKIFDAFVSYSSQDYKW
VVHNLRHTMENVPPYRLCVHDRDFIVGETIFDNIMNSVQQSKRMIMVLSQNYVDSEWC
MMEFRTAHQKVLKERSKYLIIILFDDVNKDQLDEELLAYLNTSTYLEVSS------WFWKKL
FYAMPDLSK
>Lan-TLRβ5
VFSHAPHLQELYMSDNHLDKIDDAALEKLFRNLTKLRKLIISQTRLTHLPPKLFETKPFL
RELQLGSNQLSSLDPVVFQSLFSLQMLYLENNLIRTIYESSLFVWKNLTKISLAENLFSC
TCDNFWFRTWMDTT--QTTIVALNSYRCYEPKELAKSPFLDWHPSKAQ-
CTPAWVIASAIGVSIMLFLALVTVVSHRYRWYIRYWCFTLRSRYKRLEPFENNGTFVFD
AFVSYNCHDRHWVIQRLLPKLEYDAGFKLCLHDRDFIVGHDIVDNIVDALEVSRKTILVL
SNNFAQSQWCQLEMTMAQHKLFDENKDILVLILLEDIKPENLSNRLTLLLRKQTYIEWP
REEEGQDLFWERVKAALQKPYG
>Lan-TLRα1
KLNGQPNMEIVEFSNYAY------VFITIFHGYPALHTLIAVRNNITVFPQATLLNFPKL
RYVDLRYNSIKELKI---PR-GSNRVFDLRHNDIQDLTIENVNAMRYAAHVDFRNNPIDC
GCNNSDAVKHLRSEVVKSTYRFLYDIPCHHGET--TTTIRSINLDDLNECF-IIIIPWIV
LGLLACLVVLTAILTVYFRREIQILLFRLKCRCR------S-PPKRFDAFVSYNSGDEHW
IVHTLAPKLENKPPFRLCLHYRDFIVGAAIAENIIESIEASRHTIMVLSENFLKSEWCLM
EFRAAYHQGLRERNKHLIAIVLEDILLDDIEADLRSHLRTTTYLKVSD------WFWDKL
IYCLSRNPH
>Lan-TLRα6
-ITTLPAMP-IYLQNNKLEIIT-----------------------------------DYFSRV
HTLVASNNSIAKITSRIFWY---ISHVQLDGNILKSLPQDIESMKHNITSLSLSRNPWTC
SCENLWLKSWLLKKRK--VI-HMDSIICTNE--VKGKPISQVT--EEMLCHPSYIQVAVS
LGILLLLTLITIAVLYKYRFEVKVILHRFNWHPR-----QEMTEKLYDAFISYSSEDRFW
VHTTLAPTLENQLPYRLCMHCRDFLPGEAIDNNIIQAIQNSRCTILVLTKNFLKSNWCIF
EFQQAHYQMIHNAHFKVIVILKEDIPAEEMDDDLRAYLRTHTYLEAKD------WFWKKL
LYVMPTMNK
>Lan-TLRβ7
IFSAVPSLNTLSLANNSLGQT-
PAILKKMFKNLGQIWKLRLSGNGLVELPLGMFDDLVQM
TDLHLQVNQITTLPAGIFNKCKKLAHVNVENNKIISISEGVLWAIGSLRQLDLSGNKWTC
DCDIRWFVHWLRNT--RVLLSKGKQEHCNLPSDLRQLKLVDFCPAWIE-
CDNLHLTAGLTSS--VVAITLTSYLIFIFRWDIKYAWVIRKTRRNGYVEI---
PDERYAAFVSYCSKNTKWIKDELLKNVEDDMGLRLCIYERDFICGNPIVDNIEEYMNQT
TRVVFVVTGDSLQSRLCDHEFKVAQNKLFEKRITSIIFILHEDVDKKTIPDNMQTMMRHT
TCLCWPENGRQKTVFWKKIRLALLR---
> Lan-TLRδ3
DFKD-LKLKELYLNGNKIRTLP----PNIF-KLRELTHFDGSYNELQSIPD--IDQLQNL
KYIRLKQNRLRRLPE--LGNVKSLEVICVSENCLQDIPAEKLAKLPKL-RLCLHSNRLGQ
----A-VFQTLKKAKFHVRFDN-RLVD---P------KIKD----KIEGCH---------
------MTVPS-------------------------------GKMPIYDVFILYSEEDEKL
INDVFLPGLEEENELKVCVAFRDYIPGQYVSEEAISNMKKSRKIIALLTEHFDEQK--AV
EINQAVGADQGRQSCSVIPVVSGNI---KIPAQFEKIVPLRA------------VDWDKL
LTAIKA---

> Lan-TLRδ4
AIKKLTKLESLALNANEIRELN----IGIF-DLEHLIFLDASHNPIFAIPK--VQKLKKL
EYLRLKMCRLQALPE--LGDLPRLETICVSENMISKVPAEKFQKMRQLRTICLHSNRLSV
E---LKLRQ-LK----DVRLLNDQSLNC--------------------GCY---------
---------------------------------------K-KPTEKDVLIIYSGTDDRV
VDDEILPILEEELRFSAVVDFRDFIVGKPVFTQYAANRKSCRKILFVLTADFCSGK--RM
HLNEALQAVADDKRSRIIPLIWND-PNFQLPEELRSYVQLHK----NE------SRNEKL
WKALA----
> Lan-TLRδ2
DFKG-SQLKALYLNANYIRTLP----QNIL-KLKELIIFDGSYNELQFLPD--IDQLQNL
KYIRLKQNQLRRLPE--LGNMKSIEVICVSENRLQHIPAEKLAKLPKL-RLCLHSNRLGQ
----A-VVQILKTAKFEVRFDN-RSVD----P------KIPKI---KIEGCH---------
------MTVLS------------------------------RETPVFDVFILYSEEDEKL
ITDKFLPQLEDKAELKVCFASRDYIPGHFELKEALNNMKKSRKIIALLTEHFDEQK--AV
EINQAVDADLARQSCSVIPVVWGNV---KMPAQFKKIVPLRR------------VDWDRL
LTAIKE---
>Lan-TLRβ1
LFKGTSKLKILFLSDNELGYVFNDPNGMLFKNLYKLENLTLERNRISQLWPAQFQNLTS
VKNLSLSDNQVSFFTSQLFAPMTSLRALNLSQNMISLVNSSSIGGLGRLQTLDLSGSPF
ACTCDLVWFRRWINQT--NITLSQLDVYTCNTPAERRGMPLLQFDPDAID-
CVNPIYLASAVGG-TLALLVVVFISLYRWRWFLKLRYYRFKRLAKGYERV-
EGDDIVSDAFVSFCDADREWVAVELLARMDSAGRFNL-
VCDLNFLPDKSELESVVEAIECTRKAIVVLSDAYIGDPRCQF
ELEQIYESSVERQRYEMILVLKG-
LPNGKIPKVLRQRLERGEFLEWTEDANGQQLFWDQLGEKLEQRPH
>Lan-TLRβ10
DIRNLTRLKILHFCDNTISLTRPD--NNFFTGMVSLESLNLAGKELEGVDLTLFNPLINL
KVLNMTYTGLTKVTPGTFKPLQNLRTLDLSDNKLVEIDGDIFKYIPKLATFLFNNNRFSC
DCHLVRFVGWLKHT--SIQI---EDQPCFSPSKLSAVKVGDYSPGFLE-CK-QVLLYALG
--TLVL--LIFTAVITFYRWDIRFWWQKVRPKKQGYIPI----DGEFDAFVSYSSKDEDW
VVGTLVRNLEEEARFQLCLDNRDLIPGNFIIDNLIQGMEKSKCCLFVITRNFVKSEWCNF
ELNTAISKMLDERKNVVILIYLEHIPDKDLPKNLRRLKKHVTHLKWPNDERKIDIFWKKL
QLVLYHKKE
>Lan-TLRδ5
NIKKLTKLESLVLNANEIRELN----IGIF-DLEHLIFLDASHNPISAIPK--VQKLKKL
EYLRLKMCRLQALPE--LGDLPRLETICVSENMISKVPAEKFQKMGQLRTICLHSNRLSV
E---LKLRQ-LK----DVRLLNDQSLNC--------------------GCY---------
---------------------------------------K-KPMENDVLIIYSGTDDTV
VDDEILPILEKELRFSAVVDFRDFIVGKPVFTQYADNRKSCRKILFVLTADFCSGK--RM
HLNEALLAVADDRRSRIIPLIWND-PNFQLPEELRIYAKLNR----KD------YFWKKL
QKALA----
>Lan-TLRβ4
IFSNSPNLHDLSMHDNQLNDMNSTALETVFRNLTKLRKLYLQNSKLANLPAKMFVNNG
MLSTLQLQSNYLSTWDPIVFLPLLSLKHLFMDHNHIRILNETSFFIWTNLTEVNLAGNPF
SCTCENLWFRNWIQST--KAKVLQLHKYICYS----AKTPFLDWHPTKAQ-CTPAWVIASAI

GVSIMLFLALVIVVSHRYRWYIRYWCFTLRSRYKRLEPFEDNGAFVFDAFVSYNCHDR
HWVIQRLLPKLEYDAGFKLCLHDRDFIVGHDIVDNIVDALEVSRKAILVLSNNFAQSQW
CQLEMTMAQHKLFDENKDILVLILLEDIKPENLSNRLTLLLRKQTYIEWPREEEGQELFW
ERVKASLQIHSG
>Lan-TLRα5
-YTSVP-LG-LTLANNGITELKNNSINQSFTGLFQLRTLNLSFNLLEDLKEYSFSGMTML
ENLYLDHNLLTSIDPSTFASLSRLKILTLHSNRLEYLLPDVF---TSLVHLTLSHNRWPC
DCDVIYFKHWVVSYKA--IIFDVGNINCTFKRIVQGKRVLYF---DEDYCNRTHVAALVS
VSILFFLTVIVTSLLLYYRTEIKVWIFKFGCRPK-----PDDDEKIFDAFISYSSKDEHL
IVHELAPRLENHPSYKLCLHYRDFPVGASIAETIIDAVEASKRTILVLSQNFLDSEWCLY
EFQTAHHQALQDRTNRVIVILLEDIPLKNMDNELRAYMKTKTYLRWDD------WFWDKM
AYALPDVHK
>Lan-TLRβ2
LFSHTPNLHELNLNSNHLRHLNSTAMETMFRNLTQLRKMYIRNAGLSVLPPNMFVNKG
MLSELQLQSNSLSTWDPFVFQPLISLKKLYMNGNRISVLNETSFHIWNNVTEMDLSGN
PFSCTCGNLYFRNWMQTT--QVKLLEIHRYQCFEPKDLEKTLFLDWHPTIAQ-CT-
VWIIASAIGVSTMLFLALVIVVSHRYRWYLRYWCFSLRARYKRLEPFEDNGTYVFDAFV
SYNCHDRSWVIQRLLPKLEYDAGFKLCLHDRDFIVGHDIVDNIVDGID--------------------
---------------------------NLSNRLTLLLRKQTYIEWPSEEEGQELFWERV
KAALRRPPE
>Lan-TLRδ6
NIKKLTKLESLVLNANEICELN----IGIF-DLEHLIFLDASHNPISEIPK--VQKLKKL
EYLRLKMCRLQALPE--LGDLPRLETICVSENMISKVPAEKFQKMGQLRTICLHSNRLSV
E---LKLRQ-LK----DVRLLNDQSLNC---------------------GCY----------
---------------------------------------K-KPMENDVLVIYSGTDDRV
VRRRILPILEKKLGLSAVVDFRDFTIGKPVFTEYADKLKNCRKILFVLTADFCSGM--KL
HINEALQAVADDKRSRIIPLIWND-PNFQLPVELRSYAKLNR----KD------YFWKNL
KKALA----
>Lan-TLRβ3
IFSNSPNLHDRSMHNNQLNDMNSTALETMFRNLTKLRKLYIHNSKLANLPPKMFANNG
MLSTLQLQSNYLSTWDPIVFQPLLSLKKLFMDHNNIRILNETSFFIWTNLTEINLAGNPFS
CTCENLWLRNWIQST--KVKLLQLHYYQCYAPEKLAKTQFLDWHPTKAQ-
CTPAWVIASAIGVPTLLFLALVIVVSHRYRWYIRYWCFTLRSRYKRLEPFENNGTFVFD
AFVSYNCHDRHWVIQRLLPKLEYDAGFKLCLHDRDFIVGHDILDNIVDALDVSRKTILVL
SNNFAQSQWCQLEMTMAQHKLFDENKDILVLILLEDIKPENLSNRLTLLLRKQTYIKWP
CEEEGQELFWERVKAALQKPYG
>Llon-TLRα2
-LSAIPRMP-LHFENNRIEELS--------------------------------
GYLKFVVGLWMSRNDITAISREVVELLSKAKGIYFQYNKISRLSKSVMSLWKGVSKLDL
TYNLLVCDCHSEWLRHWIIEASY--LV-NGWKLRCASDETARGRAILTVE--SHEVCKT---
IIAIIFGTVFILLIVAFALVVRYRQEIKIWLYKYDWHPK--D--DSDPSLIYDAFICYSSLDYDW
AVHTLWNKLENTPPYKLLLHQRDFIPGQMTMDSIYEGVNSSKRMIMLVTQNFVRSDW
CMAEFRTAHHEVLSKNTNYLIAILGEDLDIECVPEDFKVFLKNTTYLKKDE------
NFWDRLFYALPQKGP
>Llon-TLRβ8
AFLGLEHIEELDLNDTDIGAV-REDLKYVFKPLKGLKRLNLADSLFKHYPVVIFQNQAEL

EELDWSSNAITAIGSVVFSTLRRLRRLDLRNNQLIYISGKVFTTLTNLQTLLMWENSFAC
NCKLRGFTYWLSRSKFKDAICESYSEPCRSPPKHVGHRLESFLPTWLD-
CENAIVALSTSLVLLFCLSVSLSVVAYRKRLSIRYWYVIRKLKRKGYIPL--
SRRHSYDVFIAYMPQEQRWVEHTFRPELEKDVAFRVATVDREFQVSGEVIDLVEGGF
RHSSHVIFIVTDEFLSWEKSDYMMTQAEVMYLEKGCPEPILVLKERITMDQAPLSYKRLI
RHVVRLHWPQGQGYTEDFWKNLRLVLLGERT

>LIon-TLRβ4

EMEHLTKLEKLFLSCAGTTVLP--------STLATLKQLEISEWSFREDSIQIISSLKNI
EILSLQQSNIHHFPYQELMMLDNLVTLDLSRNRISALDHKRIWKIPKLRKLNVASNNFEC
NCSMLPFSEWLRRPRPRIEIISLFNVKCASPSKYYNMALFNFDKD----CRSPIILPSTL
GP-IGLLIAIVIFVTVRYRGYIRYGLMLIRARWRGYGSI-EGCKFKWDAFVSFNGADYDW
VYNQLKPKLEDEAGYRICLHHRDFTIGEFITDNIVKCIDRSRKTLLILSDDFAKSQWCQL
ELSVAQHKLFYDDRDVLILVKLNDVSPENITGTMQVVMRTKTFITWSDALAEQDLFWK
QLILALKRPPG

>LIon-TLRβ7

AFTGLENLEQLELNDTDIGAV-IEDLKYMLRPLKSLKRLDLDKSRFSHIPEDTFLNQVNL
EELHLADNEIRVIGHSAFRTLVRLKYLDLRNNRIEFIHGEAMGHLTSLLTFLFTENNFGC
HCDLAGFTKWLKEHTFHENRCEWRSERCTVPLHLKDTPILDYQPGWTD-
CDNLILTVSSIFSVFLILSSSIAIHSYRRRLSIRYWYVLQKLRRAEGGTQ-
NSLDEPFDVFISFELNDRYWVEETLLPNLEDDIRFRVCTVDRDLDPGRPEVMNIARGIR
NSRNVIFVVTRELIQTAWCEYEICLAETQSLQEGNCRLIIIFLEKFTWEELPLCMKRLLSH
VNFLRWPETAHEQEDFWRRLRLVILGETT

>LIon-TLRβ6

AFTGLENLEQLELNDTDIGAV-IEDLKYMLRPLKSLKRLDLDKSRFSHIPEDTFLNQVNL
EELHLADNEIRVIGHSAFRTLVRLKYLDLRNNRIEFIHGEAMGHLTSLLTFLFTENNFGC
HCDLAGFTKWLKEHTFHENRCEWRSERCTVPLHLKDTPILDYQPGWTD-
CDNLILTVSSIFSVFLILSSSIAIHSYRRRLSIRYWYVLQKLRRAEGGTQ-
NSLDEPFDVFISFELNDRYWVEETLLPNLEDDIRFRVCTVDRDLDPGRPEVMNIARGIR
NSRNVIFVVTREFAQTSWCEYEISLAETQYLQEGNCRLVVLFLQKFTWEELPLCLKRLL
SHVNFLRWPATAHERNEFWQRLRLMLLGELT

>LIon-TLRα1

-LNKLPSIKLIYLNQNNF----------------SQISLDHSENLLTEILE--RSIKGHV
KVLDLSYSSIADIDNEFLEKLSHLTHLYLNGNKLTKLTEHTLSLQERLTELHLYNNTWDC
SCSAMLMIKLLNRLIARKTLVRPDEIVCVTPERNRGRMVYMV---DDELCE-TKLAFYIE
QHFVLNMTLALLVLKFFKRETIQLLTL------RAIVNDDD-TSMVFDAFVSYCEDDRVW
VEQELIPCLQQEPPYKICQHRLNFVPGFTVQQNEFNAIKHSRRTIIVMSNAYLGREHCQ
YEFKTAYNYWITEKEPRLVVVKYPDVEDRN-QETCHAYFRKFTYLEKDE------NTFDRL
LAFMPRR--

>LIon-TLRβ5

AFVGLEKLEQLELNDTDIGEI-TEDMKSVFHPLKGLKRLDLDKSRFSNIPDGMFMNQVN
LVELHLADNKLRAIDHSVFRTLLRLKQLDLRNNRLGFISGEVMARLPSLQTFFFTGNNF
GCHCDLAEFTRWLKKNDFRENQCELYNEKCVVPPSMKDTSILDYQPTWLG-
CDNLILTVSSAFSFFLILSTSVAIHAYRRRLSIRYWFVVQKMRRAEYEAL-
DNSDIPFDVFVCFEKNDRFWVEKTLLPKLEDDIRFRVCTVDRDLDPGRPEVMNVARGI
RNSRNVIFVVTRELIQTAWCEYEICLAETQSLQEGNCRLIIIFLEKFTWEELPLCMKRLLS
HVNFLRWPETAHEQEDFWRRLRLVILGETT

>Llon-TLRβ2
ALHGIPNLEVLDIHGNKFDDMTQD--
ANFLSMFRNLKRLSMGKMGLFFLEGWIFDNLTKLERLELSFNALGNITARWFKNLKYLK
KLEMRDCRIATVNVKSFAFLNQLNSLDLRDNPFSCDCSIQWFLNWSKHHGNQLYMFN
RKDYTCASPQWLHRMPLRKFTIP---SCYKTLLTGLSV
AGVAIIVCFFVLAFFSRYRWHIKYKLFKLKIWF-QYEEL-DGSKYEFDYHVHYDDQDVSW
VVNTLIPELEDKRGYRLYIKHRDSSLCQYIIENIRYSIEHSYKTVLCLSNQFTQNPGTQF
LLSFIINKLVNEKKNILVCILLEEIQGENLLETLEEVLTEKSYIRLPEDREAMEYFWSRV
DEALH-PRN
>Llon-TLRβ1
VFQNLKRLKILRLTNNDLGPQLKDTKGELFAGLENLEELYIEKNDIQELTGDVFRHLKGA
KMLELGENAISQWGTSTFSQNSTLKHLNLSRNRIATINEPSLADLKLLTTLTLTANPFSC
DCGLVWFRRWINST--NVTFPELELYTCNSPVRMAGIPLLKFDPDSLT-CKDPYILGGSL
GG-VVILILVISLVIYRYRWFIKLRAYRIAKAVAGYEPI-PGDDLRFDAYISNHRDDRRF
VLDELLQNYDNNGGFRLCFNERDFVPGEYDLTNVTENMSQSQRGLIVLSLQYIQDHFQ
DFELHLLLKEANLRA-FGLIVIELEEIPPNRIPNGLRRIFEEGDHLSWSEDPNQQQL
FRERLTNKLQRRPQ
>Llon-TLRβ3
PIAGLNKLYKFGTTGADF----IN--RGLFINKTELQSLHIGSGRITMNHLDILRNVTSL
KSLRLEKMDIREIPH--ILHLKHLKSLTLVSNKIEMIPQHFIPKLDGLKTVDLAFNPFAC
NCSLMPFSNWLRNASRLATVTNLDVTLCFSPASYKNTPLFNFEKD----CRNPIILPSVL
IP-LVLLILVVTAVSVQYRGYIRYACMLVRARWRGYDALNEGRSFKYDTFVSYNREDA
AWVLRVLRPKLEDEVGYQICLHDRDFTVGEDIVDNIIMSIDESRKTILVLSDNFAKSQWC
QLEMSLAQHKLFEDNRDVLILIRLGEVAEENMTRTMRMLMRTKTYITWPQNEEGIDLF
WRNLIFALRRPPG
>Lrub-TLRβ2
FFKGLTNLQNLSLSWTDFGEVDHSQGADIFDQANTLRSLNFLGGFITHISAGLINDLHNL
THLNFQSNSIGYLFSEWFENLTNLKVLNLNDNKITTVVGANGRFFRSLSQLTIGENAFD
CDCQLRFFSEWLRGLDDQIRVSDMDKAVCLTPIAYENKRIKDF---KSAVCS---GIAFTS
AAAALLFIILSVIVGCYCRHDIKYMMAIRRLHHR---TLR-GSKLIYDV----NDTDRQW
INTNIGNITDKD--FNITTNHPDIVPGEARSNPLSKQINQCYYTLLLISNHFKDDLWPEV
HANLTVQEI---HNIRFVIVLIDNLRLRDLPRELKALAKQRPCFKWPTETLRRRLFWRQL
ILALIKRRA
>Lrub-TLRα1
-LNKLPSIKLVYLNGNNF----------------SQINLDHSEKLLTEILD--KSIKGHV
KVLDLSHSNISDIDKQFLEKLPYLSHLYLNDNMLTKLTLNSLRIADRLTEIHLYNNTWHC
SCNMTQMTILLNHLIARKTLVRPDEILCATPERHRGRRVYML---DHELCG-TDSMSFVV
LTSIIAITLATLILKFFKPKTLQVALP------RAIEDDDD-TGMVFDAFVSYCEDDRVW
VEEELIPRLK--PSYKICEHKKHFVPGITVQENEYNAIKHSRRTIIVMSNAYLERGHCRY
EFTTAYTYWIIEMKPRLVVVKYPEVVDQNK-ETCHAYFRTFTYLAKDG------NTFDRL
LDFMPEK--
>Lrub-TLRβ1
ELHGIPNLDVLDIHGNKFDNMVQD--ANFLSMFPNLKTLQMGKMGLFFLKDCIFDNLTKL
EKLELSFNALGNIPARWFKNLKHLKTLEMRDCRIATVNEKSFEFLNQVTHLDLRNNPFS
CDCSMKWFLNWSKYHNNRLVNYNQKDYTCASPPTLHGLPIKNFTIP---
SCYKTLLTGLSAAGVAVLICFFSLALLGRYRWHIKYKLFKLKIWY-QYEEV-

DGSKYEYDYHVHYDDEDVTWVLNTLIPELETKRRYRLYIKHRDSPLCEYIIENIRYSIEH
SYKTVLCLSNKFTRNPGTQFLLSFIINKLVNEKKNILVCILLEEIEGENLLDTLEEVLTERN
YIRLPEDREAMEYFWTLVDQALH-PRN
>Lrub-TLRα3
-LKAIPHLTNFIHKDVQV----------------ESSILILSHNNINSLNP--YEHLKHF
TQIDLSHNNLSFIDPSWFNLFKGVSQLHLNDNNLTQINPKDIETFRNLEELHLYNNPWD
CGCNKIWFKSWLSYLADAGVVMKPHLITCASHHWNKEKIVHNL---YVSFCQ-TFIAMVLI
--VLVTIFFIFILM-----YKLRLYIFSFNVHLR-----EEGENMESDAFISYNGADYDW
VKNKFNIRL---MKYKLC-NLRQAVDGRDFLENIETALMTSKRTIVVLTKHYLEDEECTR
EFKIAREYWVDIKRHRLIVLKH-GVDIDEIDNEIRLFLKRYKLIEMDR------NLWRNL
SYAMPNP--
>Lrub-TLRα4
-LKAIPTLNGLIKPDNKI--------------------LLLTGNNITNLNA--VQYLDSF
TTIDLSHSNISAINEYTL--LGKVSKLFLNDNNLTHLLVQGLKIFTNLEELHLYNNPWNC
SCDQKWMKLWLLNYIDAGIILKPHMVTCGSQNWNEGKVIHTL---PEEFCFPTILYIFLV
---LLVCILVG---VKQFIYNNKYQLFEFNLHPC-----EHGENMNYDVFISYCENDRSK
VLKDLIQSRD--PPYKICDPDDSFPRGRPINESIADAVTKSKRTIIVLTKAYALKSYCCE
EFRTALDHWSMDERHRLIVI--KDIETNTIDDKLRQYLREYTYLELDV------NLWKRL
SYILPQP--
>Lrub-TLRα2

TNWPGHIQSFEDKVTEIHLYNNPWDCSCNKLWMKKWLNKLIV--ILVKPDKIICGTP--
NKGQMFYMV---EDSFC--TDKKIIIIL--VISIVLAVLILRH---VFHKLWPDPLPPNRI---
DDSNTSGMTFDAFVSYCEPDSEWVEQELIPNLQQNPPYKVCHHKQYFQPGMPSEWN
EFMAIIHSRRIIIVMSNSYLEREHCRFEFSTAYSYWIHTKNPRIVIVKYPDLEIVN-
REACSAYFKRFTYLAKDE------T-FKRLFQFMPLE--
>Nge-TLRβ6
VFLGLGHITELELNMTDIGKI-TEDLEFVFRPLKNLERLNLANSALKDFPRDLFLNQVNL
KILDLSYNRITIIGDVILSTLKKLRYLDLRRNEITEISGDALRKLTYLQIALIAKNNYAC
TCALRGFTKWLLEHKVTDDVCADYSAGCRSPPQFVGKRLDKFQPSWID-
CDNAIVTLSTLFTIIFCLFTILSIYGYRKRLSIRYWYVIRKIKRKGYLPL-
PALHDLHDAYVAYADNDHAWVEGTLLPNLEDDVTFKICTNERDFQVSDEIIDIVEKGIKC
SDRIIFLITNAYLQATKSDYVMAQAERLYLETGRPHIILIMKEKINFDRVPLSFKRLISHAA
RLHWPENQGQRNDFWKNLRLLLLGERT
>Nge-TLRβ5
VFLGLGHITELELNMTDIGKI-PDDLEVVFRPLKNLKRLNLANSAINDFPRDLFLNQVNL
EFLDLSYNRITIIGDVVFSTLKKLRYLDLRRNEITEISGDALRKLTHLQIALIGKNNYAC
TCALRGFTKWLLEHQVGGEVCADLSASCRSPPQFVGKRLDRFQPSWID-
CDNTIVTLSTLFIII----VCLSIYGYRKRLSIRYWYVIRKIKRKGYLPL-
PALHDLHDAYVAYADNDHAWVEGTLLPNLEDDVTFKICTNERDFQVSDEIIDIVEKGIKC
SDRIIFLITNAYLQETKSDYVMAQAERLYLETGRPHIILIIKEKINFDRVPLSFKRLISHAAR
LHWPENQGQRNDFWKNLRLLLLGERT
>Nge-TLRα

NSLDYVWVVHTLWNKLEKRPAYRLLLHHRDFIPGGMIMDNIVEGVTKSKRMIMYVTDN

FIKSQWCMVEFRTAHHEALSKNMNYLIAIVDEELDIENVPEDFKVFLKNTTYLKRNE------
HFWDKLYYALPQRGP
>Nge-TLRβ2
VFKNLKHLKKLKLGKNELGGLIDDKKGELFAGLDHLEQLDLRMNSVKELTDGVLKPLKG
MKKLELGRNSISAWGPATFSQNRTLQHLNLSNNNIATISKSSMSTLTSLVTLTLTGNPF
SCDCGLVWFRRWIDHA--NVTFPGLKSYQCNSPPVREGLLLLKFDPNSLT-
CIDPYVLGGSIGG-AVILCLVMTLVMYRYRWFIKLRAYRFGQAMREYEPI-
PGDDLHFDAYISNRDSSREFVLGTLLPVFDNNGAYRLCFDERDFEPGEYVLTNITNNIA
QSQRGLIILTPEYIHDKFYELELHMLLEEANKRP-
FTIIVIELVEIPPNRVPNGLRRIFEARNQLTWSENPDEQALFKDRLTNKLERRPQ
>Nge-TLRβ4
------DCGYLNISYFKFDDQLELANPVPFRLTPNIAEFMVSAARCFVQPQYK-------
---------LVSLLRAILRDEYITWHKKMFLLRVVAVVLPLLADGRRASDVEYVKKGVNV
NCTEKGWKDVPKNLPKKIASIRRAFVGLTKLESLDLTANKIRXIPQ--RCHKRLLAGLCA
FGVLLIVTALGLGLFSRYRWKIKYKIFKLRLWFYQYEEL-
DGSKYEYHFLVHYDDSDFPWVRDMLIPELEHKRGYRLYIKDRDSRLCEYILENIQYSIE
NSYKTVLCISNQFTQNSWCQFLLRLLIQKLVNEKKNILVCILLEEIGGENLLDTLENVLTQ
KNYIRLPEDREAMAYFWTCVVEALH-PRN
>Nge-TLRβ1
VFKNLKHLKKLKVGKNELGGLIEDKKGELFAGLDHLEQLDLRMNNIQELTDGVLRPLKG
MKKLELGRNSISAWGPATFSQNRTLQHLNLSNNNVATISKSSMSTLTSLVTLTLTGNPF
LCDCGLVWFRRWIDHA--NVTFPGLKSYQCNSPPIREGLPLLKFDADSLP-
CIDPYILGGSIGG-AVIICLAMTLVMYRYRWFIKLRAYRFGQAVREYEPI-
PGDDLEFDAYISNHHSSSEFVLGTLLPNFDNNGAFRLYFDERDFEPGTHDLTNMGKKI
SQSQRGLIILTPEYIQDKFHELELHLLLEEAKKRP-
FTIIVIELVEIPPTRVPKGLRRVFEARDQLTWSENADEQVLFKERLTNKLERRPQ
>Nge-TLRβ3
--------------------------------PEFEDFEVVSRKVFEVERWSFDKAVHICGWVLRF--
VYNLRHPNLRHSGPLSHEEMFLLRVVAVVLPLLAHGRRASDVEYVKKGVNV
NCAEKGWKDVPKNLPKKISSIRRAFVGLTKLESLDLTANKI-------------------
---------------SRYRWKIKYKIFKLRLWFYQYEDL-DGSKYEYHFLVHYDDSDFPW
VRDMLIPELENKRGYRLYIKDRDSRLCEYILENIQYSIENSYKTVLCISNQFTQNSWCQF
LLRLLIQKLVNEKKNILVCILLEEIEGENLLDTLENVLTQKNYIRLPEDREAMAYFWTCV
VEALH-PRN
>Pau-TLRγ14
LFRAVGKLEHLFASRNMFGLFKPGDLVTTLSGLPNIKTIDLSINRLSYLPRGIFSECPNL
THLDLQRNGMKSVHL--FSSLPSLKYLDLSHNEIKGFSKEQTEDF----FLKLENNSFEC
SCDNIPFIEWIQSDGSNDTVLNKSKLICEFADG-RMTALVDVDLNKLYDCMKIIIAVATM
----AVVLLSTSLIMWRYQWYIKYWIYVLRLRHR------DDGTEPFASYISYADNDYDL
ANT-VCTKLEE-SNLPVFFRDRDTSLGTSIFDEYFRGIESSRKCILCLTDSHLNCAERYF
ELQMSMVR----GKGFLIPVVVGNLALEKLPKPLRRLLRDDVYFEWPKTDLEEEDFWKSL
IAAVLTRKG
>Pau-TLRγ6

-NCRRSLLGF-------------------------E-EH-QICIKRTWGIAKCHRS---LDAVFSYCLH-----------
------------------HQHLILAAFTGKMVTCLSSEDRHWVHDVLRARLEENSDFGLCIHYRNFL

PGRNIEENVIDAIESSRHSMLVVSRNFLKSEWCIFEMHMARNIFRRQQKDVLLLILLEDI-
VQDAPLTLVNLLRSRTYLKWPADDVGQEAFWERLKETLKREPE
>Pau-TLRγ5
RYKAFPALKKLILAGNKLYIMLRDRKTKHFRYLNNLTHLDLAYNSINELYPETFNDLPAI
KEVLLRGNRLISVTSMNITGMPSLKNISFAKNNVRFVSENVLHLWHG-
KSVDFSQNPFNCSCLFLPFLRWFNNVSSTVTLLNSDHYRCGDEKKT---
YVRKISLDALTKCTSAWMIISIAMSICVALCITLGSVLYRHRWTISFWINFAARKSHSYSP
H-MRQRFQYDAFVIYSSEDRHWVHDVLVTRLEDESDIGLCIHYRNFLPGRDIEENVL
NAIENSRHSMLVVSRHFLKSEWCIFEMHVARNVLRQQRKDVLVLILLEDIPVQDAPLTL
VNMLRTRTYLKWPANDVGQEAFWEMLKETLTQEPE
>Pau-TLRγ2
IFQNLTSLKTLDLSHNLLADKLKDEYGTIFQNMTTVVKIDLSGNGIYKLHINTFHHLKKV
REVILRNNRLASLPAVHIEYLTALKLVDMTSNRVEYLSKASLDSLDK--TVLLADNPFNC
TCEMLVLFRWLHEYLEGLHIKDNHSVYCSHNTSLR---LSNFQFDDLQKCT-LWIWLSLS
TILLLTALIVGLGVAYRRRWTIRFWLIAAR---QGYHRL-PMPEYKYDAFLCHSGENTWW
A-KRIQDHLEDDIGMKLCIYYRDFPVGVPIVECVNDAIVDSRYIILLITKSFIKSQWCIY
EFFMAKSKVFCENRSRLIVLVMEKLTDDVLPRTLQNLMKDSVYLEWTDNALGQEQFW
KRLRERLRTEPP
>Pau-TLRγ13
QFKAFGNLEHLMASRNRFGLFRSEDLVTTFSNLPRIRTIDLALNSLTSLPEGMFSQCPL
LEYLNLERNGIKVVNI--FATLPSLKLLDLSHNEIKGFTKDQTDDL----VLQMNNNSFEC
SCGNIPFIEWIQSDASDGIVLNKNNLTCQYEDG-KITSLRAVQ--GLYQCIKIIITVSSL
----TSVALCTALIVWRYQWHVRYWIYILRLKRR------DEVSKCFDAFISYSDEDHNL
AAS-VFRKLEE-MGLQIFFRERDTVIGACVLDESFRGIESSREIVLCLTESYLNSDQCYF
ELRMSMLR----GKGFVIPVVVGDVALEKLSKPLRHLLREGVYFEWPTLESEE
KDFWKSMKKAVLTAKG
>Pau-TLRα2
-LTETS----ISLAKNNLSNIGHNYIVNTFKDANCLKELRLDNNKLTQLKRYYFESLNNI
VELWLQNNDITSIDKDSFIHLTHLQRLFLHRNHLHTLPESKL---DSIVEVTLAQNNWSC
ECSFASFRHWLLDHID--IMADITNITCTATSTSRGEELVSF---DINFCNQRLISGLIA
SILTLVTITLSLSLLLKYRDTIKVWLYRYGWRPS-----SADVRKKYDIYLSSTNTEA--
-CRELLAELEDLPRYVVFFPQRDLIPGGVTTNDITEAIKESWRTIVVLSPAYLQDSWRMF
EFLRAHYCSVHTKTNRIIVLLSEPMKADDMEKDIQAYLTSKSYIKLWE------RLYDKI
RYRLPDGRK
>Pau-TLRα1
-LSGIPE---VSLANNEITELNNNDIPNTFRELACVKVLRLDHNQIAHLAAYIFTGLRQL
RELDLQSNLISSIDNRTFHQIIHLEKLQLHDNRLVSLPEPNT---TSMKHITLSGNPWTC
GCDFAQFRRWLILHMD--IIPDILEVKCTLKQTSNNRKLIDLSLINIEYCYESIRNALIS
TIIIFICLIITTIVVYTNRMEIKVWIFRYGRRPY-----KDDFSKPYDAYISYSDKQLNF
VIHELLPKLEQSPHYKLHVRARDDLPGGVRANDIISTLENSCRTIAVLSENYVADEWCLF
EFQRAHYNALHSKMHSIIVVLLHDVKA-DVDKEIQLYIKTGSYMRRDD------KLWQKI
RYALPDTRK
>Pau-TLRβ3
YFKGLKKLDEILLGRNDFSEFDRKSPIEIFSDLRSLRKLNLNYVNLKYLHDGFFAALKNL
TKLILDGNGFSGWSPRAFQYLVSLQHLSIINCQVFTINSSMLQPVATLTRFEGYGNPFA
CECKLRWFISWLEMMNSSTHV---KPYKCLTPKKWHGHSIFEYNTTDDD-

CSDWLLITATASGTVVFVTAAISGIAYYHRWSIRYWMFLARSRRKKEISLRRREDFEYD
CFVTYSSLDTDFVVQEMLSHLEGENDLRVCIHERNFQVGGDITDNIVQSIETSRKIVVVL
TENYVKSEWCKLELNMAHAKLLDERRQALIIIMKEKVSVKLMTPILRHLVRNQTYILWNG
SDILQTAFWGKLVQAIMKP--
>Pau-TLRβ2
YFANLTSLEHLSLNFVDLGIDIDSKPECLFEGLVKLKVLDLSSTALNGLPERLFKDLTSL
ERLILRKNQLSGWNGAVLENLKNLRSLDVSMNQIRTINQSSLRPVSTLNHFQGFHNPYI
CDCNLAWYVDWVGQMHATLKISHKISYKCSN---LKNKSLLQYRPTFFE-
CHRLAILCGSGFG-LFTVIFTVIGLLYKHRWYIRYWIFLLRSRRSTHLEETDGLLYTYDCFI
TYSGEDSNLVTQQLLPKLENEFGYKMCVHERDFKLGREISENIAESIEKSRKVLVVLTQ
NFVQSEWCKFEVNLAHANALHNARQSLIIILVEDVSFEHMTPILRFLMRKKTFLEWTND
AQGQTLFWERLKNAIQQYGQ
>Pau-TLRγ10
---------------------GKGLSTIPRNLPNATVLILRRNSITSIPANIF------
--VLLQRNNIAVVD-ATFKKLPFIKLMDLGHNYIKKFSFEQVEDL----TLRLTGNLFEC
SCKTVDFITWIQAPGTSNVIENKKDLKCASPDG-THQRVVEVSLQGLGYCVQLIISSAVV
S----AMGICVAVVIWRHKWTIKYWMYLL----KRRR--GIDRIRPRHAYISATDDDLEK
ANMIFQQ-IEDKLENSVFWKHRDTVPGRSTFDEIFRGVEESRKVILCITQSYSTCTQ-NF
EVEMSF----ARGKGFIIPVLIGDVPLERLPRPLRRLLRDDIYLEWPNNVAEMPNFWVSL
HEAVMTQKG
>Pau-TLRγ7
LFSHFPSIKVIELQDNLLGLEMPDQFADVFENVRTLLEIDLSSNYLNKLAAECLAGNAKL
TRIHLANNKLDKLG-LRLENFPMLEFLNLSKNAILFLSSNETSALDSI-ILDLSGNILLC
SCATLNFLDWLRT---SVIFSGRNTYTCSY-KG-KPRNLEDVDLENFRECMSVTVLTSSL
VA--GTILVMLATVGWYRRGHIRYVIY------KFRQ--HPNDDLRYDAYLAYESRDCDV
AVEMAAI-LEGDHGLDIYIHDRNAPVPGDHYDSIFDGLGRSKKVILLITDHALRSESWSF
ETDLSL---SIKGKGKILCVVKGHLSIGRLNRKLRYLMADDTYLVWPEDNDVEKTFWRHV
AVAITSKNG
>Pau-TLRγ15
FLAYLPSIEVIELQDNLLGLEMPHQFARVFENVTTLTEIDLTRNYLHNLTAECLTGNENL
AKIHLANNKLDKIGL--VEKFPMLEFLNLSKNAILFLSSSETSALDSIAIVDLSGNIMLC
SCATLNFLEWLRTA--SVTFPKKSSYTCTYKGK--TRNLEEFDYEDFRECFSITVLTSSL
VA--GTVLVLSVVIGWYRRWHIRYIIY------KFRQPPEPNDGHRYDAYLAYESRDHDK
ALE-MTAVLERDHGLKIYIHDRNAPLPGDHHDSIFDGVGRSKKVILLITDHALRSQWWSF
ETDLS---LSIKGKSKILCVVKGHLSIGRLNRKLRYLMADDTYLLWPDNENAANTFWRNV
ALAITSKNG
>Pau-TLRα6
-LRSFPKLP-LHLEDNFLEHVE--------------------------------EYLKRV
TKLFASRNNISDVSEKVLKKMEKITVLYLDSNKLTTLPEYIKKMTRRLTHVNIKHNFFEC
DCNTLWIKYWLRENIA--KVIETQNILCSSG--TKGKSIIYVP--DNKVCEL---VAAIV
LAVTLTIFLVAVVSVYKHRQEVKVLLYKLQWHPK--E-LDEDETKIYDAFISYCQKDYRF
VCNDLRSSLEQNPPYKLCIHERDFMAGAPIYENIMNSVKLSKRMIMILSNDFLLSEWCM
LEFRTAHQKVLKEHSRYLIIIALGDIVSRNTDEDLQAYLKTNTYLTVDD------LFLERL
RYALPRPTS
>Pau-TLRα4

-LTSLPKLPRTNFSNNHLTEVT--------------------------------
AYFPNIIDLDISGNNIRNVSDAALIQLRNIKVLNVAKNKLTTMPRRLLESSANSTAISLSGN
SWNCTCSEVWFIKWVLSKSS--VVTDSHGLFCSHP--MRGKRFSDDV--
VTERCDADYTAVAVSVGVSSTVLLIVIVIVTVFSEDIKVILFKWNIDIR--N-
VDNCSDRNFDAFVSYSSLDGDWVRNHLLPLLENDPPFKTCFHERDFIPGLPITENIIQAI
QKSKRTVLVVSKNFIDSEWCQFEFLTAHKTFLETKENKLIVIVVESVNLRSLNPKLRAYF
NTKTFLKVTD------LFKEKLYYAMPRLME
>Pau-TLRα3
-LTSLPAAP-FHLSKNSINKIE--------------------------------DYLTRV
FNMDLSYNNISVIDEDAFQNMKQVQSVDLRGNGLTTLPLLLKQGTRNLQKIFLGENKYN
CSCENAWMKNWLKRNSN--ISAGLEDIVCDSP---KKFRAIKVI--EADNCREKFTQEVVV
SICLCLLVLVLSIIIIIYRDLFRVLMYHFNVRIH-----EEETDATYDAFIAYSSLDGEW
VRNKLMPLLENRKPFKVCIHEREFLPGLSVADNVHRCMDLSRRNVMVVSQNFINSEW
CRFEFQAAHAATMRNKSKRLLLIMLEDIRQDNLGDDIKSYLKTNTYLEAEE------
WFKPKLFYFMPSVKS
>Pau-TLRγ4
RWQATPSLKKLLLTGNRLYVMLRDKKTKHFKYLKNLEHLDLADNNLAEIYPDMFRELPV
IKEIVLRRNRLFSATNLDVSGMPLLKKVNFVNNKIRFVSEGALKLWHG-
KSIDFSGNPFNCSCHFLPFLEWFNNASPTVTLLNSDQYLCRS-----
GAYIKNVDIKRLNQCKKTWRVISITVAIGVALSITFGGVLYRYRWTIKFWIVFAARRSR---
EVDRCRKFRFDAFVIYSSEDRYWVHDVLRTKLEDGNDFGLCIHYRNFLPGPPIEENIIG
AIENSRHCILIVSRNFLQSEWCIFEMHMARNVFRQQQKDVLILILLEDVPVQDAPLTLVN
LLRTRTYLKWPADDVGQEAFWETLKDTLRQEPE
>Pau-TLRα5
-LTNLPKLP-LQLSDNRIEELK--------------------------------DYFHRL
LELDLSNNGLRTMSDIALVKLTNITTLKLNGNRLRTLPRSTETWSQSLRQLALHDNLWE
CTCDTMWFRDWLIQLGS--VVQEPDSIMCFKD--EEWKPIKKA-----ILC--DYIPLAIT
VSSVSAVLMLAAVLMYIYRMEMKLLIFRLNWHPR-----TEILNKKYDAFISYSEEDSMW
VRRLIQLLEVDDPPYITCFHHRDFIPGVSTAANIEMAVHDSQCTIIVLSPAFVQSEWCMF
EFQVAHACLMDNEIGKVIIIIKEDIEVKKLQPDLKSYLRTMTYVKASD------WFSEKL
YYALPQKDK
>Pau-TLRγ1
ILQNMTSLRTLILSDNSLADKLADEYGSLFQNMTSVVEIDLSYNGIYVLHSNTFYHLKNV
RKIILRNNRLASLPSEHTENLTVLSFVDMASNKIQYLSKAFLDSLNR--TVLLSGNPFNC
SCGMLVFLHWLADYDDEDKIQDYRKLHCHRKPRLH---LSDFRFADLEECT-LWIWLSII
TMCTLAVLTVCFGVTYHKRWVIRFWLVATR---KKYNRL-PTTQYTYDAFLCHSSEDVRC
V-ERMRERLEEGSRLKLCLYYRDFPLGVPIIECVNEAIADSRYILLLITKNFIKSQWCIY
EFFMAKTKVFCENLSRLIIVVLEELTNDVLPRTLQSVMRDNVYLEWTDDVQGQEHFWQ
RLEECLGTEPP
>Pau-TLRγ12
LFRAIGKLEHLFASRNRIGLLTSQDLSATFSSLPNIRTIDLSLNQLSSIPESMFSLCPHL
ERLNVQRNGMKSVNL--FANLQSLKFLDFSHNEISGFTKEQTIDL----ALQMNNNSFEC
SCNNVPFIEWIQHESSNDTVLYKENLTCLYKDG-REEAVISVDLGGLYECIKIIIIISTL
----ISVVLCTALVAWRFQWHIRYWVYILRMKRR------DDV----------SKKXYDLALS-
VLHKLEE-MGLLAFFRDRDTDLGVCVLDECFRGIESSRKSIVCLTENYLNSGQRYF
ELRMSMLR---GKGFVIPVVVGDVALEKLSKPLRHLLRDGVYFEWPTLESEEKDFWKSM

LKAVLTPKG
>Pau-TLRγ11
MFRAIGKLEHLFASRNRIGLVTSQDLTATFSSLPNIRTIDLSLNQLSFIPEGMFSLCPHL
ERLNVQRNGMKSVNL--FANLQSLKFLDFSHNEISGFTKEQTIDL----ALQMNNNSFEC
SCNNVPFIEWIQLESSNDTVLNKENITCLYKDG-REEAVISVDLGGLYECIKIIIIVSTL
----ISVVLCSALIAWRFQWHIRYWVYILRMKRR------DDVSKNFDAFISYADVDYDL
ALS-VLHKLEE-MGLLAFFRDRNTDLGACVLDECFRGIESSRKSIVCLTESYLDSDQRYF
ELRMSMLR----GKGFVIPVVVGDVALEKLSKPLRHLLSDGVYFEWPTLESEEKDFWKS
MLKAVLTPKG
>Pau-TLRγ9
FFRDFPSLQTLQLASNRFGLFTESYLEAIFSNLPSIRKIDLADNLLTTVPKAMFSNCTSL
VTLNLRYNPLVTFEF--FSLFPQISYVDVGDCKIQEFKAYQVKFFSSL-KVNVSGLDLDC
NCENKEFYEMIQKNKSLADLEGKQELACTRDGV--RVKLAHLGLSGLESCMYIFVILCVT
---VVSAILISVVIVLYCRWNIKYLVHLTKRKLK-----NQANALYYDVYLSFSEDDRDT
AFQ-LFTGLNN--GLEVFYWPRNSRPGTCQFEEIFEQMGLCKKIVILITASTENSAMQNF
EIRMSLPR----GKGFVIPIVKEDYVIGKLPGPIKNLLRQDLFFLWPEQEKDQEMFYRNV
KRAARSKDG
>Pau-TLRγ8
LFRAVPSLQTLLLAVNRIGLFTETELVAIFSNLQNIKEIDLTDNMLTTIPKVMFSNCTSL
VILNLQKNPLLTFEL--FSLFPGFTFIDLSNCEIQEFKYFQTAFFSSFRVVNVSGLSLSC
NCNNKEFYDMIRNNKSLVDLEGREELTCTHGTE--RIKLVDLDLSSLESCWYTFLIVCVT
---LTSVVIIGVVVVLYCRWNIKYLVYLTKNKLR-----NNANIFEYDLYISFSEDDRDI
AFQ-LFTGLHNK-GLDVFFWPRNSRPGSCVFDEIFEQLDGSKKVLVLVTSSTEN
SVTQNFEIRMSMAR----GKGFIIPVVTEDFVVCNLPQGIKHLLRHDLYFLWPEEDEEKE
EFWKNLERAITTKRG
>Pau-TLRγ3
SYLPCPNLRKLILAGNRLYVMLRDKGTKHFANLPNLEYLDLANNNLTELYPEMFSELPAI
QTIVLRGNRLFAVANMDISDMPSLKKIIFARNRIQFVSEGALQLWHG-KKLDLSQNPFNC
SCQSLSFLRWFKNVSSTVTFLSPHGYLCRDKFHQKSEYVGEVDLKRLEECRKDWIVISI
TVSTGVALLITLIGMIYRHRWTIKFWIVFAARRG-PINSLDRQRRFHYDAFLIYSSEDRHW
VHDVLREKLEEDNEFGLCIHYRNFLPGQPIEENIMYAIENSRHSILVLSRNFLKSEWCIF
EMHMARNIFRQQRRDILILILLEDIPVQDSPLTLINMLRTRTYLKWPADDVGQEAFWEML
KQTLKKEPG
>Pau-TLRβ1
----------------------------MFEGLVKLKVLDLSYTNLKGLPERLFKDLTGL
ERLILRQNQLSGWNDVVLRNLKNLRSLDVSMNQIRTINQSSLRPVSTLNHFQAFSNPYI
CDCNLAWYVDWVRQMHGTLKISYKIPYNCSN---LKHKSLLQYRPTFFE-CHRLVILCG
SGFG-LFVVILAVIGLLYKHRWYIRYWIFLLRSRRSNHLEETDRLLYTYDCFITYSGD
DSDLVTQQLLPKLENEFGYKMCIHERDFKLGREISENIAESI-------VVLTQNFVKSE
WCKFEVNLAHANTLHNARQSLIIILVEDVSFEHMTPILRFLMRKKTFLEWSNDTQGQRV
FWERLKDAIQQRGQ
>Phe-TLRα
-LNGIPE---VSMANNNLAVLNNNTIPDAFMNVDCILVLNLSQNKLTYLDASMFNGLKDL
RELHLQENNISTIMKDTFQRLQKLEILHLHKNSLTVLHEPSI---GSLKKLTLANNEWVC
DCEFAPFRQWLIEHMN--IIQDLINITCVIKEKSKNRKLIDLSLTNIEFCYESLRNALIS
VIIIFISIGVTSTLVYKYRNSIKVWLFRYGWRPY-----QDDFSKTYDAYVSYSDKQLNF

VLHELLPKLERAPQYKLYLRDRDLIPGGVQANDIIEAIEDSCRTLVVLSENYTTDEWCLF
EFQRAHYNALHNKNHNVVVIKLHDIQTEDIDKEIQLYIKTGSFFKRED------KLWEKV
RYALPDMRE
>Phe-TLRβ
---RFFKDLSQL
LTLELGQNKLQGWDPEIFKNTTKLQYFSVYSNNIGTLNKSSMHLLPSLKRFDAYSNPYV
CNCDLIWYCDWLRNMQRKVTIRSDRPYNCSN---LKKRTLLSYNPTFFD-CHQLKIILPSG
FG—FVFVVFIAGLAYRYRWYMRYWLFLLRSRRNKHLEEHERLCYEYDCFVTYSG
EDSEWVIQEMLPKLEQEFRLRACIHERDFELGHDIYENIAESIENSRKVIVILTKNFVKSE
WCKFELNLAHANTLHNACQKLIIVMKECVPMNIMTPLLRYLVRKRTFLEWSNDEQGRT
LFWNRLNIALTTAAG
>Ese-TLRα
----L--LNNLDLSSNRISYIP----PRLFYKLEYLATLNLRSNQLTELDI--YFYLPRI
QTIDLTFNRISRFTNEVINNLPSLKQADLRNNLITSFDDYVLRLYRSL-TMRLDNNPLNC
DCAKI-FTQLLRNSVDTTNI---FRALCQT---FNGKSIFNFSLN---ACSSLFQIAGYV
IGLLLLLLMILYCLILAICFNCIPFFYVCPCKS-GVK-----RDKEYDLFISYNRANEKW
VKEQLVPFIKENENYILHYNNEN-KLDEVFGPYVKDIMSKSSCILFILSDAFLLKEWNNK
DLRQHLRYLITKEKTRFICVQMHDICDEEVEEYFTDKLQIPRFVSLENDE----LFWKKL
AYYLPKPKS
>Pcau-TLRα2
-QSEIPNVS-LRLDGNNVTTIHNSVIPGSFRDLNNLIALYLDGNEIEEIGDDQFNGLADI
KELHLENNMLVNISTTWIDVTPMFSMLALHGNAFSKAPEAIY---IRSSEYTLRQNPWIC
DCTDEFFLDWLRSNVD--NISDIGEMMCTIPRAITVIEILDF---EMVYCTAGFIAGFVM
LGVLFLTTVLCIALTHHYQHEIKLWLFKYGVRVR---DPESDKAKKYDAFISYHNSDEDI
ILREFVPQLEHETPYKLCVHNRDFLAGEFIAENIVYAVENSRRTIVLLTASFIDSEWCRY
EFQAAHNQAISEKVNRIILVVFEDIPKGKLDKNLEAYIKTNTYLRYDD------MFWSKL
RYALPAVRA
>Pcau-TLRα1
CLEKIPGLTKLSIAHTTIRSVV--YMRDFFKYHPNLTYLDMTGTRFSSLTTEAISSLDHL
RVLRLRDTGISSIPD--FARL-QLKELDLSYNNLMRIPVALL---DSLKHLDLRENPLIC
ECSTIDFMHAAQRFGLVGYLDDPDALSCTFSDQ--SIALRKVHIQ---DCG-VINIFAIV
MASLIL--LAIVVVTYRRRRYIAYYFHVTAVRLKRYEPA---GEYEYDAFVGYST-ELNW
IINFLLPKMENENPYRLFLEERDMPAYGMQVSNIVAFMDKSHTVILVITQTFLTDVYCNF
MLKTAAM------RNNVIIIFLETIATEEFPAELRVLQLHSTCLHWSENRNSQERFWKAI
EYAMPQDPS
>Pcau-TLRα3
-ITEFADWAEIFLDGNFLDDFN-----------------------------GLNLEKPL
TILSLSQNNIDEGGLSLKEILTHVTYLGLTENNLTQLPDKDFMLAASVQVISLNGNPFRC
DCETSYLKRWFSNNAQ--RINKPNETFCTSGPLYRRTAIADLP--DDVTCDSTFPYVYLS
LLVVALLAGVA---------GVGVYVC------RGDDGDESTG-KEYDAFIAFSSQDFEF
VARTLVPGLEGRPPYRLCVHDRDFHAGKLIMDSIIQAIEVSRSTVLLLSNHFIQSNWCKL
EFQASFIEVLANPRYKLIVIVCEPIEMDSLEPDLRFYIKTHTYLEIKD------KFWEKL
CAALPRPLA
>Hsp-TLRα3
-LTKVPYVP-LFMDGNDVNRLPNSVINGSFVGVSNLRILRLDGNLLQNLNGFEFLPLGNL
HELYLHDNLLEFVAKATFAALGHLKVLTLHNNRLHRIPSDLF---QHLTELTLSVNSWIC

DCENSTVQVWAESISD--IISDINLTYCLLGIT--GENLSEFNDS---LCMENFLYLILI
LVLIFIICVILAFLLYRFCYEFKVCLYRYGWRIN-----MEDYHKKYDAFVSYSTRDELF
VLEEFVHRLE--PQFKVALQYREF-PSSSVADGIMDGAHKSRRFVIFITENFLHYVWKEP
ESKSAHQQVLWDTRNQVIIVMLTERPDDKFEPDLRLYMKSKTCLRWND------MFWDKL
YYTMPDIKR

>Hsp-TLRα4

-VSDLKEFEFMFFDGNFLEAFF------------------------------NLNLTKPL
LVLSLMDNNLRADSLSLKDILLHVTHLSLSGNNMTELPDKKFMQKRDVENFIITGNPFR
CDCHTLYLKNWFQQNSD--VIVHANATFCKFGPYYQQTPIMDLP—DEVTCGATLPDV
QVNLATLLFAAPVT---------GVVVFLYNYRR—QSVKENE GVGIKEYDAFIAFNSNDFDL
VAYTLVPVLESNPPYRLCVHDRDFPAGKLIMDSIINAVEQSRATILVLSNNFIKSHWCKL
EFQASFIEVLGNPKYKLVVILCEDIPIDSLDADLRYYLKTHTYLELND------DFWPKL
IAALPPPMG

>Hsp-TLRα1

GLFNIPNLKVVDLCSNNLTRVP----QKLFHDVPTLESILLVNNSISFIHREDFKNLPAL
QLVNMSLNVIEGISPDGFIGVDNLNTLDFEANSLVSFAANNIGFLSKLREVDLQGNVMK
CGCFEISFSELLSSQ--RLTF---TDIHCVTPENLE--QV--F------HCPKSWLLYVCI
VG--VLFLFVILIIIVRFCSRVKIACHRYGIRIR-----QQPKGKTYDAYICYSRSNEHW
VSSTLVPVLESRPPYKLCVHNRDRPAGDSSSNSMVNAIKQSKVTVLVLSDDFMTSDW
CMVEFSPLHQSMSSY-TNNIIPIVLENIESRNVNTEMKRILRNKQALHVDD------YFWDKL
YYMLPDAEA

>Hsp-TLRα2

--TAVPEIS-LHLDSNDLSTIGNSEIAGSFRDLSKLTLLDLEGNSLTDVGSGVFTGLQSL
QRLHLSRNDISHVDERAFEGLSRLSALFLDGNALALPAAALY---ANASQFTLSGNPWIC
DCSMEFFFSWLKVNVD--RISDIGSTLCTV-----EMPIMDF---ETAYCSSAFIAGLAV
LAILFLLTVVGMAVVYRYQYEIRVWVFRYGIRRR---YPESDKNKLYDAFLSYHNGDEEM
ILKEFIPRLEYERKFLLCIHARDFVPGEFIAENITQAAENSRRTIVLLTKRYLESEWCRY
EFQAGHNQAICDQVNRIILVVFGDIPKDKLDSNLQAYINTNTYIRYDD------RFWDKL
LYAMPDPPI

>Rva-TLRα

-LTTIPMLP-VYLDGNRLPSLPNSRINHTFNGLSQLRNLHLHHNQITILRGGEFSQLVSL
EVLDLSWNDIHSIHEHTFLTLTKLRVLNLAGNQLDSLITLPL---PTVSQLFLANNVWEC
WCNEERLTEWLVRFTA--RIQDIHHMHCYDRSQ--AL-LRDMPRE---RCSASFVVVGIV
LGCVCFLVIVLVAFVLRYRYEIQVRLYRFRLRLS-----EEDYEKICDAFISYSDLDEHL
VLGELAPRLEFSPKYKLFLHYRDHPLGMRTPESIIQGVQLSKRTILVLSENYLKREWAKL
DFKTAHQQVFKDKKNKIIIVLLGDIQMKDLDVDLRIYLKQNPCLQWGE------LFWKKL
YYALPDPEP

>Hex-TLRα

-LTSIPMLP-VYLDGNVLPSMPNSRINHTFNGLSHLKVLHLHHNQITVLRGHEFDQLVNL
EVLDLSWNDIHSIHPATFSQLTKLRVLNIAGNQLDSLVALPL---PSLTQLFLANNLWEC
WCNDDRLTDWLVRHSG--KILDIHHLHCYDRSQ--AL-LRDMPRE---RCSASFIMVGVI
LGCVCFVVILAIALILRYRYEIQVRFYRFRMRLS---SQDEDYEKMFDAFISYSDQDEHL
VLGELAPRLEYVPKYKLFLHSRDYPLGTRTPDSVIQGVQMSKRTILFLSENYLKREWSK
LDFKTAHQQVFKDKRNKIIIVLLGDIQMKDLDVDLRIYLKQNPCLHWGE------LFWKKL
YYALPDPEP

>Pcap-TLRβ

MFSNF-SLKELYLGDNKLGPAFEGNLGHLFDNLTVLSLLDLSFNDIDIFSIDQFSSLSAL
KVLNLNHNKVSIFPPDVFDGLKSLERLNIKANKITVLEAGSFQLMKKLKEIDFSENPLQC
QCDVMDFFHWINFT--NLTITHWNDYFC--PQR--NTSLKEFLIMEAEECLHNIVIICAI
TISSLVIFVLLCVLAFRL-YRFIYVRASVEVNTQKSTTIKKNKVKCYDAFICYTSKDADW
IPALFKEHLGEARKLRLYFHDNHKHIERTTSWDVMNKVDSSYKVVFISTKNFVQTDWF
QWESMMLMF-----QDCAIIVGLEDIPTMNMSYTLQWLVRTKPFITWPVLDTDIGLFWDDL
AIYIKER--
>Lloa-TLRα
-LEPSPDIP-IYLEHMEIPVVRHSEIPLAFNTLPSLQLLDLSGNYLMRLTGDELYRTNKI
TTLLLHNNHLMSLGDRLNEVMPQLKTITLHNNKLQDLPLSIEQ--KQITDITLGSNLFRC
DCSPRFIQYWFSSNLD--MIHDVSDIFCVENISNFGDDIFKIP--MTQIATASFLIITAL
LAVALITIGLICLAVLFLRKTKSVIVQRYKVPPF--GTHTTGSSPLFDAFISYSKKDEKL
IIDTLYRQLES-EEYILCLLHRDSPNYSTISDELINQMECAQSLILVLTQHFLNNEWKTL
QIKTSHQIFAKNRHKKLIALLGDGIEPNQLDAELGQILRKNTCIRMND------LFWNLL
HSALPVRIA
>Ovo-TLRα
-LKPSPDIP-IYLENMEIPIVRYSEVPLAFNTLPSLQLLDLSGNYLMRLTGDELYRTNKI
TTLMLHNNHLMSLGDRLNEVMPQLKTITLHNNKLQDLPLSIEQ--KQITNITLGSNLFRC
DCSPRFIQFWFSRNLD--VIHDMSDIFCVENISNFGEDIFKIP--MAQIATASFLIITVL
FAVALITTGLILLAMLFLRKTKSVIVQRYKVPPF--GTHTTGSSPLFDAFISYSKKDEKL
IIDTLYRQLES-EEYILCLLHRDGPNYNTISDELINQMECAQSLILVLTQHFLDNEWKTL
QIKTSHQIFAKNRHKKLIALLGDGIEPNQLDAELGQILRKNTCIRMND------LFWNLL
HSALPVRIA
>Ael-TLRα1
-LEH------LLLTNTSIDRVP------------------------------------LAQLSLGRNAITEIGVETFAN
ASRLSFINLSHNGARSFRKNVTDSLESSTEIK-ADSSLECECLG--EREWLGSSAE----
TSVTNLHCLAPQINGGEKRHRLQV-NQEIADAWTIVLIAIFSILLVFIIVAIIVILRFKVE
IQAFVFNFGVRIK---LPNDVGDKIYDAFLIFSADDEDWVVNTLLQKLETAPPYR
ICIHYRDFVPGNPIIQNVMDSVANSKSTLAVISDGFINSQWCKYEFVTAFQQTLKNAAHK
LCAILTQKIEPQLLKSNLQFYLKTNTYLEKSD------MFWEKLFFSLPDP--
>Ael-TLRα2
-LEH------LLLTNTSIDRVP------------------------------------LAQLSLGRNAITEIGVETF
ANASRLSFINLSHNGARSFRKNVTDSLESSTEIK-ADSSLECECLG--ERE WLGSSAE----
TSVTNLHCLAPQINGGEKRHRLQV-NQEIADAWTIVLIAIFSILLVFIIVAIIVILRFKVEIQ
AFVFNFGVRIK---LPNDVGDKIYDAFLIFSADDEDWVVNTLLQKLETAPPYRICIH
YRDFVPGNPIIQNVMDSVANSKSTLAVISDGFINSQWCKYEFVTAFQQTLKNAAHKLCA
ILTQKIEPQLLKSNLQF-------------------------------
>Isc-TLR3
-LTQLPTLP-LDLSGNKLESLDSN----------------------TA---GLAKKAPFL
RLLNLSDNLLSSIDPSEIPQ--GTDELFLRGNRLSRFPIDLVSKF-NMSILELAGNPWSC
DCEDYAFRQWAEAYTD--VVEDAEEITCAKGPN--LKRFMDL---GQKLCPS--LSYGLP
LLVLLIISLAASTAYLRHKRAIKVWLYRGVCSS-CIKEDDLDEDKIFDVFLSFSSKDSMW
AYEQLIPGVEA-
HGFSVCTYDRNFKGGFLLQDIIHEAVSCSRRTLLLLTKNFVESEWCRW
EFRVAHHQALEDKINRLILVLVDELAPGLVDEELQLYMQATNYLRWGE------HFWDKL
IYSLPKKDA

>Isc-TLR2
-FTQLV-LRKLMLRNNRI------YIDGTFRNNGNLKYLDLAENRIEWLGKRAFSGLVNL
DLLSVSDNFLLHLNGSV-SHMPQLRILNFSHNA--------------IQ--TLYGNDFYN
DPELT-FYAYG-NNLS--TI---GAFQ-TSPKL----RM--F------ACA---------
--------LTASTAYLKYKREIKVWLYRGLCSR-CIKEDDLDDDKLFDVFLSFSSKDSNW
AYNELIPKIES-HGFSICTYDRNFKGGYLVQDIIHEAVACSRRILLLLTENFVESEWCRW
EFRVAHHRALEDNTNRLIVVLVDEVTSDAVDEELRRYMQVTNFLRWGE------HFWDKL
LYSLPKKDS
>Isc-TLR1
-LTDLPTSP-LYLQSNSISSLV-------------------------------APRWENL
TEVYLDENLLSNLDLTT---MRRLQILSLTNNRLRSLTPQLMGMLSSL-SLSLSGNPWIC
DCSTFSFKTWLRGHVY--MVKDYPDIACGD-----GVRINEI---PDSYCPVKQLAAVTA
ICVLAVLLVVVSVLYYRNRQTIIAYVYHFHNVFE-----DLDEDKTYDAFVSYSSADRDI
AMG-LLNSLESEEMFKLCIHERDWLPGYNISWNIVNSVQNSRRTILVVSKDFLE
SVWFQVEFHTAYYQMLEDRVDRLIVIVRGELPAETLDKELKFLLTTKTYLVWGE------
WFWEKLKYAMPHRRQ
>Isc-TLR5
-YGAIPRVP-LYLDGNDMSHLSNSTINRTFNGLVGLQVLHLDHNKVTALHGFEFENLTNL
RELHLSHNRLATVSNRTFVSLKSLTILYLDNNYIVEFQVWNF---PSLSDLRLGHNPWSC
GCRFMEFQDWVHMFGA--PLKDSVAIRCRQNQT--GP-LLEF---NATACT-NYMPLLIV
LPSVVVLLLFVLVLVLVYRKQMKVWVHKYGVRLR---QYAPEVDRLFDAFVSYCK
KDEAFVAQILAPELECHPPFRLCLRYRDLPMSGYVAEAITEAVECSHRTLVVLSEQFLK
SEWCRFELKTAHHELRCNSRHRLVVVLLDDVAVKEMDADARQCLRSAVLLRWGD-----
-RFWEKLRYALPDAAR
>Isc-TLR4
-HIAVPQLP-LYLDGNDIPALSSSTVNRTFSGLRTLRVLRLERNRLATLHGYEFDGLGEL
KELYLSYNHLTHVNNATFVPLKSLEVLHLDHNYILEMAIWNL---PRLNDVRLADNPWSC
DCHFAQFTDFLQNKGA-ELVRDLFSIQCVHNET--ALPLWEL---NTTSCT-DLVPLLVV
LAALFLLLVCIVVLAFVYRRHLSVWFYKYGVRMR-----PAEEEKLFDAFVSYSKKDEAF
VAQILAPELECQPPYRLCLHYRDLMAGGYLTDAITEAVESSRRTIVILSEHFLKSEWCRY
EFKSAHHEVLHSCTHRLVVIFLGRVSYKELDPDIRLWLKSSTFLRWGE------RFWDKL
RYAMPDTRH
>Dpu-TLRβ
TFYGLNSLEYLNMDRCKL----TD--EAIFAGAPRLRHLSMRDNQIVSFGSNPFADATSL
VSVDLFKNRIRGWDTQLFAGSPDLDVLNLAENQISTVSKAMMADIANLSEVDLLGNPID
CDCNLEPLRRYLEDTEDNLLI---KADHCSSPDKWRFQPITSFDPD---HCYYSFVIALYI
LIPTVCLSMLVGYAIYRSRWVIRYYMFRKRLSQ-SSSSMAEEGNFKYDAFVSYS
NVDHAFVAR-MVGMLENPPHYKLCVYERDFTAGNVLNDCIMQSIATSRKVVLVISE
NFIQSHWCLWELHLAQHSLLEDKRNGLVLVVVGKLKLNQCPPTLRFLMKTRIYLEWDL
DPSKQRVFWERLRDALAQKPD
>Dpu-TLRα3
-LNEIPRLPRMNLSSNSI------QIPNSS-----------------------DCYPDVTWLDLSHNGMDESS
MSDWQNLPKLNRLDLTHNNFNSIPNGVVDSWHNL-TYNLNGNPWKCDCTNLALL
NFIYGSWK--RLEDFNQMKCDN-----GQKISEL---SVELCP-SVKYYTIPLPILALLIVCVGIIV
YRNRRVIRAWLYRQLCLWK---EEEENDERIYDAFISFSHHDEIFVNEVLVPQLERPPHY

QLCIHYRDWLAGEWIADQIVRSVATSKRTIVVLTENFLDSLWGKLEFRTAYKQVLTDKR
MRLIIIVKGELPPDKMDQELQTYLSLNTYLKYDD------FFMDRLRYALPHNTS
>Dpu-TLRα2
PLNDLPGIP-LYLDGNNLTELSGSTLNRTFHGLGALQVLQLADNELEELRGSEFEPLDHL
RELYLQNNKLRFISDTAFVHLRSLQVLRLDGNRLLTFPLWRL---PHLNQLSLGLNPWSC
ECRFLAFQQWIAAHPQ--QLVDSDSLHCLMGDQ----QLIGF---EFNSCSADYLPVMAA
GICLFLLGLIAVVLVFVYRQTVRIWIFRYRIRLS-----EEDKDAMFDAFVSYSLKDEQF
VSQVLAAELEHESSFRLCLQHRDFPTSHPGGDPLTLGLAASRRIVLVISQSFIESEWTR
PEVRTALTGFLRLPRSRLVAVLLTPWTDDQSDPELSLLLRSSIIIRWGE------NFWSKI
RYYLPDPTP
>Dpu-TLRα1
-FDDLN-LKTLWLDSNKL------KIGKIFKNIPQLISLQLGSNVIKQLEIGAFSNLPNL
FQLNLQNNQLDILPSDVLQSLANLKYLDLSNNKLTIID-------RNL-TYSLSGNPWRC
DCSNLALLKFIYGSWK--RVEDFNQMRCDN-----GLFFFEL---SVELCP-SLKYLTIA
MPVLALLVFCICTIFYRSRRVIRAWLYHQFCLWQ---EEEENDDRIYDAFISFSHNDEKF
V-DELVAQLERPPNYQLCLHHRDWLAGEWIPDQIVRSVASSKRTVVILTENFLDSF
WGKLEFRTAYQQVLKDKRMRLIVIVKGELPPDKMDTELQTYLSLNTYLKYDD------
FFMERLRYALPHKKN
>Dpu-TLRα4
-HPNLPRIP-LYLDGAQLRALSSSIINRTFNGLRGLYVLHLEDNRIRTLEGFEFSDLESL
RELYLHNNAITSIQNRTFSALKHLQVLRLDGNRLVDFPVWNL---PELNALTLNDNPWSC
DCLFLALRTALHTAGP--KVSDASQLICGGSNR--NRSL---------LCV-DYLPLLVT
TLVAFIAVTLIILFVFIYRQPVRVWCHRYGLRLS---SAATPDSKLFDAFLSYSAKDDAF
VQQMLATNLEYSPTYKLCLQHRDCPSGGGLSETISQAVDSSRRTVMIISPNFIKAEWC
RFEYKSALHQLFGTSRKRLIVILIGDVTHKDLDADLKLYLKTNTYLQWGE------GFWDKL
RFALPDPVQ
>Ppr-TLR1
---KSHIDSL
SSLEYLDVRDNEFACSCDLRWFQTWMTQVP-KMIIPNKNSLHCRSPTDMTQDSVA
NYTTPWIK-CDNFIVVGGVSV--FVVLAIVITLLVGLKRWEIKYWWVFKKARM-GWRK
LRDS---EFDAYVCYHSEDEEWVTQTLQENIEGNVNFKLCIEERDFILGRQHLENF
TDLLNKSHKVLIVVSQNYLKSLWCRFEVGMAQLKLYEDNRDLLIFILLDNLKRKDMPRA
LKCLMLNSRVLPWPKSSQMKSVFWMKLKLALQE---
>Ppr-TLR2
ILTKLPNLSKLDLSSNELGS--SSILPYLFKNLSTLRELDISDNILRTMHEDMFNSLLHL
EKLVIRHNYFKTLPTNIFKRLVNLRSLVMSTNNLCGLYKDQISPLISL-RLDVRRNSFYC
SCSLRWFQNWLQTT--RVWVDDKYKMRCNSPSDQWSNTLINFTIPWYR-
CDNTIMASTSV--SVLFLIISIVILLIFFRWDIKYWWVFSKVSIRGWHHF--
SEEKEYDAFVCYEHSDEDWVMKELLENVEKNNTFKLCIHERDFIPGKRIVDNIERGINL
SHKVIIIVSSAYLSSQWCEFELDMAHIKLTKKKK----------V---------------------------------
----
>Ppr-TLR3
----------LLLGMNNLTGFINSETPPLFDKNSYLKSLDLATNGLHVVHEKTMKSLPNL
QYLNLSDNFLSDIS---ISGMLQLIVLNISKNNWKDTPTELIQSLQKLNCLDISSNPLTT
ECEIQEFIQWSLYT--NVTLTKYNKFECILKNN----HV--FSETVIKTCNSGMFLAIGL
CTSFFILSVIVLTITYRNRWTIKWWLFLARKYLRLREELAEQRNYQYDAFVAFSADDITW

LKSDLIPELEMDRGLKLCIHHRDFQLGVPIEENIVNAIANSRKTILLITNKFVHSNWCMF
EVHMARQRLFDEGKNVIIAILLEEVNIGKLNRTLRNILTSNTYLEYPKNEDGQQLFWIKL
VDALRSNKD
>Ppr-TLR4
LFSCLKNLATLLLQGNNLGGVITDLHGDLFSSKSNLVDLHLDDNNVETLPVNLFKNATS
LKRLSLSKNTIRHWHERLFRKTTSLEYLNLAHNQISLMNKTSLPNLNILKTLNLTANPFA
CTCDLIWFHDWVQNT--KVNLPGVEDYTCDSPQIFQGVPLKEFDPNKLV-CWEKLIMY
ISFPS-LIAVILVSFLIVYKKRWSLRRYWFIMKMRARRKRLMEE—GLEFDAFISYCTA
DKDWVEQTILSKLDKKA-FKFYYDARDDIPGKGIYDNLQYGFEHSRKILIILSTEYFEDK
HADLELQLIPEIEVDAREDKVIFVFKEDVYVNKIPRSIRRKVDNDDFLTWTDDQAVQDLF
WGRLHEELSK-PQ
>Cs_Toll4
SLIDMDNLTTLLLQNNHLGDSLRDVIGQTFSSQKKLKVLNLSKNSIKALPYLIFKNQVSL
KNLSLARNAMTDVS-FSLKTMKMLQFLDLSDNQIEYVTSENMGYLDQIAH
LNLSGNILACMCDNQQFLSWIATT--KVHIIDRGQLKCLYRNKT-TLSLSRIIRSQLKDC
SLWIVMTSCVTGFGLLLILSLITLLYHRRWQLRYLWYIGRKKIDPFH--HDSRLPQIDVY
ISYEQHDVQTVTDYVYPFFERRG-YIVKIRE-EFEATDKLYRVIPDTVNKTRKVV
VFLTPSYCKDYWNTFEFNIAAYEGIYTKRNIIIPVLIGDFSDQNFTPEIRSFVNSKIVLRFP
SQAHRINTFNEQLEHWLQ----
>Cs_Toll13
TFKYLPKLIALEVSYFNMFDMPAQSINTLFSPLSNLKYLMCYNCQIRDDPKLFLSNKSQL
NRIKLDRNYIENISNDTFKSNPLLKTLSINMNKIGHLKASEIDFLNSLDSLDLSHNPFIC
DCDLEWFITWLKST--KTAKVEYQNYVCAYPANMAGTKLTDVHYTYRE-
CHPVWEWVGIVGGPIAVVLAIVSFVLYRKRWSIKHYIYLMRKRR-NYILV-
DGENFLYDAFVAYNQEDSDWVREHLLPVLEDEHQLKLCIHERDFRAGILINDNIVTCIEQ
SKKIIIILSNEFAKSGWCMFELRVAHSKHIEDE-MELVVILLERINGRNMNN
SMKTLLETTTYIEWTEDQHGQLLFWNQLKASMNK---
>Lrug-TLR2
-FLRIPELQK---WRYDIPKLIYLDLSYNDIDRIDINGFPDDG-
LGRINLQFNNITTIRSEDIRA-LEA-IVDISNNPINCGCG-IKLYEF------LGNYEYIR
DLVCHSP--LKGRKIRNLT--QKEICPTH-MVLIVSLCAVVVILGIIIIILL
RYRREVVILVYRLHI---PCQPVDTYDSKNYDAFISYSSKDDDWVLRTLVQRLENEE
KFKLCVHHRDFEIGAAIADNVVQSVEDSRHTVMVLSRNYVDSEWCIY
EFRTALHQSLIERQKHLIVVILEDVPKSELDPDLRKCLETFTYIQVGD------LFWDKI
RYSLG--HR
>Ppe-TLR1
VFKNLSSLKTLNLSKNRLKYMVEDKYGGMFEGLDNLVTLNLENNDIEGLSPVIFLHLTN
VQNLLMSGNRMAHWDKELFTNTQHIKNLDLSRNKISVIDEHALNNYSNFKTLNLEDNPF
ACNCELVWFCKWANRT--KVTLVKFNNYTCSSPKSRQGVLLINFDWRSLV-CFNPYI
IAGSVVG-GVFLMTLIVVMLYRCRWRISLCCYRCGQRC-DYHYL-E-
EDKRFDAYISFDKKDNSFVQEVIMNQFDRNGKYQLCFEPRDFRLGSSIVGSMCVAVEN
SHRAIIVFTNAYLASGRFQMELDLLHNEHLDRSFGMIFVTTGPQLDFRLLPKWLHKSYE
DGKFLVWDENSSAQEEFRQRLDRKLRTPPP
>Ppe-TLR3
VFQRLNSLKHLDLSLNQLGRLTQLQFVRLFQPIRTLEYLHLGHNDLTFLNAPIFEDMLAL
KTINLVENSLKVVQSNLFESSPSLERVLLSDNQISFLDAGMFRRMTNLSFLSIEENEFEC

DCALRQFRDWGHGD--GVMILGLHGERCFASDKRLDSRVTEYETEWIE-
CDHEYIIAGALGL-FFAFATLLAGLVYRYRYDLLWWLLKRRRRR----PT--
TAGERYHAFVSYNSRDSRFTLS-MIRYLEDDIRFKISFDG--FDPASFISDCI
VQCIERSEKIIFVVSRTFLQSEWCSYELRMGELKCFEERRNIMILIFLEKIPVKELPRSLR
TLVRQINYLQWPVDERARDVFWKRLKIALSKDAK

>Pps-TLR3

YFANLTWLEHLNLEGVNLRDVNVDTPDCLFEGLDNLKVLDLTNTNLKGIPTRFFKDLRS
LQELILRQNRLSGWNDVVFDNLTALQFLDVAVNQIRVVNMSSLQSVKSLNRFQAFSNP
YICDCNLAWYADWLRRMHATLKVTYQLPYNCSN---LKRVSVLSYRPTFFE-CHRLIIL
SASGGG-LFILVMLTVTVLYQYRWYIRYWMFLLRSRRAKHVEEADRLIYKYDGF
VTYSGEDSEWVIRTLLPKLEKEYGFSMCIHERDFTLGRDISENIAESIEQSRKVLVVLTN
NFVRSYWCKFEVNLAHANTLHNSRQSLIIILAEDVDMDLMTPILRYLIRRKTYIEWTMND
QGQILFWKRLKEAMQKRGN

> Pps-TLR2

ILKNMTRLKTLILSDNMLSDSLVDESGRMFKDMNSVETLDLSGNRIFILHVNTFKHLINV
KTILLKDNRLASTPAVNVESLESLEQVDLSSNRIQYLSNEFLQSVTK--SVRLTGNPFNC
TCEILVLLQWLAGSEDAMKLVDNTTLACSGDNIIRGAKLVDFHYKSLQRCI-LWIWLSVS
TILTISILTFCIGLAYRKRWSLRFWIIAAR---RKYERL-PSTNYTYDAFVCHSSFDAKW
M-NTLQKELEQEPNFKLCLHYRDFPLGLPIVECINDAIVNSKYIILLITRNFIESQWCIY
EFYMAKTRVFCENRSRLIIVILEHLPEAVLPQTLQNVMKDHVYLEWTDDPVGQQAFWE
RLRENLAAEPP

>Pps-TLR4

-LTDFPTLPDLNVSNNDLTQLP--------------------------------IYLTQLVELDATGNNI
GDISGTALLQMSNIKALYIRNNNLRKLPKALLDSHGNASVLTLGENPWDCSCPNEW
FLKWMTSRGS--VVTDVGDVTCDIP--VRGQRFSDDV--IKQNCETDYMVMAST
VGSITALLIIALVLVVIFRHDIKVILFKWDIDLR--A-EEQSKDRPFDVFVSYSSLDGEW
VRQKLLPMLERNPPFRTCFHERDFLPGAPIAENIMRAIQASKRTLMVVSKNFIASDWCE
FEFLTAHKSFMETKQNKIIVVMLEDVDTKSMDPMLRAYFTTKTFIRAAD------LFKEKL
YYAMPR-TE

>Pva-TLR1

---L-----------------DLSHNSLGYMESS
VFQNLSGLKFLNISKNKFKCDCDLRPFRDLLHEA--TFHF---GQNPCYYPTSLKKQL
VSNYSLSFIA-CDHEMIIVLAVAG-FIFLTIPIPALIAYYRLNLKYWWYFGRRRA-GYRPL-
DGGVHRYDAFVCYSKNELSWVVRELVEELENNERFQLCIHDRDFDLGGDIVDNIIRSID
CSRRVIFILSREFIRSYWGTFELNLALMEAIEKRINFIILIFFENIPKKEIPRHLQCFMRHVT
YASWPQQARAREMFWMKLKLALRNREE

>Pva-TLR2

YFANLTSLDHLILNGVYLRALNDDKPECLFQGLGKLKVLDLAFTHLKGLPERLFKDLTRL
ETLILRHNQLSGWNDVVLENLKNLRSLDVSGNQIHTINQSSLRPVSTLNHFQAFSNPYI
CDCNLAWYADWVRQMHATLKISYQVPYNCSN---LKNKSLLQYRPTFVE-
CHRLIILSASGFG-LFIVILAVIGLLYKYRWYIRYWIFLLRSRRARHIEENDRLL
YTYDCFITYSGEDSDLVTQQLLPKLENEFGYKMCIHERDFKLGRDISENIAESIEKSRKV
LVVLTQNFVQSEWCKFEVNLAHANTLHNARQSLIIILVEDVGFEHMTPILRYLIKKKTFLE
WSNEEQGQRVFWERLKDAIQQRGQ

> Pva-TLR3

YFAGLLTLISLKMSDLDMGKLS--DKVCLFEGLTELKYLDLHKVMLKNLPANIFVDLKSL

VYLRISGNKLLAINPVVFSSMLSLERLDVSSNLIQNINQSSLQPFKGLNYFEAYNNPYAC
TCDLQWYTDWLRQMKRKIIRKNMQYKCASPKKFKKKNLLTYNPTFID-CYEPFIIGTTV
GS-FAVLATIVVAVGYHYRWYIRYWLFLFRSRFAKNLREDERLVYRYDCFVTYCE-
DDGWVLETLRPKLEDEFGFRVCLQDRDFELGKSKVDNIDEAIQNSRKVLIFLTANFAMN
SWCNFELSLAHANCLENDQQHLIIIMMEDVSPKYMTPILRYLVRKRTYIEWTGDEVGQN
LFWQKLPDAIRSPNQ

> Pva-TLR4

YFSGRPSLVSLKMPGVNLHKQT—KSSCLFRGLFRLKHLDLHDVQLKRLPSDMF
QDLQSLVYLRLSGNMLHEINPVVFSML-SLARLDVSSNQIQNINQSSLQPFKGLNQF
EAHYNPYACTCDLQWYTEWLRTMIRKVTIRKYMQYKCATPKQIERKNLLTYDPTFLD-
CYEPFIIGTSVGS-FAVLVIIVVTVGYHYRWYIRYWLFLFRSRFAKNLREDERLVY
RYDCFVTYCE-DDGWVLETLRPKLEDEFGFRVCLQDRDFELGKSKVDNIDEAIQ
NSRKVLIFLTANFAMNSWCNFELSLAHANCLENDQQHLIIIMMEDVSPKYMTPILRYLVR
KRTYIEWTGDEVGQNLFWQKLPDAIRSPNQ

>Pva-TLR5

RYKIAPSLKKLILSGNKLYIMLRDQKKKHFKHLNNLTHIDLSHNSINELYPEVFSELSNV
KEIILRRNRLLAVTSMNITEMPSLKNVSLVANNIRVVPETSLRAWRG-KSFDFSRNPFNC
SCQFLPFLRWFNNVSSTVTLLHAENYRCGDEQKI---FVREVRLDELVKCTSAWIVISIA
LSICVALCITLGSILYRHRWTIRYYIVSAARRTRGHPPQ-MLRRFQYDAFVVYSSEDRHW
VHDAMRTRLEDGSDFGLCIHYRNFLPGRHIEESVIDAIENSRHSILVVSRNFLRSEWCIF
EMHMARNIFRQQRKDVLIVVLLEDIPVQEAPLTLVNLLRTRTYLKWPADDVGQEAFWE
MLKETLKKEPE

> Pva-TLR6

LLKGLGNLKWLFLNNNQLGKNLLQEYSLLFGDTLSLKELHLDRNDISSLPGNLFHSMKN
LEILSLRDNKISHWSPKLFAPLKSMEALDLSNNLIALINQTSVHNING--AFNLTGNPFAC
TCDLMWFRQWVNIT—NISFPCIGQYACNSPHSLQNTKFLDWYPDPRD-CINPFYVG
GSVCG-TVLLMLVISGATYRRRWFIRLSWYKLTHRRRGYRSLNNADVPSFDAFVSFCE
EDRQWVFDTLMKTFDEDNNFNICHDERDFPPNLSTAGCIFGCIENSRKFIVVVSEDYD
YCGRLEIELHYALQEIMEDAEFEIIVLLKDNPHPSRIPKHVAHLVSDPEFVEWPSDNDGQ
QLCLRRLQTMLERD--

>Ce_Toll1

-MVPVVELP-IILSGVTLPQLRGTSIPKAFHTLPALKTLDLSDNSLISLSGEEFLKCGEV
SQLFLNGNRFSTLSRGIFEKLPNLKYLTLHNNSLEDIPQVL----TALSKISLSSNPLRC
DCSGEHAAEWFSLHRH--LVVDFPKVECWENVTNMGNDVFVMP--IEELRDYSILFVIIT
ISIAVLLCVLVILAISFIRKSHDAINQRYKA--S--NCSTSGSSPLYHAFVSYSKKDEKM
VIDQLCRPLED-EDYQLCLLHRDGPTYCAISDELIAQMDSSQCLILVLTKHFLENEWKTL
QIKTSHQLFAKNRAKRVIAVLGDGVDANLLDDELGQILRKHTRIEMRS------LFWTLL
HSSLPSRLP

>Dm_Toll1

-LTHVP-LPNLHLENNTL----------------------------LRLPSANTPGYESVTSLHLAGN
NLTSIDVDQ---LTNLTHLDISWNHLQMLNATVLGFLMKWRSVKLSGNPWMC
DCTAKPLLLFTQDNFE--RIGDRNEMMCVNAEM--PTRMVEL---STNICPAVFIALAVV
IALTGLLAGFTAALYYKFQTEIKIWLYHNLLLW----EEDLDKDKKFDAFISYSHKDQSF
IEDYLVPQLEHPQKFQLCVHERDWLVGGHIPENIMRSVADSRRTIIVLSQNFIKSEWAR
LEFRAAHRSALNEGRSRIIVIIYSDIDVEKLDEELKAYLKMNTYLKWGD------WFWDKL
RFALPHRRP

>Dm_Toll2
-LAALPRIP-LYLDGNNMPELEASTLNGSLAQLVNLRVLHLENNKLTALEGTEFRSLGLL
RELYLHNNMLTHISNATFEPLVSLEVLRLDNNRLSSLP-------HSLQGLTLGRNAWSC
RCQQLRLAQFVSDNAM--VVRDAHDIYCLDAGI----K-RELELIANGDCS-YRLPLLAA
VL-VLIFLVVVLIIVFVFRESVRMWLFHYGVRVP-----FEDAGKLYDAIILHSEKDYEF
VCRNIAAELEHRPPFRLCIQQRDLPPQA-SHLQLVEGARASRKIILVLTRNLLATEWNRI
EFRNAFHESLRGLAQKLVIIEETSVSAEAEDAELSPYLKSVPSLLTCD------YFWEKL
RYAIPIESP
>Dm_Toll3
-LLQMPSLSS--s-----------------------RVTYVDLRNNNL
TALSQKNRSSINRL-KLHLLDNPWSCSCNDIEKINFMKSVSS--SIVDFTEIKC-SN----
GEKLVSIN--QHI-CP-SDLFYYLALAISLVATIIALNFLIWFRQPVLVWFYHGVCLSA----
RELDKDKRFDAFLAFTHKDEALL-EEFVDRLERRPRFQLCFYLRDWLAGESIPDCIG
QSIKDSRRIIVLMTENFMNSTWGRLEFRLALHATSRDRCKRLIVVLYPNVKNDSLDSEL
RTYMAFNTYLERSH------NFWNKLIYSMP----
>Dm_Toll4
-LSEIPQLPTLVFERNSLKKWP--------------------------------PGYSSV
TRFYLAHNRLSDIDQ------DKLEYLDISNNNFSALDDRVRGFLKRL-QLSLFGNPWTC
RCEDKDFLVFVKEQAK--NIANASAIQCIDT----GRSLIEVE--ETD-CP-SVLIYYTS
LAVSLLIIALSINVFICFRQPIMIWFYHEICLSA----RELDEDKKYDAFLSFTHKDEDL
I-EEFVDRLENRHKFRLCFYLRDWLVGESIPDCINQSVKGSRRIIILMTKNFLKSTWGRL
EFRLALHATSRDRCKRLIVVLYPDVEHDDLDSELRAYMVLNTYLDRNN------NFWNKL
MYSMPHASH
>Dm_Toll5
-LEELP-LPRLKVGNNSL----------------------------TSLPTSEHSGYANV
SGLFLSDNNLTSLGS---DQLPNLTHLDVRGNQIQSLSDEFLLFLNNTMTLSLSGNPITC
GCESLSLLFFVRTNPQ--RVRDIADIVCTKQKK----SFQQM---EAFLCP-SYLLISCV
VGGLVIVICLLTVFYLMFQQELKIWLYNNLCLW----EEELDKDKTYDAFISYSHKDEEL
I-SKLLPKLESPHPFRLCLHDRDWLVGDCIPEQIVRTVDDSKRVIIVLSQHFIDSVWARM
EFRIAYQATLQDKRKRIIIILYRELEHNGIDSELRAYLKLNTYLKWGD------LFWSKL
YYAMPHNRR
>Dm_Toll6
-YSEMPRVP-LYIDGNNFVELANSHINTTFSGLKRLLILHLEDNHIISLEGNEFHNLENL
RELYLQSNKIASIANGSFQMLRKLEVLRLDGNRLMHFEVWQL---PYLVEISLADNQWSC
ECGYLAFRNYLGQSSE--KIIDASRVSCIYNNA--SV-LRE----KNGKCT-GLLPLLLV
ATCAFVAFFGLIFGLFCYRHELKIWAHTNCLMNK---VDQLDKERPNDAYFAYSLQDEHF
VNQILAQTLENDIGYRLCLHYRDVNINAYITDALIEAAESAKQFVLVLSKNFLYNEWSRF
EYKSALHELVKR-RKRVVFILYGDLPQRDIDMDMRHYLRTSTCIEWDD------KFWQKL
RLALPLPNG
>Dm_Toll7
-TTELPRVP-VYLDGNNFPVLKGSAINRTFASLASLQLLHLADNKLRTLHGYEFEQLSAL
RELYLQNNQLTTIENATLAPLAALELIRIDGNRLVTLPIWQMHATTRLKSISLGRNQWSC
RCQFLQLTSYVADNAL--IVQDAQDIYCMAASSSGSLK-RELDFNATGACT-SYIPLLAA
AL-ALLFLLVVIAMVFAFRESLRIWLFHYGVRVP-----CEESEKLYDAVLLHSAKDSEF

VCQHLAAQLETRPPLRVCLQHRDLAHDA-THYQLLEATRVSRRVVILLTRNFLQ
TEWARCELRRSVHDALRGRPQKLVIIEEPEVAFEESDIELLPYLKTSAVIRRSD------
HFWEKLRYALPVDYP
>Dm_Toll8
-YEQLPHIP-LYLDGNNFRELQHSVLNRTFYGLLELEVLQLQSNQLKALNGN
EFQGLDNLQELYLQHNAIATIDTLTFTHLYHLKILRLDHNAITSFAVWNF---
SYLNELRLASNPWTCSCEFIDLRDYI-NRHE--YVVDKLKMKCISGNSPASLPV--V------
QCSNDYIPILVAILTAFIFVMICISLVFIFRQEMRVWCHRFGVRLN---VDKNEREKLFDA
FVSYSSKDELFVNEELAPMLEMEHRYKLCLHQRDFPVGGYLPETIVQAIDSSRRTIMVV
SENFIKSEWCRFEFKSAHQSVLRDRRRRLIVIVLGEVPQKELDPDLRLYLKTNTYLQWG
D------LFWQKLRFALPDVSS
>Dm_Toll9
AFDGIATLKYLYFERSNIKDLE--------KSLKNLQVLGLAGNNINALTPAMFQSLESL
EILDLSSNHVGNWYRSAFHN-SALRVLNLRSNTINMLSNEMLKDFERLDYLSLG
DNDFICDCHLLWYIPWLQRSYSKLRFEDYMVAKCSAPYHLDGDTLLDFQLQVDENCQ
SELHVTNTVIAVMLVGACILGFIIYLKRWHIHYYYSSLKSAAKKFTNIQRDPSAVYDIFISY
CQNDRTWVLNELLPNVEETGDVSICLHERDFQIGVTILDNIISCMDRSYSLMLIISSKFLL
SHWCQFEMYLAQHRIFEVSKEHLILVFLEDIPRRKRPKTLQYLMDVKTYIKWPTAKEDR
KLFWKRLKRSLERE--
>Ci_TLR1
AFKHV-NLTC--IKFNQV----EQ-NGIMFSGL-MVKQLYFIRSNIRSISSSAFTGSVHL
RLLDVSYNKITGLEKDIFTNL--LEELNLRGNQIRVLDPSTFSSLVNLRSLDIENNRFLC
NCDIIPLQQWIIDKLYRILL---RNVTCSLHSSRSYVDIIEW---DSELCWK--KIVGIV
LGC-LLLSTACAVFGFSVRFQALFWYEMIKSKV-SYHPRNRSDVYEYQAYISC
DSVDEAWVVRQLLCAIENETPMKLCFPSRDFKPGCPKMVSAANNLRLSKHALVILSKD
YVANSWTRFELSMVSEMWRNSERESLIVVYLKEV--ERL---PVLGVRRNAWLV
WPTDVADRPSFWMKLRRSLAK---
>Ci_TLR2
RFNVLPTIPRLDLSNLQL----NE--KISLTQLTRLTTLNLTGNKLTSIPL--QGLPRSI
ENINLSRNKISTLPATTLTCLPNLKQLDLRNNSFSTIQTQEVSIFLAVTSVLLKGNPLEC
NCKLRPLITWIQTNEKDLSTHDLKDLICFTPKRFEGRFIINLSES----CP-NLLIGGLV
TALLIIIIIIVNIYLYKKRKKQERRDI-------GFKDLEED-TYEYDAFVSYSSDDVEF
VYK-MLEEMEEKRERKMCIHERDFTPGRGIADNIVECISTSRRMVLVVSRKYASSA
WCQYEVQIALTELHAKRRRLLVPILLEDVTREQYAGSVTTILSAITAIQAPKAQRTWANF
WNKLDKTLT----
>Od_TLR
QLKNL-NLRGVDFSMNKITHFC----IDDFVNLEDLEFFNASLNQITDIPNNTFSFAREL
RVLDLHANSIQELN---FANLPELRMLDVSENQIRTSVDPYL---GALEQLDASYNPFQC
DCQLKKFVQFVQEPGRIVGIQAQSRYKCQIPRLLGNLNLLRL---QDKVCENEFYLSILA
IS-IVVILVVVAVVSKNRRQRMKMKELSGRNRVR---AAKN-NIVKNDAAILCHINSQKW
VTDVMLPTLKQKPQEKLYI---DFIKSQVKNEKLRRCVEQNKRVIIIITTEFASSDACLF
CLQAIYDLTRRNRKDGIVLVVLEPIPWNSMPHALKILMAEKTFIQYPVEDVGRQYFWDA
LRASIYQERT
>Sp_TLR020
VFRQLSVLQELNLEYCQIGNL-----PLVFSGLESLQKLSLKGNNIQHIHDDVLSGLGQV
NIIDFEGNQIIYLDELIFSNNRNLTNLSLADNKLTRFNQKTFKPISSISSLDLSMNPIDC

NCDLKWLIYWINKP---IHLIDRDKTICSSLEPFREKPLLDVDPNEL--CILGLLFL-IP
LASIGL--VVISVLLYHFRWQLRYKLFLLKLAA-GYKEMRDHNDYEFDVNIIFGEDDEEW
IREQLRPALGERLQ-RNVFGDEDLVLGMHYLDSVHYVVSHSYKTIIVLSRAAVQDRWFIL
KFRTAMDHVSDTLTEFVVVVFLEDIPDDEMPFLARLYLNDGRYIHWTEDARGQECFWD
ELTKNLT----
>Sp_TLR007
VFQNLSQLVYLDMTNSRIHTLR----SGLFSPLSSLRYLYIGENNLGEVPGDIFNGLFRL
NVLTFQNNILSSLDPKTFAQTLRLTDLYLPGNQISTIKPGTV---NTS-RFDISKNPFSC
TCSLAWFRQWLDSAD--IDFKHADQTLCSGLKGLSKQPILSFHPD---HCGVIFLIAGIS
FTGIFL--FFITLLAYNRRWWLNHKLFLLKLAV-GYKEMAEADNYEFHLNLMFLEEEEEW
VDRVMKPALEERFHQNIIYGDKDLHLGMFYINAINDALDNSFKTVLLISNQSIRDAWCMT
KLRMALEHLNETGLDKIILIFLEDIEDENLPYLVRLFMSRNKYMLWTDDEDGQELFWAQ
FEKSMRAN--
>Sp_TLR053
IFTPLRNLVELDLTSCCIKQVA----SRTFANLTTLLQLSLQDNDLTSIPKDAFQGLQNL
QVLRLQNNLIKFIHQGLFMGTNELEQLYLQNNHISTVASNTF---SSL-RFNIAYNPLTC
DCQLAWFRQWLNEVEGKIDLAPKNQTRCSSLKVLVNQIIWSFHPD---YCGITMIIVSAC
FAPILV--LTLGILVYLNRWWINYKLYLLKLAI-GYHEITEPEDYEFQLNLMFHDDDEWW
VNDCMKPFLEQRMHERVIFGDADLHPGSFYLNAIYDVIENSHKTILLLSNQSVDDTWYM
TKLRMTVEHMNDTKLEKVILIFLEDIDDDHLPYLVRLLLSRNKYLLWTEDEEGQEVFWA
KVQKSMRQN--
>Sp_TLR039
ILTDLLLLQELDLSDCQLTEI-----VNAFEGLQSLQILHLEGNQLLDLPHGVLWNMAHL
RNVYLEGNKLKYLDRDLFFNSSRLRNLTLARNQLTGLNHSTFKPIKTLLSIDISENEITC
TCNLKWLPIWLSGS---ITLLNEIDTRCSSLEELELKPLMSFKPAEL--CGPIALYCSLP
IVTTWI--IIVLVFAYRHRWFLKYKLFLLKMAV-GYREIRDFDDYEFHLNVMFAEEDEGW
VRYRLRPVLEELLE-RNVYGDNDLPLGMHYYDAVHYVVEKSYKTIVLVSRAAIQDNWFII
QFRTAADQVNDTQIENMVVIFLEDIPDVELPFLVRLYLSDRKYLSWKEDERFQEYFWQ
KLIKMLKRN--
>Sp_TLR056
IFQNLSNLQILRLDKCSLSVL-----IGIFIDLKSLVSLHLENNHLKVISTGLFDKLYDL
QYLLLNGNELTYLDSNLFKYLSSLRCLDASENRISGLNHSTIEPL-RLTTLGLSLNPLVC
NCNLKWLPGWLKGT---IELIDSMGTTCNTLEPFRGKQLITFDPRYE--CGPITLYSCLA
MIGFVL--IFAVGLIYYQRWWVRYQLFLLKLCF-GYEEVHDRGEFQYDIAIMLDEIDNEW
VNQHLRPALMERGD-RIVCGDEELMLGMFYLDAVHYATEKSFKTIFVISHAA
LQDQWFMMKFRTVLDHVNDVGTEKMILVFVEDVEDDELPFLIRLFLSDHRYLVWPDDE
RGQEYFWEELIRDLTRH--
>Sp_TLR044
TFQGLQNLQNLEMDNSDITSLN----EDIFLNLTSLQHLSIDVNHIAELTSRHLADLRSL
VGVSIKSNEIKGLASDVFTNNPHLSYLYISHNHLTTVKEGTV-----LRTLDVSNNPFSC
NCEFTWFLNWINKA--EVSIIHPDQTNCSSLAPFKNQPILAFDPT---VCGPVWVYIITI
--FVIVTCIMICVVAYQRRWLINYKLFHLKILL-GRRDDHDR-DYEYDINLAFDDDDEQW
VRGILKPGLEERLDDRIVCGDDDLPLGMYYIEAITEVFEQSYKSILIVSNRAVDNHSFIS
KLRLAVDQMNEVELEKVILIFKEDIPDGRLPYLVRLFLSKNKYFRWSEDKYGQKIMWEK
LVRELGKD--
>Sp_TLR016

VFNNLSALQVLNMSDCQISTI-----SGAFASMTSLTILSLQNNDLQILPLHIFDNLIHL
SIFSIGNNVLVYIDEALFAKMQMITSIDLARNQLSTFNQTTFSQITTLSSIDLSQNPIEC
SCKSKWLIKLLRGA---IDVQNGKDTTCSFMKPFGGEALESIQPNDL--CTAFPVYFSAV
FFAVIF--VIFIIFVYHFRWQLRYKHFLLRLAI-GYREILDREDYDFDVYVISTDDDENW
IHDQLKPSFQRFLYSRNVFTEDDLPLGMHRTEAVDHVLTRSFKILVLVNKAACADDWFL
TCFRMAMDQVADTQTENIIVVFLENIEEDEMPLNVRLYMGGQGYVEWVEDDEGQKYF
WKRLEKCLSKH--
>Sp_TLR100
TFSSLGNLGTMYLQHNNLYNMWETIHVPFLKSLRRLKYLNLCYNGFQNIPNNSLSNLP
ELKALFLCHNKISHLQDNIINDL-PLTTLDLGHNQINLINQTLLEPLGTLKALTVSGNPFSC
GCDLQWFREWLDVT--QVHVDDNSHMICSSPPDMRGKLVIDFHPETLN-
CDHTWVLVGVG----SCMVFVTVALAVKFRFHINYCFNLVNARRRKYQRIKEDLPFLY
DAFVFFSHKDEEWVYNELVRHLEDDSGLRLCVHNRDFTLGRKILDNTIEAVDSSRFTLC
ILSADYLDSHWCKMEQEFAMANLIDR—DVLIIIALGEIPENKITKKLHKVMMKRTYLKW
PMEPVQRNDFWMKLKTVLREPNN
>Hs_TLR1
----TKSLLSLNMSSNIL--------DTIFRCLPRIKVLDLHSNKIKSIPK--VVKLEAL
QELNVAFNSLTDLPG—CGSFSSLSVLIIDHNSVSHPSADFFQSCQKMRSIKAGD
NPFQCTCELGEFVKNIDQV--SSEVEGWDSYKCDYPESYRGTLLKDFHMSELS-CN-
TLLIVTIVATMLVLVTVTSLCSYLDLPWYLRMVCQWTQTRRRARNPLEEQRNLQFHAFI
SYSGHDSFWVKNELLPNLEKE-GMQICLHERNFVPGKSIVENIITCIEKSYKSIFVLSP
NFVQSEWCHYELYFAHHNLFHEGSNSLILILLEPIPQYSIPSKLKSLMARRTYLEWPKEK
SKRGLFWANLRAAINKK--
>Hs_TLR2
----WPKMKYLNLSSTRIHSVT--------GCIPTLEILDVSNNNL-----NLSLNLPQL
KELYISRNKLMTLPDASL--LPMLLVLKISRNAITTFSKEQLDSFHTLKTLEAGGNNFIC
SCEFLSFTQEQQALA-KVLIDWPANYLCDSPSHVRGQQVQDVRLSVSE-
CHRTALVSGMCCA-LFLLILLTGVLCHRFHWYMKMMWAWLQAKRKPRK--
APSRNICYDAFVSYSERDAYWVENLMVQELENNPPFKLCLHKRDFIPGKWIIDNIIDSIE
KSHKTVFVLSENFVKSEWCKYELDFSHFRLFDENNDAAILILLEPIEKKAIPQKLRKIMNT
KTYLEWPMDEAQREGFWVNLRAAIKS---
>Hs_TLR3
MLEGLEKLEILDLQHNNLARLWHANPGPIYKGLSHLHILNLESNGFDEIPVEVFKDLFEL
KIIDLGLNNLNTLPASVFNNQVSLKSLNLQKNLITSVEKKVFPAFRNLTELDMRFNPFDC
TCESIWFVNWINET--HTNIPELSHYLCNTPPHYHGFPVRLFDTS---SCKDPFELFFMI
TS-ILLIFIFIVLLIHFEGWRISFYWNVSVHRV-GFKEIDQTEQFEYAAYIIHAYKDKDW
VWEHFSSMEKEDQSLKFCLEERDFEAGVFELEAIVNSIKRSRKIIFVITHHLLKDPLCRF
KVHHAVQQAIEQNLDSIILVFLEEIPDYKLNHARRGMFKSHCILNWPVQKERIGAFRHKL
QVALGSKNS
>Hs_TLR4
IFNGLSSLEVLKMAGNSF----ENFLPDIFTELRNLTFLDLSQCQLEQLSPTAFNSLSSL
QVLNMSHNNFFSLDTFPYKCLNSLQVLDYSLNHIMTSKKQELQHFSSLAFLNLTQNDF
ACTCEHQSFLQWIKDQ--RQLLVEVERMECATPSDKQGMPVLSLNIT----
CQMKTIIGVSVLS--VLVVSVVAVLVYKFYFHLMLLAG-------KYG----
RGENIYDAFVIYSSQDEDWVRNELVKNLEEGPPFQLCLHYRDFIPGVAIAANIHEGFHK

SRKVIVVVSQHFIQSRWCIFEYEIAQTWQFLSSRAGIIFIVLQKVEKTLLRQELYRLLSRN
TYLEWEDSVLGRHIFWRRLRKALLDEQE
>Hs_TLR5
-----PSLEQLFLGENMLQLAWTELCWDVFEGLSHLQVLYLNHNYLNSLPPGVFSHLTAL
RGLSLNSNRLTVLSH---NDLPNLEILDISRNQLLAPNPDVF---VSLSVLDITHNKFIC
ECELSTFINWLNHT--NVTIAGPADIYCVYPDSFSGVSL--FSLSTEG-CDEKFFIVCTV
TLT---LFLMTILTVTKFRGFCFICYKAQRLVFKDHPQGTEPDMYKYDAYLCFSSKDFTW
VQNALLKHLDDQNRFNLCFEERDFVPGENRIANIQDAIWNSRKIVCLVSRHFLRDGWC
LEAFSYAQGRCLSDLNSALIMVVVGSLSQYQLHQSIRGFVQKQQYLRWPEDLQDVGW
FLHKLSQQILKKQT
>Hs_TLR6
----VESIVVLNLSSNML--------DSVFRCLPRIKVLDLHSNKIKSVPK--VVKLEAL
QELNVAFNSLTDLPG--CGSFSSLSVLIIDHNSVSHPSADFFQSCQKMRSIKAGD
NPFQCTCELREFVKNIDQV--SSEVEGWDSYKCDYPESYRGSPLKDFHMSELS-CN-
TLLIVTIGATMLVLVTVTSLCIYLDLPWYLRMVCQWTQTRRRARNPLEEQRNLQFHAFIS
YSEHDSAWVKSELVPYLEKE-DIQICLHERNFVPGKSIVENIINCIEKSYKSIFV
LSPNFVQSEWCHYELYFAHHNLFHEGSNNLILILLEPIPQNSIPNKLKALMTQRTYLQW
PKEKSKRGLFWANIRAAFNKEKS
>Hs_TLR7
VFDGMPNLKNLSLAKNGLKSF-----WKKLQCLKNLETLDLSHNQLTTVPERLSNCSRSL
KNLILKNNQIRSLTKYFLQDAFQLRYLDLSSNKIQMIQKTSFNVLNNLKMLLLHHNRFLC
TCDAVWFVWWVNHT--EVTIPYLTDVTCVGPGAHKGQSVISLDLYT---CELLILFSLSI
SVSLFL--MVMMTASHLYFWDVWYIYHFCKAKIKGYQRLISPDCC-YDAFIVYDTKDPEW
VLAELVAKLEREKHFNLCLEERDWLPGQPVLENLSQSIQLSKKTVFVMTDKYAKTENF
KIAFYLSHQRLMDEKVDVIILIFLEKPFQKSKFLQLRKRLCGSSVLEWPTNPQAHPYFW
QCLKNALATDNH
>Hs_TLR8
AFLNLPSLTELHINDNMLKFF-----WTLLQQFPRLELLDLRGNKLLFLTDSLSDFTSSL
RTLLLSHNRISHLPSGFLSEVSSLKHLDLSSNLLKTINKSALKTTTKLSMLELHGNPFEC
TCDIGDFRRWMDHL--NVKIPRLVDVICASPGDQRGKSIVSLELTT---CVSVILFFFTF
FITTMV--MLAALAHHLFYWDVWFIYNVCLAKVKGYRSLSTSQTF-YDAYISYDTKDADW
VINELRYHLERDKNVLLCLEERDWDPGLAIIDNLMQSINQSKKTVFVLTKKYAKSWNFK
TAFYLALQRLMDENMDVIIFILLEPVLQHSQYLRLRQRICKSSILQWPDNPKAEGLFWQT
LRNVVLTEND
>Hs_TLR9
TLRNLPSLQVLRLRDNYLAFF-----WWSLHFLPKLEVLDLAGNQLKALTNGSLPAGTRL
RRLDVSCNSISFVAPGFFSKAKELRELNLSANALKTVDHSWFPLASALQILDVSANPLH
CACGAA-FMDFLLEV--QAAVPGLSRVKCGSPGQLQGLSIFAQDLRL---CLDWDC
FALSLLAVALG—LGVPMLHHLCGWDLWYCFHLCLAWLRGRQSGRDEDALPYDA
FVVFDKTQSDWVYNELRGQLERGRALRLCLEERDWLPGKTLFENLWASVYGSRKTLF
VLAHTDRVSGLLRASFLLAQQRLLEDRKDVVVLVILSPDGRRSRYVRLRQRLCRQSVL
LWPHQPSGQRSFWAQLGMALTRDNH
>Hs_TLR10
-----PTVVNMNLSYNKL--------DSVFRCLPSIQILDLNNNQIQTVPK--TIHLMAL
RELNIAFNFLTDLPG--CSHFSRLSVLNIEMNFILSPSLDFVQSCQEVKTLNAGRNPFRC
TCELKNFIQLETYS--EVMMVGWDSYTCEYPLNLRGTRLKDVHLHELS-CNTALLIVTIV

VIMLVLLAVAFCCLHFDLPWYLRMLGQCTQTWHRVRKTQEQKRNVRFHAFISYSEHD
SLWVKNELIPNLEKEGSILICLYESYFDPGKSISENIVSFIEKSYKSIFVLSPNFVQNEWC
HYEFYFAHHNLFHENSDHIILILLEPIPFYCIPTKLKALLEKKAYLEWPKDRRKCGLFWAN
LRAAINNEQT
>Nv_TLR
-LHRLPKMP-VNLRGNAIRELP--------------------------------HYLGNI
TVLELSNNEIKELNMTFVDSLARVVNLAINDNKLKYLPRGVTNLTEGFRSLSISHNFFVC
DCYASWMRDWLANNTD--KIEDTSSILCASG--LEGLPIISVP--LSDNCSALSLLLAIV
LAVLLVLSVVAFVMTYCFRWEMKILMYHFNWHPR--D--DTDVSKIYDTFISYSSQDASW
VRETLQRTLESVPPYRLCIHDRDFEIGASIHDNILNSVRLSKRMIMVLSNHFIASEWCRL
EFRAAHQKVLEDRTNYLIIILFDDVDPSTLDDETKLYLRTNTYLSVSN------WFWQKL
FYALPKPLA
>Ad_TLR1
-LVAMPSVP-MFLQSNNIREIP--------------------------------GYLENV
TSLYLSHNQIQRLDEKTIDRLKRIETLFIDSNKLTTLPRNIENVS--FTKISLQHNFFRC
DCETKWMKQWLLREEA--HVDNIENILCHSD--VQGKAISRLP--DEELCLEAFKITAYT
LGGLLFVSLVAFAVGYKFRSEAKVFMYHFNWHPR--N--
DLDPNKPYDAFISFSGNDYEW
ICNTLCVRLENDPPYKLCLHHRDFLVGAPIQQNIFDGIERSKRMIMTLSKHFVRSEWCLL
EFRAAHQKVLEDRINYLIIILFDDVDMAEVDDEIKLYMRTNTYLSVKN------WFWEKL
FYALPQNTN
>Ad_TLR2
----MPSVP-LFLQSNKIEEIP--------------------------------SYLENV
TALYLSHNNIERLNEKTIDRLKRIEILFIDSNKLTTLPRNIENVS--FIKISLQHNFFRC
DCKTKWMKHWLLRQEA--HIDNIENILCHSD--VKGKAISRLP--DEEVCPAAFKITAYT
LGGLLLVFLVAFAVGYKFRGEVKVFMYHFNWHPR--N--DLDPNKIYDAFISFSGIDYEW
ISNTLCARLENDPPYKLCLHHRDFLVGAPIQQNIFNGIEKSKRMIMILSKNFVKSEWCLL
EFRAAHQKVLEDRINYLIIILFDDVDMAEVDDEIKLYMRTNTYLSIKN------WFWEKL
FYALPQNSK
>Ad_TLR3
-LVAMPSVP-LLLQSNNIREIP--------------------------------GYLENV
TQLYLSHNNIERLDEKTIDRLKRIEKLFIDSNKLTTLPRNIENVS--FTKIALQHNLFRC
DCKTKWIKHWLSRQED--HIEHIDNILCHSD--VKDKVISNLP--DEEVCLEAFKITACT
LGGLLFMFLLAFAVGYKFRSEGKVFMYHFNWHPR--N--
DSNPNKTYDAFISFSGNDYAW
ISNTLCARLENDPPYNLCLHHRDFLVGAPIQQNIFNAIEKSKRMIMILSKNFVKSEWCLL
EFRAAHQKVLEDRINYLIIILFDDVDMAEVDDEIKLYMRTNTYLSVKN------WFWEKL
FYALPQNSN
>Ad_TLR4
-LVTMPSVP-LFLQSNDIQEIP--------------------------------RYLENV
TSLYLSHNQIERLDEKTIDRLKRIQVLFIDSNKLTTLPRNIENVS--FTKISMQHNFFRC
DCKTKWMKQWLLREEA--HVDNIENILCHSN--VQGKAISRLP--DEKVCLEAFKITAYT
LGCLFLVFLVAFAVGYKFRSEAKVFMYHFNWHPR--K--
DSDPNKIYDAFVSFSGNDYEW
ISNTLCVRLENDPPYKLCLHHRDFLVGAPIQQNIFNGIEKSKRMIMILSKNFVRSEWCLL
EFRAAHQKVLEDRINYLIIILFDDVDMAEVDDEIKLYMRTNTYLSVKN------WFWEKL

FYALPQNSN
>Am_TLR
-FVAMPSLP-LFLQSNNIREIP--------------------------------GYLENV
TSLYLSHNQIERLDEKTIDRLKRIQVLFIDSNKLTTLPRNIENVS--FTKISLQHNFFRC
DCKTKWMKQWLLREEA--HVDNIENILCHSD--VQGKAISRLP--DEEVCLDAFKITAYT
LGGLLLVFLVAFAVGYKFRGEVKVFMYHFNWHPR--N--DLDPNKPYDAFISFSGIDYEW
ISNTLCVRLENDPPYKLCLHHRDFLVGAPIQQNIFDGIERSKRMIMILSKHFVKSEWCLL
EFRAAHQKVLEDRINYLIIILFDDVDMAEVDDEIKLYMRTNTYLSVKN------WFWEKL
FYALPQNSN
>Of_TLR
-LTAIPKVP-LKLEDNNIREIP--------------------------------PYMENV
TALYLTHNKIQVLNKSTVRRFTRIKVLFIDSNKLTYLPKNIENLN--FTSLALHHNFFKC
DCTTLWIKHWLQRKQS--KILHIKNVLCNSE--TQGKAIYTLP--NEEVCKKTFKIIALT
LGGALVLTFIAFIVAYKYRGEMKVLMYHFNWHPR--D--DSDPRKIYDAFVSYSGSDHQW
VVNTLQERLEHDPPYKLCIHHRDFVVGAPIQENILNSVDQSKRMLMVLSRNFLKSEWC
LLEFRAAHRKVLEDRMNYLIIILFDGINMDELDDEMKLYMRTNTYLSVSY------WFWEKL
YYAMPQSTD
>Mm_MyD88
----NPTV----------------------ADWTLLAEE--------------FEY-LEI
RELETRPDPTSLLDAASVGRL-ELALLDRE-DILKE-----------LK--RIEEDC---
-------QKYLKQQNQ-----------------SE--KLQV-RVE-SVQ-----------
LG-G----ITT--------------------------DD-LGTPELFDAFICYCPNDIEF
V-
QEMIRQLEQDYRLKLCVSDRDVLPGTCVWSIASELIKRCRRMVVVVSDDYLQSKECDF
QTKFALSLSPGVQQKRLIPIKYKAMKK-----DFPSILRFITICDYTN--CTKSWFWTRL
AKALSLP--
>Dm_MyD88
------KLRS----------------EEGYRDWRGISEL-------QKVDE---------
-----ANNPMLVLISQTVGHLEHLGIIDIQENLAKDTQ----RFI--MKAL------EAC
-C----FNNY--SSSNNITV---QSVQ-----------I--L---DEDRCV---------
MG--------------------------------------Q-PLPRYNACVLYAEADIDH
A-
TEIMNNLESRYNLRLFLRHRDMLMGVPFEHVQSHFMTRCNHLIVVLTEEFLRSPENTY
LVNFTQKIQIENHTRKIIPILYKDM-------HIPQTLGIYTHIKYAGDS----NFWDKL
ARSLHRQPS
>Bg_MyD88
----DIDVMH----------------N-GYPNYGGLAEL--------------FSG-LEI
MEFERAKSPTALLLEPKIGKL-ELFQLDRI-DVLTD-----------CH--AINDEA---
-------KLFIKESKA-----------------LL--KIQD-TVTPGSTPPQ--------
L---------------------------------------TGKKTYYDAYICYNPKDLEF
V-RELISRFESKYRFTLFVPFRDDLPGMNEHAINAKIIERCRHMIVVLSKNFLQSEACEF
QSTFAQSLSPGARNKRIVPIKIEDC-------VIPNILRIMACCDFTK--DLWDWSWDRL
ARSITAQQS
>La_MyD88
----NPSVLT----------------DSGFKDWKGLAEL--------------FMN-DDI
SNFESMRDSTLILKDSMIATL-DIQQLERY-DILEDPT---------IK--QVENNV---

-------KSYLRQSKE-----------M-----IE--KLQD-EVSMEYD--E--------
YK-Y----MTT--------------------------DV-TGKAMYYDAFVSYAEEDIGF
V-KKLISELEKEYGLKLCVQARDLIPGASTNTVCAKLIERCTRMVIILSPKFLNSAQCDF
QIKFAQSLSPGSRGKKLVPIMYKCC-------DIPSILRHIAICDYTK--DLQEWFWNRL
AMSLKAP--
>Aq_MyD88
-----QNL----------------------SDWRTLVEL-------------GFTY-EMV
MLLRAKNSPTSLLEEATLESL--IVMMELENV----------SALQQVK--SIH-D----
RPPP--RK---PNSCS----SDPSTMVA--P-AVPSLKYVDVNLQTNSDCEQ--------
LSS-----LSL----------------------------DHGDGSDFDIFLSFAPADAEF
A-DEMRLRLIN-AGISVYIASEGLMPGQSFIDEVADKIRGCRKTIIILSPDYNQCSWCNY
EARLAHHKNPDPKRHTLIPIVYRKC-------EVPDFMSHLFYLDFSRDHQCEKYFWDRL
YKSVRHQ--
>Lp_MyD88
----NPQLTT----------------LTGLRDFRGVAEL--------------FDY-WDI
QNFQMTSDPFKLLIQNTVGKL-DLEEIERT-DVIDD-----------VK--YAEIDA---
-------HNYLRKQKW-----------C-----KE--SLQD-NVS---SCTA--------
--------LTV--------------------------DVNEGEVSLYDAYVCYTDEDIEC
V-HILSRQLESE-GIRLFIRDRDLLLGQMEYEAFARLIERCNRVLIVLSPEFLKSVECEF
QTRYATSLAVEQQQRKLIPIIYRSC-------DVPHLLRYLSKIDFTK--HIHDWVWHRL
IHSIKGERE
>Spi_MyD88
----RPCL----------------------NDYRLLAAK--------------YTN-EEI
KYLESLREPVELMTRRTIAEL-SLQQIDRP-DVVQD-----------LQ--YI--DS---
-------TPFEKEREQ--
--------RTN--------------------------NVQRTVRKSYHAFVCFAEEDKAF
V-DNLVKKMENNRNLHLCLPVRDFLPVGSHLETTALAIQRCKKFIVILSKNYDSSQGAIY
QAQIATSLAPGAKEKRIIPVLIDEC-------SIPRTLSHITYLDYLR--DEKH-FWNLL
CDTLTRN--
>Spu_MyD88
----RPRG----------------------CDWRDMAEE--------------FSYQLHI
QNFALESDPVKVLSASNVGKL-DIEKIERH-DVLHE-----------LP--FLEEDC---
-------KRW-KRTQA-----------------AR--DIQV-EVT-NFS--S--------
LR-G----ITL--------------------------DS-SGPPEMFDAYVCFAMADLEF
V-QQLRSQLESPHNYKLCIDQRDLLPGGSHALVTAEIINRCNKMLVILSPEFLQSPSCDF
QTKFAVSLEPGAMKRRIIPILVKPC-------DLPLIIRHITLCDFTK--DLRPWFWGRL
RKAMSIR--
>Cg_MyD88
----DPGVTG----------------D--YNDYQGLAEV--------------FTF-QDI
TNFQRQSKPTEMLYQPTVDNL-KLQPIGRSDDVITE-----------CA--LIKKDV---
-------DRYCKDITG-----------------SK--DIQD-SVSPNRSCDS--------
LG-L----VTI--------------------------DVEKGDTLYYDAFVIYNPKDLEF
V-
KELAGKMEAPYNLKFCIPWRDDLPGGSRYEVSAHMITRCRRTLVILSSDFLKSAAADF
QLKFAHCLSPGARSKKVVPVFSAPC-------KMPGILRAVSFVDFTN--GLRDWNWPRL
NAVLRCPRD
