# Supplementary material for: The evolution of the metazoan Toll receptor family and its expression during protostome development
Source: BMC Ecol Evol. 2021 Nov 22;21:208. doi: 10.1186/s12862-021-01927-1 (PMC8609888; doi:10.1186/s12862-021-01927-1)

Additional file 3: Fig. S2 - Second phylogenetic analysis, excluding the 150-200 aminoacid region. Parameters applied for the construction of this phylogenetic tree are the same than the ones applied for the main phylogenetic analysis (Figure 4A). Bootstrap values are indicated next to the main nodes and all nodes with bootstrap values >60 are marked with full black dots.

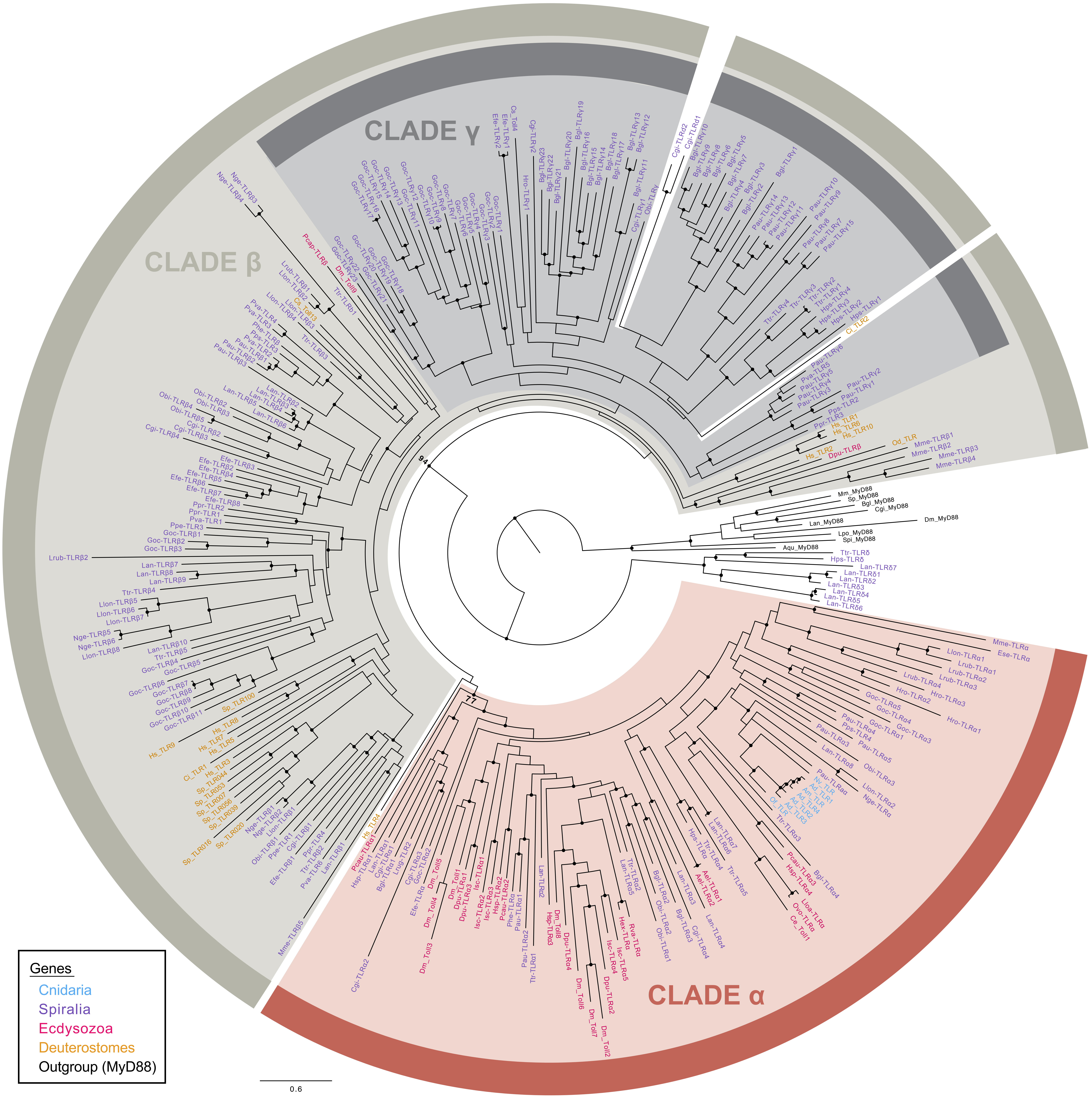

Supplement: Supplementary file 3 — Additional file 3: Fig. S2. Second phylogenetic analysis, excluding the 150–200 aminoacid region. Parameters applied for the construction of this phylogenetic tree are the same than the ones applied for the main phylogenetic analysis (Fig. 4A). Bootstrap values are indicated next to the main nodes and all nodes with bootstrap values >60 are marked with full black dots. [file 12862_2021_1927_MOESM3_ESM.pdf]
