# Supplementary material for: The evolution of the metazoan Toll receptor family and its expression during protostome development
Source: BMC Ecol Evol. 2021 Nov 22;21:208. doi: 10.1186/s12862-021-01927-1 (PMC8609888; doi:10.1186/s12862-021-01927-1)
Supplement: Supplementary file 4 — Additional file 4: Table S2. Species included in our study. [file 12862_2021_1927_MOESM4_ESM.pdf]

Additional file 4: Table S2 - Species included in our study.

	Species	TLR Source	Publication	Genome/transcriptome NCBI Accession number	Complete BUSCO gene values for transcriptomes
**Cnidaria**	*N. vectensis*	Literature	[1]	-	-
	A. digitifera	Literature	[2]	-	-
	A. millepora	Literature	[2]	-	-
	O. faveolata	Literature	[3]	-	-
**Xenacoelomorpha**	*X. profunda*	This study: Genome	Unpublished		-
	H. miamia	This study: Genome	-	GCA004352715	-
	P. naikaiensis	This study: Genome	-	PRJDB7329	-
	I. pulchra	This study: Genome	Unpublished		-
	M. stichopi	This study: Genome	Unpublished		-
	C. macropyga	This study: Transcriptome	[4]	SRX1343815	89.2%
**Bryozoa**	*M. membranacea*	This study: Transcriptome	-	SRX1121923	96.9%
	B. neritina	This study: Transcriptome	[5]		96.6%
**Cycliophora**	*S. pandora*	This study: Transcriptome	[6]	SRX1531719	87.4
**Annelida**	*G. oculata*	This study: Transcriptome.	Unpublished		99%
	E. fetida	This study: Transcriptome	-	SRX3108745	96.2%
	H. robusta	This study: Genome	[7]	AMQM00000000.1	-
	P. prolifica	Literature	[8]	-	-
**Mollusca**	*C. gigas*	This study: Genome	[9]	AFTI00000000	-
	O. bimaculoides	This study: Genome	[10]	PRJNA270931	-
	C. sinensis	Literature	[11]	-	-
	L. rugatus	Literature	[8]	-	-
	B. glabrata	This study: Genome	[12]	APKA00000000.1	-

	B. glabrata	Individual sequence(s) downloaded from NCBI	-	-	-
**Brachiopoda**	*T. transversa*	This study: Transcriptome.	[4]	SRX1307070	95.7%
	H. psittacea	This study: Transcriptome.	[8]	SRX731469	94.5%
	L. anatina	This study: Genome	[13]	LFEI00000000	-
**Micrognathozoa**	*L. maerski*	This study: Transcriptome.		SRX1121929	93.8%
**Gastrotricha**	*L. squamata*	This study: Transcriptome.	[14]	SRX1000997	89.6%.
	Macrodasys sp	This study: Transcriptome.	[15]	SRX534826	75.9%
	Megadasys sp	This study: Transcriptome.	[15]	SRX534835	70%
	D. aspetos	This study: Transcriptome.		SRX1121926	90%
	M. laticaudatus	This study: Transcriptome.		SRX872416	82.5%
**Nemertea**	*Lineus longissimus*	This study: Transcriptome.	[4]	SRX1343823	95.2%
	Lineus ruber	This study: Transcriptome.	Unpublished		95%
	N. geniculatus	This study: Genome	[16]	NMRB00000000	-
	P. peregrina	Literature	[8]	-	-
**Phoronida**	*P. harmeri*	This study: Transcriptome.		SRX1121914	90.4%
	P. australis	This study: Genome	[16]	NMRA00000000	-
	P. psammophila	Literature	[8]	-	-
	P. vancouverensis	Literature	[8]	-	-
**Platyhelminthes**	*M. lignano*	This study: Genome	[17]	SRP059553	-
	E. multilocularis	This study: Genome	[18]	PRJEB122	-
	H. microstoma	This study: Genome	[18]	PRJEB124	-
	S. mansoni	Literature	[19]	-	-
	S. mediterranea	Literature	[20]	-	-
**Rotifera**	*E. senta*	This study: Transcriptome.	Unpublished		95.2%

	R. tardigrada	This study: Transcriptome.	[21]	SRX1253177	91.1%
	E. gadi	This study: Transcriptome.		SRX1121912	74.9%
	M. hirudinaceus	This study: Transcriptome.	[15]	PRJEB5803	84.5%.
	A. vaga	Literature	[22]	-	-
**Priapulida**	*P. caudatus*	This study: Transcriptome.	[4]	SRX507009	93.9%
	H. spinulosus	This study: Transcriptome	[4]	SRX1343820	96.4%
**Tardigrada**	*H. exemplaris*	This study: Genome	[23]	SRX2495681	-
	R. varieornatus	This study: Genome	[24]	DRX012456	-
**Onychophora**	*P. capensis*	This study: Transcriptome.	[25]	SRX451023	62%
**Nematoda**	*L. loa*	This study: Genome	[26]	ADBU00000000.2	-
	O. volvulus	This study. Genome	[27]	CBVM000000000	-
	C. elegans	Individual sequence(s) downloaded from NCBI	-	-	-
**Loricifera**	*A. elegans*	This study: Transcriptome.		SRX1120677	36.2%
**Arthropoda**	*D. pulex*	This study: Genome	[28]	ACJG00000000	-
	D. melanogaster	Individual sequence(s) downloaded from NCBI	-	-	-
	I. scapularis	Literature	[29]	-	-
**Tunicata**	*C. intestinalis*	Literature	[30]	-	-
	O. dioika	Literature	[31]	-	-
**Echinodermata**	*S. purpuratus*	Literature	[32]	-	-
**Craniata**	*H. sapiens*	Individual sequence(s) downloaded from NCBI	-	-	-

Bibliography

1. Brennan JJ, Messerschmidt JL, Williams LM, Matthews BJ, Reynoso M, Gilmore TD. Sea anemone model has a single Toll-like receptor that can function in pathogen detection, NF-κB signal transduction, and development. Proc Natl Acad Sci. 2017;114:E10122–31. doi:10.1073/pnas.1711530114.
2. Poole AZ, Weis VM. TIR-domain-containing protein repertoire of nine anthozoan species reveals coral–specific expansions and uncharacterized proteins. Dev Comp Immunol. 2014;46:480–8. doi:10.1016/j.dci.2014.06.002.
3. Williams LM, Fuess LE, Brennan JJ, Mansfield KM, Salas-Rodriguez E, Welsh J, et al. A conserved Toll-like receptor-to-NF-κB signaling pathway in the endangered coral *Orbicella faveolata*. Dev Comp Immunol. 2018;79:128–36. doi:10.1016/j.dci.2017.10.016.
4. Cannon JT, Vellutini BC, Smith J, Ronquist F, Jondelius U, Hejnol A. Xenacoelomorpha is the sister group to Nephrozoa. Nature. 2016;530:89–93. doi:10.1038/nature16520.
5. Wong YH, Ryu T, Seridi L, Ghosheh Y, Bougouffa S, Qian P-Y, et al. Transcriptome analysis elucidates key developmental components of bryozoan lophophore development. Sci Rep. 2015;4:6534. doi:10.1038/srep06534.
6. Neves RC, Guimaraes JC, Strempel S, Reichert H. Transcriptome profiling of *Symbion pandora* (phylum Cycliophora): insights from a differential gene expression analysis. Org Divers Evol. 2017;17:111–9. doi:10.1007/s13127-016-0315-1.
7. Simakov O, Marletaz F, Cho S-J, Edsinger-Gonzales E, Havlak P, Hellsten U, et al. Insights into bilaterian evolution from three spiralian genomes. Nature. 2013;493:526–31. doi:10.1038/nature11696.
8. Halanych KM, Kocot KM. Repurposed transcriptomic data facilitate discovery of innate immunity *Toll-Like Receptor (TLR)* genes across Lophotrochozoa. Biol Bull. 2014;227:201–9. doi:10.1086/BBLv227n2p201.
9. Zhang G, Fang X, Guo X, Li L, Luo R, Xu F, et al. The oyster genome reveals stress adaptation and complexity of shell formation. Nature. 2012;490:49–54. doi:10.1038/nature11413.
10. Albertin CB, Simakov O, Mitros T, Wang ZY, Pungor JR, Edsinger-Gonzales E, et al. The octopus genome and the evolution of cephalopod neural and morphological novelties. Nature. 2015;524:220–4. doi:10.1038/nature14668.
11. Ren Y, Pan H, Pan B, Bu W. Identification and functional characterization of three TLR signaling pathway genes in *Cyclina sinensis*. Fish Shellfish Immunol. 2016;50:150–9. doi:10.1016/j.fsi.2016.01.025.
12. Adema CM, Hillier LW, Jones CS, Loker ES, Knight M, Minx P, et al. Whole genome analysis of a schistosomiasis-transmitting freshwater snail. Nat Commun. 2017;8:15451. doi:10.1038/ncomms15451.
13. Luo Y-J, Takeuchi T, Koyanagi R, Yamada L, Kanda M, Khalturina M, et al. The *Lingula* genome provides insights into brachiopod evolution and the origin of phosphate biomineralization. Nat Commun. 2015;6:1–10. doi:10.1038/ncomms9301.
14. Laumer CE, Hejnol A, Giribet G. Nuclear genomic signals of the 'microturbellarian' roots of platyhelminth evolutionary innovation. Elife. 2015;4:1–31. doi:10.7554/eLife.05503.
15. Struck TH, Wey-Fabrizius AR, Golombek A, Hering L, Weigert A, Bleidorn C, et al. Platyzoan paraphyly based on phylogenomic data supports a noncoelomate ancestry of Spiralia. Mol Biol Evol. 2014;31:1833–49. doi:10.1093/molbev/msu143.
16. Luo Y-J, Kanda M, Koyanagi R, Hisata K, Akiyama T, Sakamoto H, et al. Nemertean and phoronid genomes reveal lophotrochozoan evolution and the origin of bilaterian heads. Nat Ecol Evol. 2018;2:141–51. doi:10.1038/s41559-017-0389-y.
17. Wasik K, Gurtowski J, Zhou X, Ramos OM, Delás MJ, Battistoni G, et al. Genome and transcriptome of the regeneration-competent flatworm, *Macrostomum lignano*. Proc Natl Acad Sci. 2015;112:12462–7. doi:10.1073/pnas.1516718112.
18. Tsai IJ, Zarowiecki M, Holroyd N, Garciarrubio A, Sanchez-Flores A, Brooks KL, et al. The genomes of four tapeworm species reveal adaptations to parasitism. Nature. 2013;496:57–63. doi:10.1038/nature12031.
19. Zheng L, Zhang L, Lin H, McIntosh MT, Malacrida AR. Toll-like receptors in invertebrate innate immunity. Invertebr Surviv J. 2005;2:105–13.
20. Peiris TH, Hoyer KK, Oviedo NJ. Innate immune system and tissue regeneration in planarians: An area ripe for exploration. Semin Immunol. 2014;26:295–302. doi:10.1016/j.smim.2014.06.005.
21. Eyres I, Boschetti C, Crisp A, Smith TP, Fontaneto D, Tunnacliffe A, et al. Horizontal gene transfer in bdelloid rotifers is ancient, ongoing and more frequent in species from desiccating habitats. BMC Biol. 2015;13:1–17. doi:10.1186/s12915-015-0202-9.
22. Flot J-F, Hespeels B, Li X, Noel B, Arkhipova I, Danchin EGJ, et al. Genomic evidence for ameiotic evolution in the bdelloid rotifer *Adineta vaga*. Nature. 2013;500:453–7.

doi:10.1038/nature12326.
23. Yoshida Y, Koutsovoulos G, Laetsch DR, Stevens L, Kumar S, Horikawa DD, et al. Comparative genomics of the tardigrades *Hypsibius dujardini* and *Ramazzottius varieornatus*. PLOS Biol. 2017;15:e2002266. doi:10.1371/journal.pbio.2002266.
24. Hashimoto T, Horikawa DD, Saito Y, Kuwahara H, Kozuka-Hata H, Shin-I T, et al. Extremotolerant tardigrade genome and improved radiotolerance of human cultured cells by tardigrade-unique protein. Nat Commun. 2016;7:1–14. doi:10.1038/ncomms12808.
25. Sharma PP, Kaluziak ST, Pérez-Porro AR, González VL, Hormiga G, Wheeler WC, et al. Phylogenomic interrogation of arachnida reveals systemic conflicts in phylogenetic signal. Mol Biol Evol. 2014;31:2963–84. doi:10.1093/molbev/msu235.
26. Desjardins CA, Cerqueira GC, Goldberg JM, Dunning Hotopp JC, Haas BJ, Zucker J, et al. Genomics of *Loa loa*, a *Wolbachia*-free filarial parasite of humans. Nat Genet. 2013;45:495–500. doi:10.1038/ng.2585.
27. Cotton JA, Bennuru S, Grote A, Harsha B, Tracey A, Beech R, et al. The genome of *Onchocerca volvulus*, agent of river blindness. Nat Microbiol. 2017;2:16216. doi:10.1038/nmicrobiol.2016.216.
28. Colbourne JK, Pfrender ME, Gilbert D, Thomas WK, Tucker A, Oakley TH, et al. The Ecoresponsive Genome of *Daphnia pulex*. Science. 2011;331:555–61. doi:10.1126/science.1197761.
29. Gulia-Nuss M, Nuss AB, Meyer JM, Sonenshine DE, Roe RM, Waterhouse RM, et al. Genomic insights into the *Ixodes scapularis* tick vector of Lyme disease. Nat Commun. 2016;7:1–13. doi:10.1038/ncomms10507.
30. Sasaki N, Ogasawara M, Sekiguchi T, Kusumoto S, Satake H. Toll-like Receptors of the ascidian *Ciona intestinalis*. J Biol Chem. 2009;284:27336–43. doi:10.1074/jbc.M109.032433.
31. Denoeud F, Henriet S, Mungpakdee S, Aury J-M, Da Silva C, Brinkmann H, et al. Plasticity of animal genome architecture unmasked by rapid evolution of a pelagic tunicate. Science. 2010;330:1381–5. doi:10.1126/science.1194167.
32. Hibino T, Loza-Coll M, Messier C, Majeske AJ, Cohen AH, Terwilliger DP, et al. The immune gene repertoire encoded in the purple sea urchin genome. Dev Biol. 2006;300:349–65. doi:10.1016/j.ydbio.2006.08.065.
