# Supplementary material for: The evolution of the metazoan Toll receptor family and its expression during protostome development
Source: BMC Ecol Evol. 2021 Nov 22;21:208. doi: 10.1186/s12862-021-01927-1 (PMC8609888; doi:10.1186/s12862-021-01927-1)

Additional file 5: Fig. S3 - Third phylogenetic analysis, excluding the 349-354 aminoacid region. Parameters applied for the construction of this phylogenetic tree are the same than the ones applied for the main phylogenetic analysis (Figure 4A). Recovered clades are named α, β and γ. Comparison with the main phylogenetic analysis is represented with blue and magenta dots. Bootstrap values are indicated next to the main nodes and all nodes with bootstrap values >60 are marked with full black dots.

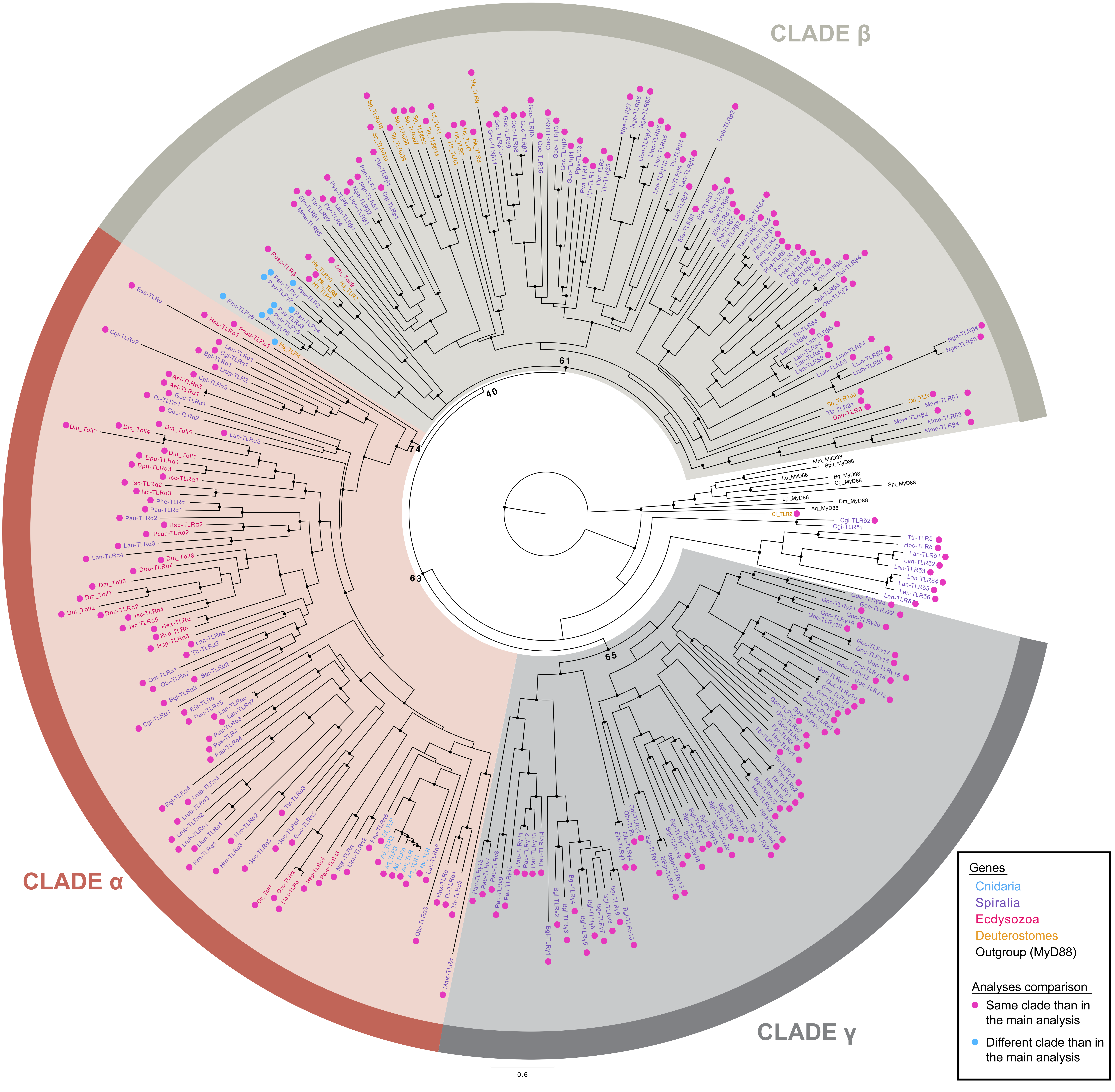

Supplement: Supplementary file 5 — Additional file 5: Fig. S3. Third phylogenetic analysis, excluding the 349–354 aminoacid region. Parameters applied for the construction of this phylogenetic tree are the same than the ones applied for the main phylogenetic analysis (Fig. 4A). Recovered clades are named α, β and γ. Comparison with the main phylogenetic analysis is represented with blue and magenta dots. Bootstrap values are indicated next to the main nodes and all nodes with bootstrap values >60 are marked with full black dots. [file 12862_2021_1927_MOESM5_ESM.pdf]
