# Supplementary material for: The evolution of the metazoan Toll receptor family and its expression during protostome development
Source: BMC Ecol Evol. 2021 Nov 22;21:208. doi: 10.1186/s12862-021-01927-1 (PMC8609888; doi:10.1186/s12862-021-01927-1)
Supplement: Supplementary file 6 — Additional file 6: Table S3. Hypsibius exemplaris stage specific transcriptome analyses (RSEM and Kallisto methods). [file 12862_2021_1927_MOESM6_ESM.pdf]

Additional file 6: Table S3 - *Hypsibius exemplaris* stage specific transcriptome analyses (RSEM and Kallisto methods)

HYPSIBIUS EXEMPLARIS																		
**Values inicate Transcripts per Milion (TEM)**																		
**RSEM**	**Zigot**	**Morula1**	**Morula2**	**Morula3**	**Early gastrula**	**Gastrula 1**	**Gastrula 2**	**Elongation**	**Segmentation 1**	**Segmentation 2**	**Limb bud formation**	**Differentiation 1**	**Differentiation 2**	**Differentiation 3**	**Differentiation 4**	**Differentiation 5**	**Differentiation 6**	**Differentiation 7**	**Differentiation 8**
Hexe-TLRα	3,165	0,000	0,000	8,589	8,071	8,335	1,421	9,685	25,103	0,000	0,000	0,000	27,095	0,000	0,000	0,000	0,000	0,000	0,000
**kallisto**	**Zigot**	**Morula1**	**Morula2**	**Morula3**	**Early gastrula**	**Gastrula 1**	**Gastrula 2**	**Elongation**	**Segmentation 1**	**Segmentation 2**	**Limb bud formation**	**Differentiation 1**	**Differentiation 2**	**Differentiation 3**	**Differentiation 4**	**Differentiation 5**	**Differentiation 6**	**Differentiation 7**	**Differentiation 8**
Hexe-TLRα	0,000	0,000	0,000	27,214	0,000	9,697	0,000	0,000	33,087	0,000	0,000	0,000	77,060	0,000	0,000	0,000	0,000	0,000	0,000

TEM ≥0,150

TEM <0,150
