# Supplementary material for: The evolution of the metazoan Toll receptor family and its expression during protostome development
Source: BMC Ecol Evol. 2021 Nov 22;21:208. doi: 10.1186/s12862-021-01927-1 (PMC8609888; doi:10.1186/s12862-021-01927-1)
Supplement: Supplementary file 7 — Additional file 7: Table S4. Priapulus caudatus stage specific transcriptome analyses. Analyses for the different methods (RSEM and Kallisto) and replicates (Rep 1_and Rep_2). For each method, average and standard error (SE) of the two replicates is provided. [file 12862_2021_1927_MOESM7_ESM.pdf]

Additional file 7: Table S4 - Priapulus caudatus stage specific transcriptome analyses. Analyses for the different methods (RSEM and Kallisto) and replicates (Rep 1_and Rep_2). For each method, average and standard error (SE) of the two replicates is provided.

PRIAPULUS CAUDATUS

Values inicate Transcripts per Milion (TEM)

Rep_1_RSEM	0d	1d	3d	5d	9d
Pcau-TLRα1	2,551	2,294	1,509	1,016	1,512
Pcau-TLRα2	1,471	2,427	1,822	1,983	2,988
Pcau-TLRα3	0,000	0,000	0,822	2,371	5,496

Rep_1_kallisto	0d	1d	3d	5d	9d
Pcau-TLRα1	2,433	2,059	1,487	0,940	1,589
Pcau-TLRα2	1,440	2,093	1,767	1,890	3,089
Pcau-TLRα3	0,000	0,000	0,825	2,156	4,627

Rep_2_RSEM	0d	1d	3d	5d	9d
Pcau-TLRα1	7,824	6,555	3,418	2,230	3,617
Pcau-TLRα2	0,680	2,285	1,477	2,630	5,675
Pcau-TLRα3	0,000	0,000	0,167	1,159	3,396

Rep_2_kallisto	0d	1d	3d	5d	9d
Pcau-TLRα1	7,609	6,107	3,311	1,985	3,445
Pcau-TLRα2	0,724	2,432	1,475	2,469	6,272
Pcau-TLRα3	0,000	0,000	0,189	0,667	3,275

RSEM_average	0d	1d	3d	5d	9d
Pcau-TLRα1	5,188	4,425	2,464	1,623	2,565
Pcau-TLRα2	1,076	2,356	1,650	2,307	4,332
Pcau-TLRα3	0,000	0,000	0,495	1,765	4,446

Kallisto_average	0d	1d	3d	5d	9d
Pcau-TLRα1	5,021	4,083	2,399	1,463	2,517
Pcau-TLRα2	1,082	2,263	1,621	2,180	4,681
Pcau-TLRα3	0,000	0,000	0,507	1,412	3,951

Values indicate Standard Error (SE)

RSEM_SE	0d	1d	3d	5d	9d
Pcau-TLRα1	2,637	2,131	0,955	0,607	1,053
Pcau-TLRα2	0,396	0,071	0,172	0,324	1,344
Pcau-TLRα3	0,000	0,000	0,328	0,606	1,050

Kallisto_SE	0d	1d	3d	5d	9d
Pcau-TLRα1	2,588	2,024	0,912	0,523	0,928
Pcau-TLRα2	0,358	0,170	0,146	0,289	1,592
Pcau-TLRα3	0,000	0,000	0,318	0,745	0,676

TEM ≥0,150

TEM <0,150
