# Supplementary material for: The evolution of the metazoan Toll receptor family and its expression during protostome development
Source: BMC Ecol Evol. 2021 Nov 22;21:208. doi: 10.1186/s12862-021-01927-1 (PMC8609888; doi:10.1186/s12862-021-01927-1)
Supplement: Supplementary file 8 — Additional file 8: Table S5. Crassostrea gigas stage specific transcriptome analyses (RSEM and Kallisto methods). [file 12862_2021_1927_MOESM8_ESM.pdf]

Additional file 8: Table S5 - *Crassostrea gigas* stage specific transcriptome analyses (RSEM and Kallisto methods)

CRASSOSTREA GIGAS

Values inicate Transcripts per Milion (TEM)

**RSEM**	Early morula	Morula	Blastula	Rotary movement	Free swimming	Early gastrula	Gastrula	Trochophore 1	Trochophore 2	Trochophore 3	Trochophore 4	Trochophore 5	early Dshaped larva 1	early Dshaped larva 2	Dshaped larva 1	Dshaped larva 2	Dshaped larva 3	Dshaped larva 4	Dshaped larva 5
Cgi-TLRα1	0,000	0,000	0,163	0,507	0,446	0,136	0,279	0,132	0,758	0,331	0,249	0,646	0,586	0,666	0,371	0,315	0,265	0,279	0,287
Cgi-TLRα2	0,048	0,121	0,154	0,119	0,085	0,027	0,058	0,042	0,000	0,052	0,065	0,055	0,053	0,047	0,000	0,000	0,000	0,052	0,000
Cgi-TLRα3	0,000	0,028	0,029	0,000	0,000	0,000	0,058	0,000	0,124	0,114	0,195	0,109	0,120	0,257	0,379	0,420	0,054	0,279	0,231
Cgi-TLRα4	0,000	0,000	0,000	0,075	0,276	0,345	0,221	0,118	0,310	0,362	0,498	0,777	0,918	0,947	1,190	0,954	1,183	1,311	2,648
Cgi-TLRβ1	0,000	0,000	0,000	0,000	0,032	0,045	0,000	0,000	0,108	0,052	0,000	0,044	0,000	0,047	0,098	0,097	0,048	0,052	0,311
Cgi-TLRβ2	0,019	0,028	0,000	0,000	0,021	0,000	0,000	0,000	0,000	0,052	0,000	0,000	0,000	0,000	0,091	0,000	0,048	0,000	0,191
Cgi-TLRβ3	0,000	0,121	0,029	0,104	0,000	0,000	0,000	0,000	0,000	0,000	0,065	0,000	0,000	0,000	0,053	0,000	0,054	0,000	0,072
Cgi-TLRβ4	0,381	1,021	0,807	0,343	0,266	0,209	0,581	0,439	0,511	0,671	0,217	0,361	0,373	0,175	0,280	0,291	0,088	0,093	0,255
Cgi-TLRγ1	0,000	0,000	0,000	0,000	0,000	0,000	0,000	0,000	0,000	0,300	0,054	0,000	0,093	0,117	0,083	0,040	0,041	0,083	0,000
Cgi-TLRγ2	0,000	0,000	0,000	0,000	0,000	0,000	0,000	0,063	0,093	0,000	0,000	0,077	0,000	0,082	0,159	0,154	0,000	0,083	0,000
Cgi-TLRδ1	0,029	0,000	0,000	0,000	0,000	0,027	0,163	0,042	0,402	0,331	0,260	0,537	0,399	0,666	0,636	0,259	0,734	0,382	0,686
Cgi-TLRδ2	0,877	0,798	0,499	0,418	0,223	0,254	0,221	0,272	0,170	0,186	0,249	0,088	0,333	0,257	0,152	0,267	0,326	0,217	0,295

**kallisto**	Early morula	Morula	Blastula	Rotary movement	Free swimming	Early gastrula	Gastrula	Trochophore 1	Trochophore 2	Trochophore 3	Trochophore 4	Trochophore 5	early Dshaped larva 1	early Dshaped larva 2	Dshaped larva 1	Dshaped larva 2	Dshaped larva 3	Dshaped larva 4	Dshaped larva 5
Cgi-TLRα1	0,000	0,000	0,141	1,011	0,890	0,187	0,671	0,100	1,331	0,772	0,302	1,031	0,821	1,193	0,820	0,457	0,472	0,512	0,384
Cgi-TLRα2	0,058	0,137	0,279	0,143	0,068	0,062	0,133	0,099	0,000	0,127	0,149	0,127	0,135	0,000	0,000	0,000	0,000	0,127	
Cgi-TLRα3	0,000	0,000	0,000	0,000	0,000	0,000	0,000	0,000	0,136	0,262	0,461	0,131	0,139	0,608	0,596	0,816	0,120	0,653	0,392
Cgi-TLRα4	0,000	0,000	0,000	0,190	0,541	0,737	0,353	0,262	0,701	0,508	0,794	1,017	1,441	1,413	1,694	0,752	1,709	2,865	4,888
Cgi-TLRβ1	0,000	0,000	0,000	0,000	0,000	0,000	0,000	0,000	0,119	0,115	0,000	0,000	0,000	0,107	0,000	0,205	0,000	0,115	0,344
Cgi-TLRβ2	0,049	0,000	0,000	0,000	0,058	0,000	0,000	0,000	0,000	0,109	0,000	0,000	0,000	0,000	0,199	0,000	0,100	0,000	0,326
Cgi-TLRβ3	0,000	0,209	0,068	0,270	0,000	0,000	0,000	0,000	0,000	0,000	0,147	0,000	0,000	0,000	0,114	0,000	0,115	0,000	0,124
Cgi-TLRβ4	2,735	2,132	2,365	2,237	1,301	1,388	2,383	1,783	1,924	2,013	1,517	1,440	0,865	1,734	0,615	1,150	0,924	1,002	1,002
Cgi-TLRγ1	0,000	0,000	0,000	0,000	0,000	0,000	0,000	0,000	0,000	0,502	0,118	0,000	0,214	0,186	0,091	0,089	0,092	0,100	0,000
Cgi-TLRγ2	0,000	0,000	0,000	0,000	0,000	0,000	0,000	0,150	0,200	0,000	0,000	0,193	0,000	0,179	0,352	0,343	0,000	0,192	0,000
Cgi-TLRδ1	0,000	0,000	0,000	0,000	0,000	0,000	0,267	0,000	0,794	0,768	0,300	1,025	0,545	1,424	1,048	0,455	1,527	0,765	1,402
Cgi-TLRδ2	1,543	1,722	0,993	0,977	0,618	0,491	0,227	0,506	0,300	0,290	0,595	0,363	0,540	0,538	0,264	0,451	0,532	0,433	0,650

TEM ≥0,150 TEM <0,150
