# Supplementary material for: The evolution of the metazoan Toll receptor family and its expression during protostome development
Source: BMC Ecol Evol. 2021 Nov 22;21:208. doi: 10.1186/s12862-021-01927-1 (PMC8609888; doi:10.1186/s12862-021-01927-1)
Supplement: Supplementary file 9 — Additional file 9: Table S6. Terebratalia transversa stage specific transcriptome analyses. Analyses for the different methods (RSEM and Kallisto) and replicates (Rep 1_and Rep_2). For each method, average and standard error (SE) of the two replicates is provided. [file 12862_2021_1927_MOESM9_ESM.pdf]

Additional file 9 - Table S6 - *Terebratalia transversa* stage specific transcriptome analyses. Analyses for the different methods (RSEM and Kallisto) and replicates (Rep 1_and Rep_2). For each method, average and standard error (SE) of the two replicates is provided.

TEREBRATALIA TRANSVERSA

Values inicate Transcripts per Milion (TEM)

Rep_1_RSEM	oocyte	8 hr mid blastula	19 hr late blastula	24 hr moving blastula	26 hr early gastrula	37 hr mid gastrula	51 hr late gastrula	59 hr bilobed gastrula	68 hr trilobed gastrula	82 hr early larva	98 hr late larva	competent larva (131h)	1 day juvenile	2 day juvenile
**Ttr-TLRα1**	0,000	0,000	0,000	0,000	0,000	0,000	0,000	0,000	0,000	0,000	0,000	0,000	0,000	0,000
**Ttr-TLRα2**	0,290	0,216	0,189	0,104	0,058	0,028	0,028	0,064	0,116	0,139	0,062	1,570	0,359	0,600
**Ttr-TLRα3**	0,000	0,000	0,000	0,088	0,099	0,000	0,000	0,000	0,000	0,000	0,000	2,302	1,983	2,589
**Ttr-TLRα4**	0,807	0,059	0,074	0,249	0,214	0,047	0,000	0,000	0,412	0,492	1,123	2,691	4,488	2,578
**Ttr-TLRα5**	0,105	0,137	0,041	0,064	0,074	0,056	0,120	0,100	0,245	0,177	0,072	0,071	0,098	0,235
**Ttr-TLRβ1**	0,129	0,000	0,000	0,096	0,222	0,150	0,000	0,000	0,039	0,253	0,577	0,708	0,153	0,000
**Ttr-TLRβ2**	0,524	0,634	0,287	0,329	0,330	0,122	0,240	0,237	0,155	0,164	0,546	0,342	0,065	0,071
**Ttr-TLRβ3**	0,331	0,471	0,771	0,553	0,577	0,207	0,065	0,428	0,142	0,076	0,330	0,224	0,458	0,271
**Ttr-TLRβ4**	0,000	0,000	0,090	0,048	0,346	0,197	0,037	0,391	0,567	0,076	0,824	0,602	0,381	0,294
**Ttr-TLRβ5**	0,000	0,039	0,066	0,160	0,058	0,066	0,000	0,000	0,000	0,000	0,000	0,059	0,000	0,000
**Ttr-TLRγ1**	0,000	0,000	0,000	0,000	0,000	0,000	0,092	0,064	0,000	0,000	0,000	0,000	0,501	0,094
**Ttr-TLRγ2**	0,000	0,000	0,000	0,000	0,000	0,000	0,000	0,000	0,000	0,000	0,000	0,106	0,000	0,282
**Ttr-TLRγ3**	0,000	0,000	0,000	0,000	0,000	0,000	0,000	0,000	0,000	0,000	0,000	0,000	0,000	0,000
**Ttr-TLRγ4**	0,226	0,275	0,082	0,192	0,568	0,442	0,406	0,309	0,142	0,088	0,227	0,236	0,458	0,294
**Ttr-TLRδ**	0,024	0,020	0,057	0,152	0,148	0,160	0,000	0,064	0,052	0,126	0,165	0,224	0,033	0,000

Rep_2_RSEM	oocyte	8 hr mid blastula	19 hr late blastula	24 hr moving blastula	26 hr early gastrula	37 hr mid gastrula	51 hr late gastrula	59 hr bilobed gastrula	68 hr trilobed gastrula	82 hr early larva	98 hr late larva	competent larva (131h)	1 day juvenile	2 day juvenile
**Ttr-TLRα1**	0,000	0,000	0,000	0,000	0,000	0,000	0,000	0,000	0,000	0,000	0,000	0,000	0,000	0,000
**Ttr-TLRα2**	0,121	0,161	0,207	0,222	0,080	0,116	0,034	0,035	0,157	0,343	0,229	0,668	0,639	0,403
**Ttr-TLRα3**	0,000	0,000	0,057	0,000	0,000	0,045	0,000	0,000	0,000	0,000	0,000	0,196	1,943	1,327
**Ttr-TLRα4**	0,664	0,484	0,607	0,625	1,045	0,695	0,051	0,129	0,868	0,520	0,774	0,701	3,791	5,293
**Ttr-TLRα5**	0,121	0,101	0,136	0,277	0,195	0,196	0,488	0,493	1,129	0,213	0,229	0,163	0,326	0,522
**Ttr-TLRβ1**	0,113	0,000	0,064	0,000	0,000	0,062	0,000	0,000	0,220	0,106	0,264	0,342	0,163	0,030

**Ttr-TLRβ2**	0,089	0,040	0,236	0,087	0,062	0,018	0,069	0,082	0,366	0,095	0,035	0,065	0,122	0,000
**Ttr-TLRβ3**	0,810	0,471	0,743	0,688	0,789	0,740	0,950	0,258	1,098	0,118	0,211	0,261	0,353	0,268
**Ttr-TLRβ4**	0,000	0,000	0,043	0,111	0,000	0,000	0,000	0,000	0,063	0,000	0,000	0,000	0,000	0,000
**Ttr-TLRβ5**	0,000	0,000	0,036	0,222	0,000	0,000	0,077	0,000	0,000	0,000	0,000	0,000	0,000	0,119
**Ttr-TLRγ1**	0,000	0,034	0,000	0,000	0,000	0,053	0,103	0,000	0,000	0,000	0,000	0,000	0,000	0,000
**Ttr-TLRγ2**	0,000	0,000	0,036	0,000	0,000	0,000	0,000	0,000	0,209	0,000	0,000	0,000	0,000	0,000
**Ttr-TLRγ3**	0,000	0,000	0,000	0,000	0,000	0,027	0,000	0,000	0,000	0,000	0,000	0,000	0,000	0,000
**Ttr-TLRγ4**	0,121	0,195	0,129	0,182	0,106	0,134	0,325	0,317	0,324	0,154	0,457	0,326	0,476	0,656
**Ttr-TLRδ**	0,000	0,040	0,279	0,649	0,230	0,160	0,394	0,047	0,178	0,035	0,070	0,065	0,190	0,313

**RSEM_average**	**oocyte**	**8 hr mid blastula**	**19 hr late blastula**	**24 hr moving blastula**	**26 hr early gastrula**	**37 hr mid gastrula**	**51 hr late gastrula**	**59 hr bilobed gastrula**	**68 hr trilobed gastrula**	**82 hr early larva**	**98 hr late larva**	**competent larva (131h)**	**1 day juvenile**	**2 day juvenile**
**Ttr-TLRα1**	0,000	0,000	0,000	0,000	0,000	0,000	0,000	0,000	0,000	0,000	0,000	0,000	0,000	0,000
**Ttr-TLRα2**	0,206	0,189	0,198	0,163	0,069	0,072	0,031	0,050	0,137	0,241	0,146	1,119	0,499	0,502
**Ttr-TLRα3**	0,000	0,000	0,029	0,044	0,050	0,023	0,000	0,000	0,000	0,000	0,000	1,249	1,963	1,958
**Ttr-TLRα4**	0,736	0,272	0,341	0,437	0,630	0,371	0,026	0,065	0,640	0,506	0,949	1,696	4,140	3,936
**Ttr-TLRα5**	0,113	0,119	0,089	0,171	0,135	0,126	0,304	0,297	0,687	0,195	0,151	0,117	0,212	0,379
**Ttr-TLRβ1**	0,121	0,000	0,032	0,048	0,111	0,106	0,000	0,000	0,130	0,180	0,421	0,525	0,158	0,015
**Ttr-TLRβ2**	0,307	0,337	0,262	0,208	0,196	0,070	0,155	0,160	0,261	0,130	0,291	0,204	0,094	0,036
**Ttr-TLRβ3**	0,571	0,471	0,757	0,621	0,683	0,474	0,508	0,343	0,620	0,097	0,271	0,243	0,406	0,270
**Ttr-TLRβ4**	0,000	0,000	0,067	0,080	0,173	0,099	0,019	0,196	0,315	0,038	0,412	0,301	0,191	0,147
**Ttr-TLRβ5**	0,000	0,020	0,051	0,191	0,029	0,033	0,039	0,000	0,000	0,000	0,000	0,030	0,000	0,060
**Ttr-TLRγ1**	0,000	0,017	0,000	0,000	0,000	0,027	0,098	0,032	0,000	0,000	0,000	0,000	0,251	0,047
**Ttr-TLRγ2**	0,000	0,000	0,018	0,000	0,000	0,000	0,000	0,000	0,105	0,000	0,000	0,053	0,000	0,141
**Ttr-TLRγ3**	0,000	0,000	0,000	0,000	0,000	0,014	0,000	0,000	0,000	0,000	0,000	0,000	0,000	0,000
**Ttr-TLRγ4**	0,174	0,235	0,106	0,187	0,337	0,288	0,366	0,313	0,233	0,121	0,342	0,281	0,467	0,475
**Ttr-TLRδ**	0,012	0,030	0,168	0,401	0,189	0,160	0,197	0,056	0,115	0,081	0,118	0,145	0,112	0,157

TEM ≥0,150
TEM <0,150

Values indicate Standard Error (SE)

RSEM_SE	oocyte	8 hr mid blastula	19 hr late blastula	24 hr moving blastula	26 hr early gastrula	37 hr mid gastrula	51 hr late gastrula	59 hr bilobed gastrula	68 hr trilobed gastrula	82 hr early larva	98 hr late larva	competent larva (131h)	1 day juvenile	2 day juvenile
**Ttr-TLRα1**	0,000	0,000	0,000	0,000	0,000	0,000	0,000	0,000	0,000	0,000	0,000	0,000	0,000	0,000
**Ttr-TLRα2**	0,085	0,028	0,009	0,059	0,011	0,044	0,003	0,015	0,020	0,102	0,084	0,451	0,140	0,098
**Ttr-TLRα3**	0,000	0,000	0,029	0,044	0,050	0,023	0,000	0,000	0,000	0,000	0,000	1,053	0,020	0,631
**Ttr-TLRα4**	0,072	0,213	0,267	0,188	0,416	0,324	0,026	0,065	0,228	0,014	0,175	0,995	0,349	1,358
**Ttr-TLRα5**	0,008	0,018	0,048	0,107	0,061	0,070	0,184	0,197	0,442	0,018	0,079	0,046	0,114	0,144
**Ttr-TLRβ1**	0,008	0,000	0,032	0,048	0,111	0,044	0,000	0,000	0,091	0,074	0,157	0,183	0,005	0,015
**Ttr-TLRβ2**	0,218	0,297	0,026	0,121	0,134	0,052	0,086	0,078	0,106	0,035	0,256	0,139	0,029	0,036
**Ttr-TLRβ3**	0,240	0,000	0,014	0,067	0,106	0,267	0,443	0,085	0,478	0,021	0,059	0,019	0,053	0,002
**Ttr-TLRβ4**	0,000	0,000	0,024	0,032	0,173	0,099	0,019	0,196	0,252	0,038	0,412	0,301	0,191	0,147
**Ttr-TLRβ5**	0,000	0,020	0,015	0,031	0,029	0,033	0,039	0,000	0,000	0,000	0,000	0,030	0,000	0,060
**Ttr-TLRγ1**	0,000	0,017	0,000	0,000	0,000	0,027	0,006	0,032	0,000	0,000	0,000	0,000	0,251	0,047
**Ttr-TLRγ2**	0,000	0,000	0,018	0,000	0,000	0,000	0,000	0,000	0,105	0,000	0,000	0,053	0,000	0,141
**Ttr-TLRγ3**	0,000	0,000	0,000	0,000	0,000	0,014	0,000	0,000	0,000	0,000	0,000	0,000	0,000	0,000
**Ttr-TLRγ4**	0,053	0,040	0,023	0,005	0,231	0,154	0,040	0,004	0,091	0,033	0,115	0,045	0,009	0,181
**Ttr-TLRδ**	0,012	0,010	0,111	0,249	0,041	0,000	0,197	0,008	0,063	0,046	0,048	0,080	0,079	0,157

Rep_1_kallisto	oocyte	8 hr mid blastula	19 hr late blastula	24 hr moving blastula	26 hr early gastrula	37 hr mid gastrula	51 hr late gastrula	59 hr bilobed gastrula	68 hr trilobed gastrula	82 hr early larva	98 hr late larva	competent larva (131h)	1 day juvenile	2 day juvenile
**Ttr-TLRα1**	0,000	0,000	0,000	0,000	0,000	0,000	0,000	0,000	0,000	0,000	0,006	0,000	0,000	0,000
**Ttr-TLRα2**	0,300	0,139	0,162	0,084	0,059	0,034	0,032	0,063	0,127	0,114	0,031	1,694	0,342	0,638
**Ttr-TLRα3**	0,000	0,000	0,000	0,000	0,068	0,000	0,000	0,000	0,000	0,000	0,042	2,292	1,974	2,754
**Ttr-TLRα4**	0,726	0,074	0,125	0,249	0,224	0,043	0,000	0,000	0,335	0,481	0,913	2,408	3,993	2,758
**Ttr-TLRα5**	0,076	0,109	0,020	0,064	0,054	0,061	0,119	0,096	0,194	0,186	0,056	0,107	0,057	0,225
**Ttr-TLRβ1**	0,099	0,101	0,001	0,095	0,004	0,000	0,000	0,000	0,000	0,237	0,000	0,628	0,004	0,008
**Ttr-TLRβ2**	0,490	0,517	0,228	0,301	0,248	0,071	0,137	0,172	0,089	0,133	0,560	0,312	0,065	0,064
**Ttr-TLRβ3**	0,313	0,398	0,737	0,463	0,360	0,185	0,089	0,408	0,145	0,000	0,252	0,252	0,517	0,225
**Ttr-TLRβ4**	0,000	0,038	0,000	0,062	0,371	0,460	0,063	0,624	0,630	0,408	0,767	0,568	0,285	0,309
**Ttr-TLRβ5**	0,000	0,045	0,000	0,140	0,066	0,075	0,000	0,000	0,000	0,000	0,000	0,065	0,000	0,000
**Ttr-TLRγ1**	0,000	0,000	0,127	0,041	0,249	0,144	0,316	0,061	0,000	0,000	0,000	0,057	0,071	0,136

**Ttr-TLRγ2**	0,000	0,000	0,000	0,000	0,000	0,000	0,000	0,000	0,000	0,000	0,000	0,000	0,000	0,188
**Ttr-TLRγ3**	0,000	0,000	0,000	0,000	0,000	0,000	0,000	0,000	0,000	0,000	0,000	0,000	0,000	0,000
**Ttr-TLRγ4**	0,211	0,228	0,039	0,176	0,551	0,404	0,371	0,283	0,115	0,092	0,204	0,176	0,393	0,304
**Ttr-TLRδ**	0,000	0,020	0,038	0,135	0,200	0,192	0,000	0,042	0,079	0,209	0,183	0,281	0,000	0,000

**Rep_2_kallisto**	**oocyte**	**8 hr mid blastula**	**19 hr late blastula**	**24 hr moving blastula**	**26 hr early gastrula**	**37 hr mid gastrula**	**51 hr late gastrula**	**59 hr bilobed gastrula**	**68 hr trilobed gastrula**	**82 hr early larva**	**98 hr late larva**	**competent larva (131h)**	**1 day juvenile**	**2 day juvenile**
**Ttr-TLRα1**	0,000	0,000	0,000	0,019	0,000	0,000	0,000	0,000	0,000	0,000	0,021	0,027	0,000	0,000
**Ttr-TLRα2**	0,143	0,165	0,184	0,150	0,083	0,120	0,037	0,037	0,157	0,364	0,194	0,578	0,690	0,443
**Ttr-TLRα3**	0,000	0,000	0,058	0,000	0,000	0,000	0,105	0,000	0,000	0,000	0,000	0,167	2,062	1,534
**Ttr-TLRα4**	0,466	0,403	0,583	0,849	0,905	0,667	0,129	0,214	0,865	0,347	0,730	0,630	3,468	5,164
**Ttr-TLRα5**	0,116	0,090	0,133	0,252	0,186	0,159	0,430	0,401	1,017	0,222	0,207	0,147	0,312	0,618
**Ttr-TLRβ1**	0,094	0,001	0,047	0,003	0,001	0,045	0,000	0,000	0,226	0,001	0,192	0,354	0,130	0,000
**Ttr-TLRβ2**	0,082	0,025	0,235	0,079	0,058	0,014	0,065	0,076	0,314	0,116	0,034	0,069	0,140	0,086
**Ttr-TLRβ3**	0,626	0,416	0,633	0,515	0,671	0,562	0,778	0,221	0,927	0,086	0,076	0,183	0,199	0,000
**Ttr-TLRβ4**	0,000	0,000	0,060	0,000	0,000	0,119	0,000	0,000	0,172	0,071	0,000	0,170	0,080	0,000
**Ttr-TLRβ5**	0,000	0,000	0,038	0,253	0,000	0,000	0,042	0,000	0,000	0,000	0,000	0,000	0,000	0,000
**Ttr-TLRγ1**	0,000	0,000	0,033	0,000	0,000	0,088	0,145	0,000	0,000	0,000	0,000	0,000	0,000	0,123
**Ttr-TLRγ2**	0,000	0,000	0,000	0,000	0,000	0,000	0,000	0,000	0,174	0,000	0,000	0,000	0,000	0,000
**Ttr-TLRγ3**	0,000	0,000	0,000	0,000	0,000	0,000	0,000	0,000	0,000	0,000	0,000	0,000	0,000	0,000
**Ttr-TLRγ4**	0,100	0,166	0,131	0,113	0,100	0,109	0,279	0,264	0,256	0,159	0,321	0,348	0,477	0,572
**Ttr-TLRδ**	0,000	0,026	0,255	0,657	0,327	0,120	0,512	0,091	0,460	0,000	0,054	0,216	0,201	0,287

**Kallisto_average**	**oocyte**	**8 hr mid blastula**	**19 hr late blastula**	**24 hr moving blastula**	**26 hr early gastrula**	**37 hr mid gastrula**	**51 hr late gastrula**	**59 hr bilobed gastrula**	**68 hr trilobed gastrula**	**82 hr early larva**	**98 hr late larva**	**competent larva (131h)**	**1 day juvenile**	**2 day juvenile**
**Ttr-TLRα1**	0,000	0,000	0,000	0,010	0,000	0,000	0,000	0,000	0,000	0,000	0,014	0,014	0,000	0,000
**Ttr-TLRα2**	0,222	0,152	0,173	0,117	0,071	0,077	0,035	0,050	0,142	0,239	0,113	1,136	0,516	0,541
**Ttr-TLRα3**	0,000	0,000	0,029	0,000	0,034	0,000	0,053	0,000	0,000	0,000	0,021	1,230	2,018	2,144
**Ttr-TLRα4**	0,596	0,239	0,354	0,549	0,565	0,355	0,065	0,107	0,600	0,414	0,822	1,519	3,731	3,961
**Ttr-TLRα5**	0,096	0,100	0,077	0,158	0,120	0,110	0,275	0,249	0,606	0,204	0,132	0,127	0,185	0,422
**Ttr-TLRβ1**	0,097	0,051	0,024	0,049	0,003	0,023	0,000	0,000	0,113	0,119	0,096	0,491	0,067	0,004
**Ttr-TLRβ2**	0,286	0,271	0,232	0,190	0,153	0,043	0,101	0,124	0,202	0,125	0,297	0,191	0,103	0,075
**Ttr-TLRβ3**	0,470	0,407	0,685	0,489	0,516	0,374	0,434	0,315	0,536	0,043	0,164	0,218	0,358	0,113
**Ttr-TLRβ4**	0,000	0,019	0,030	0,031	0,186	0,290	0,032	0,312	0,401	0,240	0,384	0,369	0,183	0,155
**Ttr-TLRβ5**	0,000	0,023	0,019	0,197	0,033	0,038	0,021	0,000	0,000	0,000	0,000	0,033	0,000	0,000

**Ttr-TLRγ1**	0,000	0,000	0,080	0,021	0,125	0,116	0,231	0,031	0,000	0,000	0,000	0,029	0,036	0,130
**Ttr-TLRγ2**	0,000	0,000	0,000	0,000	0,000	0,000	0,000	0,000	0,087	0,000	0,000	0,000	0,000	0,094
**Ttr-TLRγ3**	0,000	0,000	0,000	0,000	0,000	0,000	0,000	0,000	0,000	0,000	0,000	0,000	0,000	0,000
**Ttr-TLRγ4**	0,156	0,197	0,085	0,145	0,326	0,257	0,325	0,274	0,186	0,126	0,263	0,262	0,435	0,438
**Ttr-TLRδ**	0,000	0,023	0,147	0,396	0,264	0,156	0,256	0,067	0,270	0,105	0,119	0,249	0,101	0,144

TEM ≥0,150
TEM <0,150

**RSEM_Standard error**	**oocyte**	**8 hr mid blastula**	**19 hr late blastula**	**24 hr moving blastula**	**26 hr early gastrula**	**37 hr mid gastrula**	**51 hr late gastrula**	**59 hr bilobed gastrula**	**68 hr trilobed gastrula**	**82 hr early larva**	**98 hr late larva**	**competent larva (131h)**	**1 day juvenile**	**2 day juvenile**
**Ttr-TLRα1**	0,000	0,000	0,000	0,010	0,000	0,000	0,000	0,000	0,000	0,000	0,008	0,014	0,000	0,000
**Ttr-TLRα2**	0,079	0,013	0,011	0,033	0,012	0,043	0,003	0,013	0,015	0,125	0,082	0,558	0,174	0,098
**Ttr-TLRα3**	0,000	0,000	0,029	0,000	0,034	0,000	0,053	0,000	0,000	0,000	0,021	1,063	0,044	0,610
**Ttr-TLRα4**	0,130	0,165	0,229	0,300	0,341	0,312	0,065	0,107	0,265	0,067	0,091	0,889	0,263	1,203
**Ttr-TLRα5**	0,020	0,010	0,057	0,094	0,066	0,049	0,156	0,153	0,412	0,018	0,076	0,020	0,128	0,197
**Ttr-TLRβ1**	0,003	0,050	0,023	0,046	0,002	0,023	0,000	0,000	0,113	0,118	0,096	0,137	0,063	0,004
**Ttr-TLRβ2**	0,204	0,246	0,003	0,111	0,095	0,029	0,036	0,048	0,113	0,009	0,263	0,122	0,038	0,011
**Ttr-TLRβ3**	0,157	0,009	0,052	0,026	0,156	0,189	0,345	0,093	0,391	0,043	0,088	0,035	0,159	0,113
**Ttr-TLRβ4**	0,000	0,019	0,030	0,031	0,186	0,171	0,032	0,312	0,229	0,169	0,384	0,199	0,103	0,155
**Ttr-TLRβ5**	0,000	0,023	0,019	0,057	0,033	0,038	0,021	0,000	0,000	0,000	0,000	0,033	0,000	0,000
**Ttr-TLRγ1**	0,000	0,000	0,047	0,021	0,125	0,028	0,086	0,031	0,000	0,000	0,000	0,029	0,036	0,007
**Ttr-TLRγ2**	0,000	0,000	0,000	0,000	0,000	0,000	0,000	0,000	0,087	0,000	0,000	0,000	0,000	0,094
**Ttr-TLRγ3**	0,000	0,000	0,000	0,000	0,000	0,000	0,000	0,000	0,000	0,000	0,000	0,000	0,000	0,000
**Ttr-TLRγ4**	0,056	0,031	0,046	0,032	0,226	0,148	0,046	0,009	0,071	0,034	0,059	0,086	0,042	0,134
**Ttr-TLRδ**	0,000	0,003	0,109	0,261	0,064	0,036	0,256	0,025	0,191	0,105	0,065	0,033	0,101	0,144
