# Supplementary material for: The evolution of the metazoan Toll receptor family and its expression during protostome development
Source: BMC Ecol Evol. 2021 Nov 22;21:208. doi: 10.1186/s12862-021-01927-1 (PMC8609888; doi:10.1186/s12862-021-01927-1)

Additional file 10: Fig. S4 – RNAse treatment experiment followed by *in situ* hybridization for the *Ttr-TLRα4* gene. A. RNAse treated animals only show the ring-shaped staining (white asterisk). B. Control animals show expression of the *Ttr-TLRα4* gene in the brain and the pedicle (blue arrows) and the ring-shaped staining (white asterisk). Therefore, as the ring-shaped staining is present in RNAse treated specimens, we conclude that it is non-specific staining.

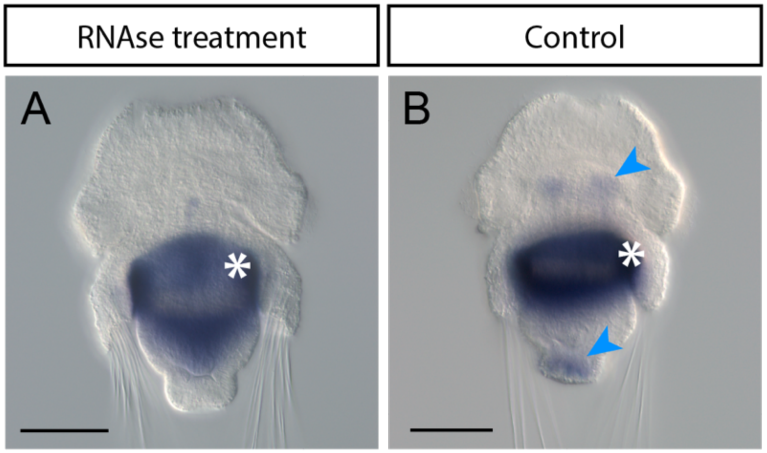

Supplement: Supplementary file 10 — Additional file 10: Fig. S4. RNAse treatment experiment followed by in situ hybridization for the Ttr-TLRα4 gene. A. RNAse treated animals only show the ring-shaped staining (white asterisk). B. Control animals show expression of the Ttr-TLRα4 gene in the brain and the pedicle (blue arrows) and the ring-shaped staining (white asterisk). Therefore, as the ring-shaped staining is present in RNAse treated specimens, we conclude that it is nonspecific staining. [file 12862_2021_1927_MOESM10_ESM.pdf]
