# Supplementary material for: The evolution of the metazoan Toll receptor family and its expression during protostome development
Source: BMC Ecol Evol. 2021 Nov 22;21:208. doi: 10.1186/s12862-021-01927-1 (PMC8609888; doi:10.1186/s12862-021-01927-1)

Additional file 11: Fig.S5 – Different scenarios for TLR evolution. Our phylogenetic analysis suggests different hypothesis about when the duplications that originated the ancestral genes for clades α, β and γ occurred. First, we can hypothesize that in the planulozoan common ancestor there were present either one (*TLR-Cα/β/γ*) – hypothesis 1A – (panels A,C and E) or two TLRs – hypothesis 1B – (panels B, D and F). Second, the duplication of *TLR-Cβ/γ* that gave rise to *TLR-Cβ* and *TLR-Cγ* could have occurred either in the nephrozoan common ancestor – hypothesis 2A – (panels A and B), the spiralian common ancestor – hypothesis 2B – (panels C and D) or the trochozoan common ancestor – hypothesis 2C – (panels E and F). The panels in this figure show the different combinations of these hypotheses.

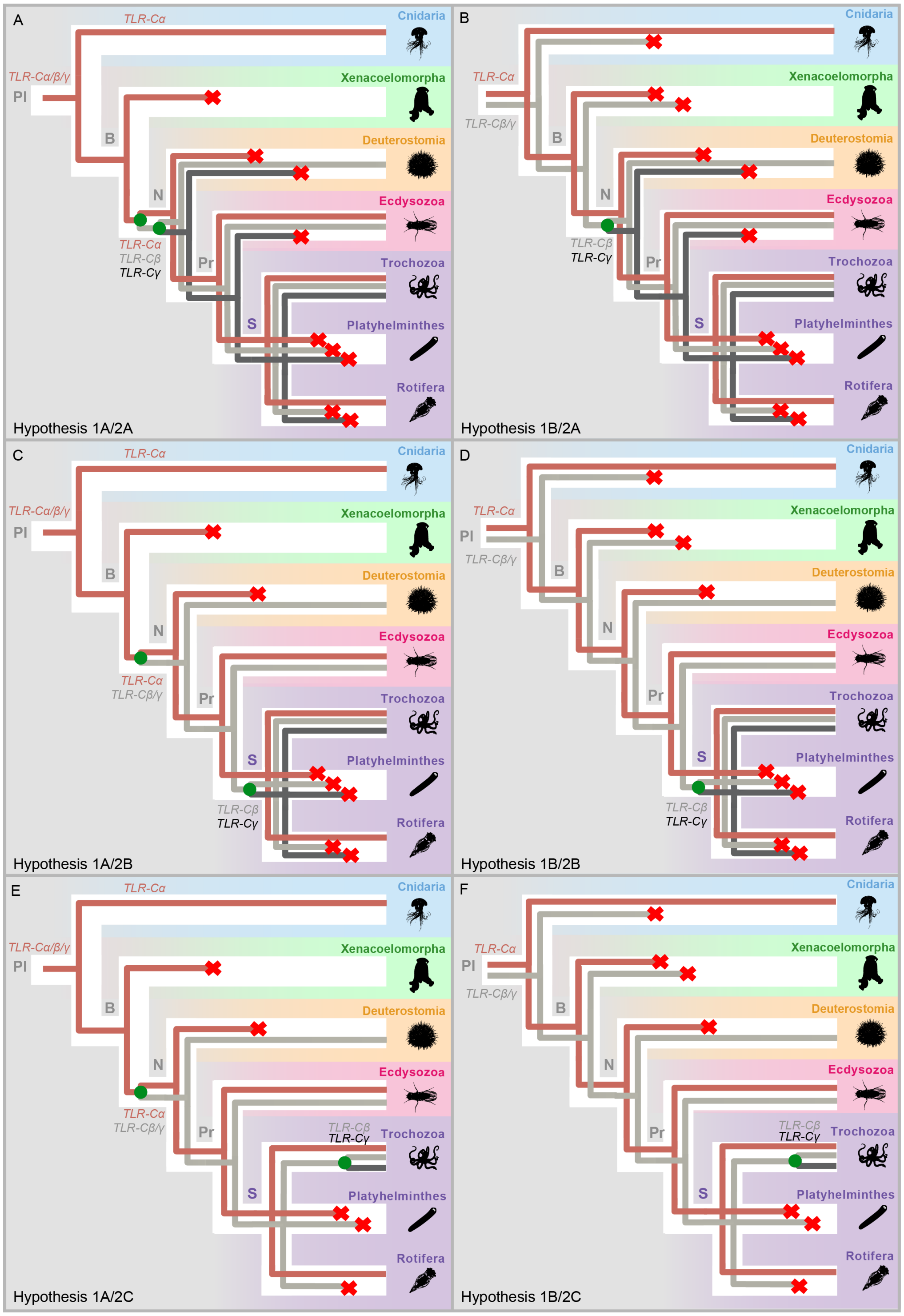

Supplement: Supplementary file 11 — Additional file 11: Fig. S5. Different scenarios for TLR evolution. Our phylogenetic analysis suggests different hypothesis about when the duplications that originated the ancestral genes for clades α, β and γ occurred. First, we can hypothesize that in the planulozoan common ancestor there were present either one (TLR-Cα/β/γ)—hypothesis 1A – (panels A, C and E) or two TLRs – hypothesis 1B—(panels B, D and F). Second, the duplication of TLR-Cβ/γ that gave raise to TLR-Cβ and TLR-Cγ could have occurred either in the nephrozoan common ancestor—hypothesis 2A–(panels A and B), the spiralian common ancestor – hypothesis 2B—(panels C and D) or the trochozoan common ancestor—hypothesis 2C—(panels E and F). The panels in this figure show the different combinations of these hypotheses. [file 12862_2021_1927_MOESM11_ESM.pdf]
